# The evolution of TNF signaling in platyhelminths suggests the cooptation of TNF receptor in the host-parasite interplay

**DOI:** 10.1186/s13071-020-04370-1

**Published:** 2020-09-25

**Authors:** Claudio R. Bertevello, Bruno R. A. Russo, Ana C. Tahira, Ednilson Hilário Lopes-Junior, Ricardo DeMarco, Katia C. Oliveira

**Affiliations:** 1 Departamento de Microbiologia, Imunologia e Parasitologia, Escola Paulista de Medicina, Universidade Federal de São Paulo, São Paulo, Brazil; 2 Departamento de Psiquiatria, Faculdade de Medicina, Universidade de São Paulo, São Paulo, Brazil; 3 Instituto de Física de São Carlos, Universidade de São Paulo, São Carlos, Brazil

**Keywords:** Signal transduction, Host-parasite relationship, TNF-α receptors and ligand, Platyhelminths, Molecular crosstalk

## Abstract

**Background:**

The TNF signaling pathway is involved in the regulation of many cellular processes (such as apoptosis and cell proliferation). Previous reports indicated the effect of human TNF-α on metabolism, physiology, gene expression and protein phosphorylation of the human parasite *Schistosoma mansoni* and suggested that its TNF receptor was responsible for this response. The lack of an endogenous TNF ligand reinforced the idea of the use of an exogenous ligand, but also opens the possibility that the receptor actually binds a non-canonical ligand, as observed for NGFRs.

**Methods:**

To obtain a more comprehensive view, we analyzed platyhelminth genomes deposited in the Wormbase ParaSite database to investigate the presence of TNF receptors and their respective ligands. Using different bioinformatics approaches, such as HMMer and BLAST search tools we identified and characterized the sequence of TNF receptors and ligand homologs. We also used bioinformatics resources for the identification of conserved protein domains and Bayesian inference for phylogenetic analysis.

**Results:**

Our analyses indicate the presence of 31 TNF receptors in 30 platyhelminth species. All platyhelminths display a single TNF receptor, and all are structurally remarkably similar to NGFR. It suggests no events of duplication and diversification occurred in this phylum, with the exception of a single species-specific duplication. Interestingly, we also identified TNF ligand homologs in five species of free-living platyhelminths.

**Conclusions:**

These results suggest that the TNF receptor from platyhelminths may be able to bind canonical TNF ligands, thus strengthening the idea that these receptors are able to bind human TNF-α. This also raises the hypothesis that an endogenous ligand was substituted by the host ligand in parasitic platyhelminths. Moreover, our analysis indicates that death domains (DD) may be present in the intracellular region of most platyhelminth TNF receptors, thus pointing to a previously unreported apoptotic action of such receptors in platyhelminths. Our data highlight the idea that host-parasite crosstalk using the TNF pathway may be widespread in parasitic platyhelminths to mediate apoptotic responses. This opens up a new hypothesis to uncover what might be an important component to understand platyhelminth infections.
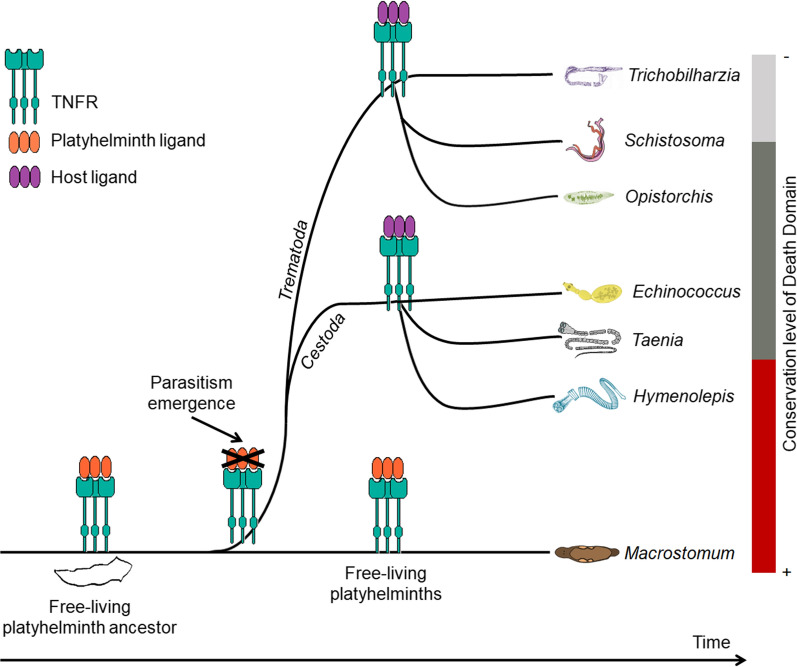

## Introduction

Infections caused by platyhelminths are spread worldwide; three out of 17 neglected tropical diseases have platyhelminths as the etiological agent (schistosomiasis, taeniasis/cysticercosis and echinococcosis) and together affect millions of people and cause thousands of deaths annually [1–4]. The host-parasite interplay is an intricate relation involving the exchange of several molecular signals and signaling pathways that are responsible for the parasite adaptation to the different environments along its biological life-cycle [5].

TNF-α is an early pro-inflammatory cytokine produced by several cell types in mammals [6]. The literature has described several effects of human TNF-α on *Schistosoma mansoni*. Pioneer studies of infection with *S. mansoni* in severe combined immunodeficient (SCID) mice showed a diminished egg-laying that was only restored with exposure to recombinant TNF-α in adult worms [7]. Controversially, further reports described directly the *in vitro* action of TNF-α in adult worm metabolism leading to a decrease of egg-laying, methionine and tyrosine uptake in females [8–10]. Moreover, further experiments with SCID mice suggested a delay, rather than an impairment of egg-laying and that TNF-α levels were not diminished in that model and, therefore, play no role in alteration of egg-laying patterns [11]. Indeed, experiments using TNF^−/−^ mice indicated no influence on egg-laying *in vivo*, but suggested that TNF deficiency results in attrition of schistosomes in the portal system [12]. Direct *in vitro* action of human TNF-α in *S. mansoni* was also reported to result in changes in the gene expression profile of adult worms [13] and in the phosphorylation profile of male adult worm proteins [14]. Therefore, although several experiments suggest an effect of TNF-α in *S. mansoni* metabolism, the nature and extent of such influence remain poorly understood.

With the advances of next-generation sequencing (NGS) techniques several genomes and transcriptomes of parasites have been sequenced and analyzed [15–19]. As a consequence, the amount of sequences available on public databases has been increasing during recent years and many specialized databases for pathogens/parasites have emerged. These resources allowed a comprehensive evaluation of diverse molecular systems in platyhelminths [20, 21]. The use of such databases made possible the characterization of signal transduction pathway components such as membrane receptors [22, 23], protein kinases [24, 25] and transcription factors [26, 27], among others. One of these receptors, *Sm*TNFR [13], is particularly interesting because this is the most likely molecular candidate to mediate the previously described effects of human TNF-α on *S. mansoni* (the etiological agent of schistosomiasis). Phylogenetic analysis of TNFR from *S. mansoni* suggested a basal position in relation to TNFR1, TNFR2 and CD40 families in vertebrates, which are able to bind *bona fide* trimeric TNF ligands. However, the bootstrap support for such a classification was only moderate and the structure of the *S. mansoni* TNFR was also similar to nerve growth factor (NGF) receptors [13], which are members of the TNFR family but have no known *bona fide* TNF ligands. The absence of described endogenous *bona fide* TNF ligand in platyhelminths and the fact that several platyhelminths are free-living (not exposed to TNF ligands from a mammalian host) raise questions about whether these receptors would function by binding non-canonical ligands, as observed by nerve growth factor receptors (NGFRs).

All these findings reveal how the molecular crosstalk between parasite and host is important and can modulate several biological processes in the parasite. To investigate if the TNF-α signaling may have a molecular role in other organisms, we aimed to identify and characterize *Sm*TNFR homologs and their eventual ligands in platyhelminths, using the available sequences in the public databases.

## Methods

### Identification of homologs of *Sm*TNFR and TNF ligands

Two complementary approaches were used to identify the possible homologous genes of *Sm*TNFR: BLAST (v.2.10.0) [28] and HMMER (v.3.3) [29] as described as follows. The first approach used the *Sm*TNFR sequence as a query to perform a BLASTp (v.2.10.0) at public sequence databases, GenBank (https://www.ncbi.nlm.nih.gov/genbank/) and Gene DB (https://www.genedb.org/). As result, we were able to identify homologs in 7 organisms: the platyhelminths *Echinococcus granulosus* (gene ID EgrG 000990500); *E. multilocularis* (gene ID EmuJ 000990500); *Hymenolepis microstoma* (HmN 000322000); *Taenia solium* (gene ID TsM 000678000); *Clonorchis sinensis* (gene ID GAA49741.1); *Opisthorchis viverrini* (gene ID XM 009173815.1) and the nematode *Trichinella spiralis* (gene ID XP 003371690.1). No homologous sequences were found among protozoan sequence data.

For the second approach, we used all previously identified helminth homologous sequences of *Sm*TNFR to construct a probabilistic model using the HMMER tool (v.3.3) [29] following the default parameters. With this model, we interrogated the predicted proteins encoded by 39 platyhelminth genomes, deposited on the Wormbase ParaSite database (release WBPS11, available at https://parasite.wormbase.org/index.html). The BLASTp (v.2.10.0) ([28] tool was also used to screen the Wormbase Parasite database to complete the search for homologous receptors.

To investigate the TNF homologs (ligands) we performed a search using HMMER (with a TNF HMM available at Pfam, PF00229) in the predicted proteins from 39 platyhelminth genomes available in the Wormbase ParaSite database (https://parasite.wormbase.org/index.html). Extensive searches in other deposited platyhelminth genomes did not reveal any other homologous genes. However, searches in RNA-seq experiments from the sequence read archive (SRA, https://www.ncbi.nlm.nih.gov/sra) in other platyhelminth species allowed the detection of reads from organisms that did not have their genomes sequenced, displaying high similarity to the TNF homologs.

### Improvement of platyhelminth gene predictions

It was possible to note that in almost all species only one receptor was detected and that in 10 sequences, their sizes were similar to the one observed for *S. mansoni* (> 450 residues). Eleven sequences were derived from gene models that were probably incomplete due to the small size of the coded protein. Facing this scenario, some gene predictions were manually corrected by the alignment of ESTs, available at GenBank (https://www.ncbi.nlm.nih.gov/genbank/) and reads of RNA-seq available at SRA (https://www.ncbi.nlm.nih.gov/sra). After a manual correction/verification Spaln 2 [30] was used to predict a new gene model of *Sm*TNFR homologs.

### Analysis of conserved domains and secondary structure

A search on the protein domain database (SMART-PRO [31], PFAM [32], Interprot [33] and Prosite [34]) was performed to identify the protein containing conserved domains in platyhelminth TNFR homologs. To improve this search, we selected the cysteine-rich domains (CRD or TNFR domains) identified and used in HMMER to find other less conserved CRD in platyhelminth homologs. JPred 4 [35] (http://www.compbio.dundee.ac.uk/jpred/) was used to identify secondary structures (α-helices) in the primary sequences of TNFR homologs. Sequences of amino acid with a score ≥ 5 were considered as potential α helix regions. Potential DDs were considered when more than three α helix regions were identified.

### Phylogenetic analysis

The alignment of TNFR and TNF domains was performed using MUSCLE [36] in the software MEGA 7.0 [37]. Phylogenetic analysis was performed by Bayesian inference using MrBayes [38]. The phylogenetic trees were visualized with TreeViewX software [39]. The accession numbers of the sequences used in the phylogenetic analysis of TNF receptor homologs are available in Additional file 1: Table S1.

## Results

### TNF receptor homologs are conserved in Platyhelminthes

The increased number of platyhelminth genomes available in the public databases offered us a chance to improve the searching for homologs of TNF receptors and their ligands. We initially used the sequence of *Sm*TNFR as a model to perform a search for similar sequences, through a combined approach with BLAST and HMMER (see Methods). As a result, 31 TNFR were identified in 30 species of platyhelminths: *Echinococcus canadensis*; *E. granulosus*; *E. multilocularis* (2 isoforms); *Hydatigera taeniaeformis*; *Hymenolepis diminuta*; *H. microstoma*; *H. nana*; *Mesocestoides corti*; *Schistocephalus solidus*; *Spirometra erinaceieuropaei* (2 paralogs); *Taenia asiatica*; *T. multiceps*; *T. saginata*; *T. solium*; *Gyrodactylus salaris*; *Macrostomum lignano*; *Clonorchis sinensis*; *Echinostoma caproni*; *Fasciola hepatica*; *Opisthorchis felineus* (2 isoforms); *O. viverrini*; *Schistosoma bovis*; *S. curassoni*; *S. haematobium*; *S. japonicum*; *S. mansoni* (2 new isoforms in addition to the original *Sm*TNFR); *S. margrebowiei*; *S. mattheei*; *S. rodhaini*; and *Trichobilharzia regenti.* It is worth noting that the presence of a TNFR in the free-living platyhelminth, *M. lignano*, indicates the presence of this receptor in an organism that does not interact with vertebrate hosts.

We observed that many of these TNFR sequences were wrongly predicted and/or incomplete, because they did not show all the conserved elements that were expected. We also observed that the markers of quality of the genome assemblies (N50 and number of contigs/scaffolds) were correlated (*P*-value< 0.01) to the number of TNFR domains that were identified (Additional file 2: Figure S1a–c, Additional file 3: Table S1 (columns AY to BA)).

**Fig. 1:**
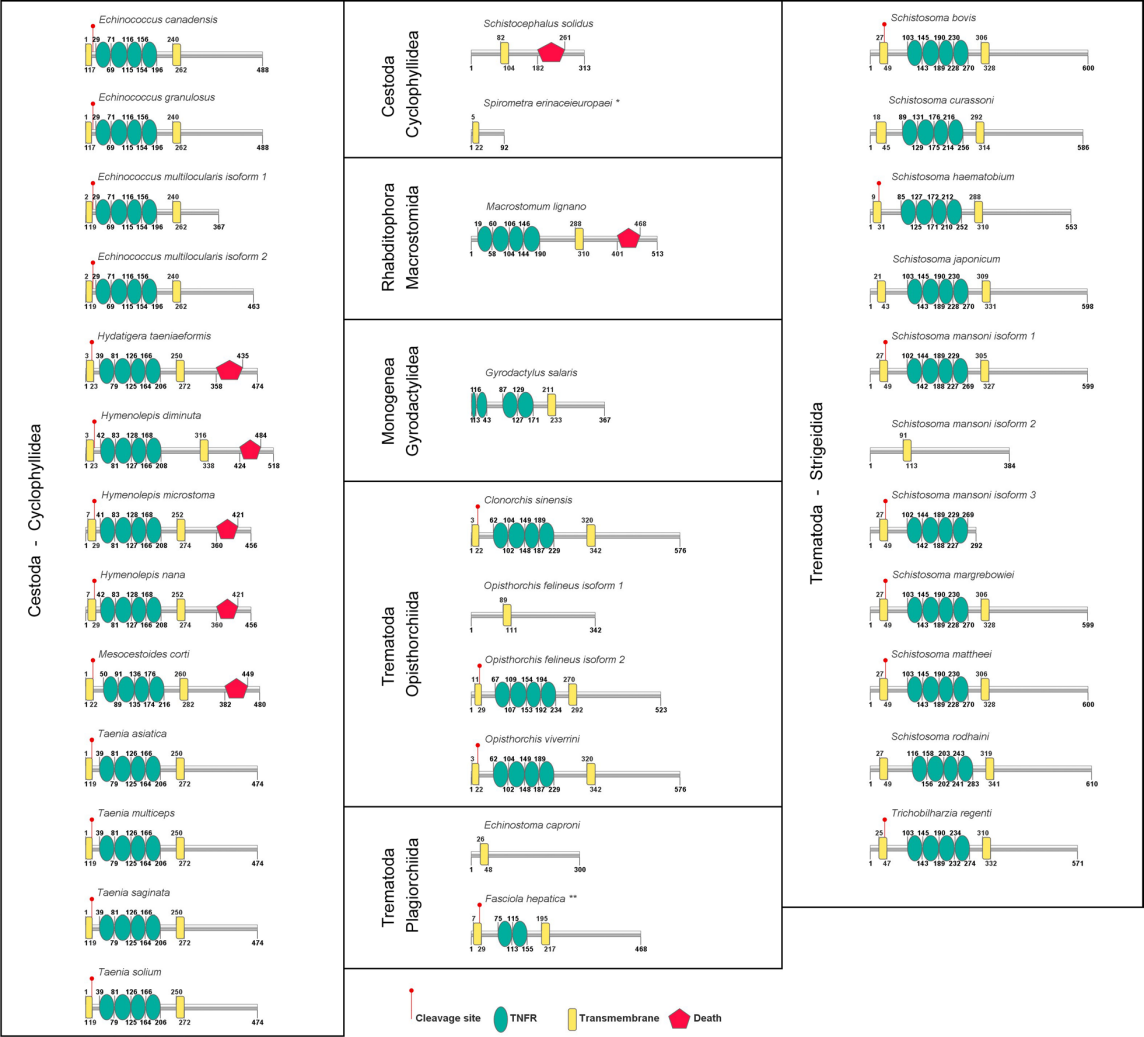
Schematic representation of protein conserved domains in platyhelminth TNFRs. The length of each gray bar is proportional to the sequence length, the number of amino acid residues is indicated on the right side of each bar. The amino acid coordinates of each identified conserved domain are indicated on top (start) and at the bottom (end) of each element. *Key*: green ellipse, TNFR domain; yellow rectangle, transmembrane region; red pentagon, DD; red circle on top of a red line, cleavage site of the signal peptide

To improve the wrong or incomplete gene predictions, we utilized Spaln software to produce new predictions based on the alignments between the genomes and complete ortholog TNFR sequences. In addition, we manually curated some of these genes using information from ESTs or RNA-seq reads, available in the public databases. The final corrected sequences obtained with these approaches are much more reliable and complete when compared to the original sequences. Almost all curated TNFRs display the expected elements: signal peptide (90%); 4 TNFR domains (83%), transmembrane domain (93%) and the intracellular region (96%) (Fig. 1). The corrected sequences of all these homologs are available in Additional file 4: Data S1. Full information about the corrected genes, their genomic contexts and the respective protein conserved elements is provided in Additional file 3: Table S1 (columns J to AU).

It is worth noting that alternatively spliced isoforms that were confirmed by EST and/or RNA-seq reads are present in the following organisms: *E. multilocularis* (2 isoforms); *S. mansoni* (3 isoforms); and *O. felineus* (2 isoforms). Duplication of the TNFR gene was only observed in *S. erinaceieuropaei.* It should be borne in mind that the gene duplication may be an artefact due to the preliminary state of the genome assembly of this organism (see N50 and the number of contigs/scaffolds in Additional file 3: Table S1 (columns AX to BA) and Additional file 2: Figure S1).

TNF receptors comprise a superfamily with more than 25 members that are characterized by CRD, also known as TNFR domains. We aligned the sequences of human TNFR to platyhelminth TNFRs, to compare the similarity and identity among them. The results of 505 alignments are summarized in Additional file 5: Figure S2 and are available in Additional file 6: Table S3. We note that the best score and e-value were observed in the alignments of platyhelminth homologs with human NGFR.

To understand the conservation and phylogenetic context of these TNFRs, we aligned 29 platyhelminth sequences that have 4 TNFR domains (Additional file 7: Figure S3). Eight platyhelminth sequences were not included in this analysis; *F. hepatica* has just 2 TNFR domains, we could not improve the gene model. Other species such as *S. solidus*, *S. erinaceieuropaei* (2 homologs) and *E. caproni* have incomplete gene predictions that are at the extremity of genomic scaffolds; because of this, it was not possible to improve or complete these gene predictions.

It is possible to observe the conservation of the cysteines in the CRD (TNFR domains). The modular architecture of TNFR domains is based on the three-dimensional assembly and the number of disulfide bridges in each module according to Bodmer et al. [40]. The modules of the TNFR domains are classified as A1, A2, B1, B2 and C2 among others. The modular organization of human NGFR is comprised of modules A1 and B2. In a previous study [13], *Sm*TNFR was also defined to be composed of A1 and B2 modules. Based on the alignment of TNFR homologs and on the conservation of cysteines, we assume that the platyhelminth TNFR homologs also have the modular organization A1/B2 similar to *Sm*TNFR and human NGFR. This assumption is supported by the number of disulfide bridges identified in each TNFR domain (see Additional file 3: Table S1 (columns T to AI)). This evidence is also reinforced by the similarities observed in platyhelminth TNFR homologs compared to the human TNFR superfamily (Additional file 5: Figure S2), where the best score and e-value were observed in the alignments of platyhelminth homologs with human NGFR (Additional file 6: Table S3).

**Fig. 2:**
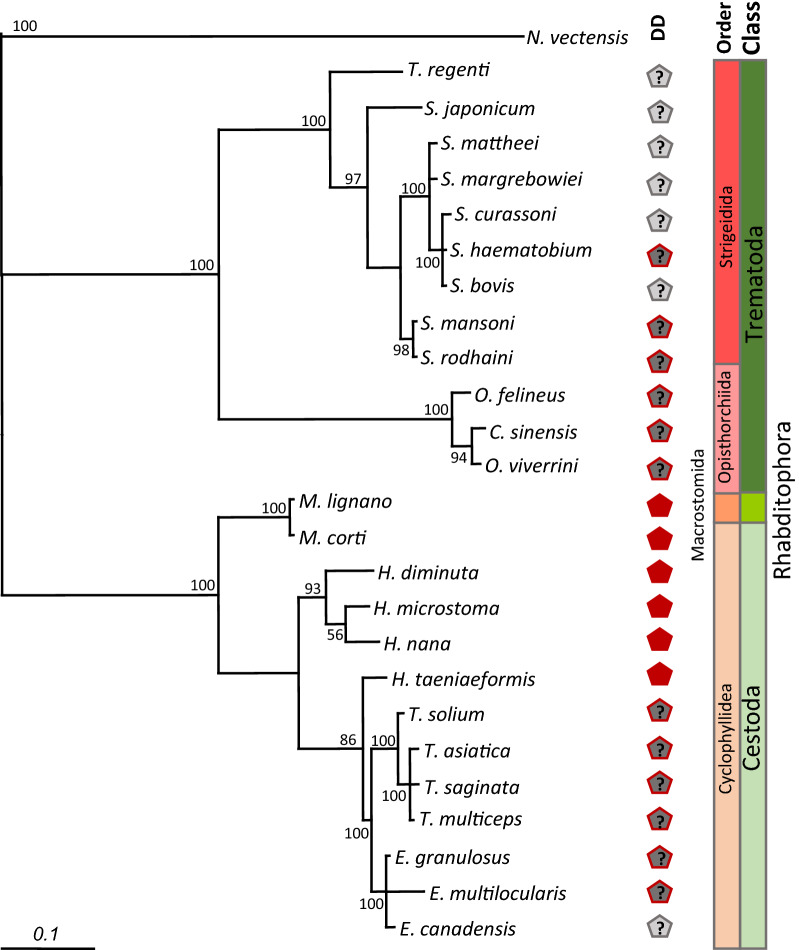
Phylogenetic analysis of conserved TNFR domains of platyhelminth TNFRs. The regions containing four TNFR domains of platyhelminth receptor homologs were used to perform an alignment using MUSCLE. Bayesian inference was used to construct a phylogenetic tree using MrBayes. Clade credibility values are indicated on the left, close to each branch. On the right side, the order and class of each species and the status of DD conservation are represented (red pentagon represents conserved DD, dark gray pentagon with red border represents region with strong evidence of DD; light gray pentagon with dark gray borders represents weak evidence of DD)

Phylogenetic analysis of Platyhelminth TNFR homologs was performed by the method of Bayesian inference using the protein sequences from TNFR domains (Fig. 2).

Extending the analysis to TNFR superfamily members TNFR1, TNFR2 and NGFR (TNFR16) of several species, it was possible to show that all platyhelminth homologs form a branch separated from vertebrate TNFR1 and TNFR2 (Additional file 8: Figure S4). The platyhelminth TNFR branch was grouped with the NGFR branch, but the Bayesian posterior probability values (bpp) joining those two branches was relatively low. Therefore, the evolutionary relationship between platyhelminth and vertebrate TNFRs is still uncertain.

It is interesting to note that seven platyhelminth TNFRs display a conserved DD (*H. taeniaeformis*, *H. diminuta*, *H. microstoma*, *H. nana*, *M. corti*, *S. solidus* and *M. lignano*). This contrasts with *Sm*TNFR in which no DD was previously detected [13]. Analysis of the phylogeny of the TNFR domain shows that the proteins that display DD do not form a monophyletic group (Fig. 2). Therefore, possible interpretations of this scenario are either that multiple events of insertions/deletions of DD occurred during the evolution of TNFR in platyhelminths or that DD in some TNFR are not readily detectable. The latter interpretation is supported by the fact that searches for DD in the CDD databases using a loose e-value threshold (10) allowed detection of DD in most of the TNFR (see Additional file 3: Table S1, column AS). Indeed, it has been previously described that sequence similarity across the DD superfamily is low and analysis of homology between members also relies on structural components, especially the presence of six α-helices that is characteristic of the DD [41].

**Fig. 3:**
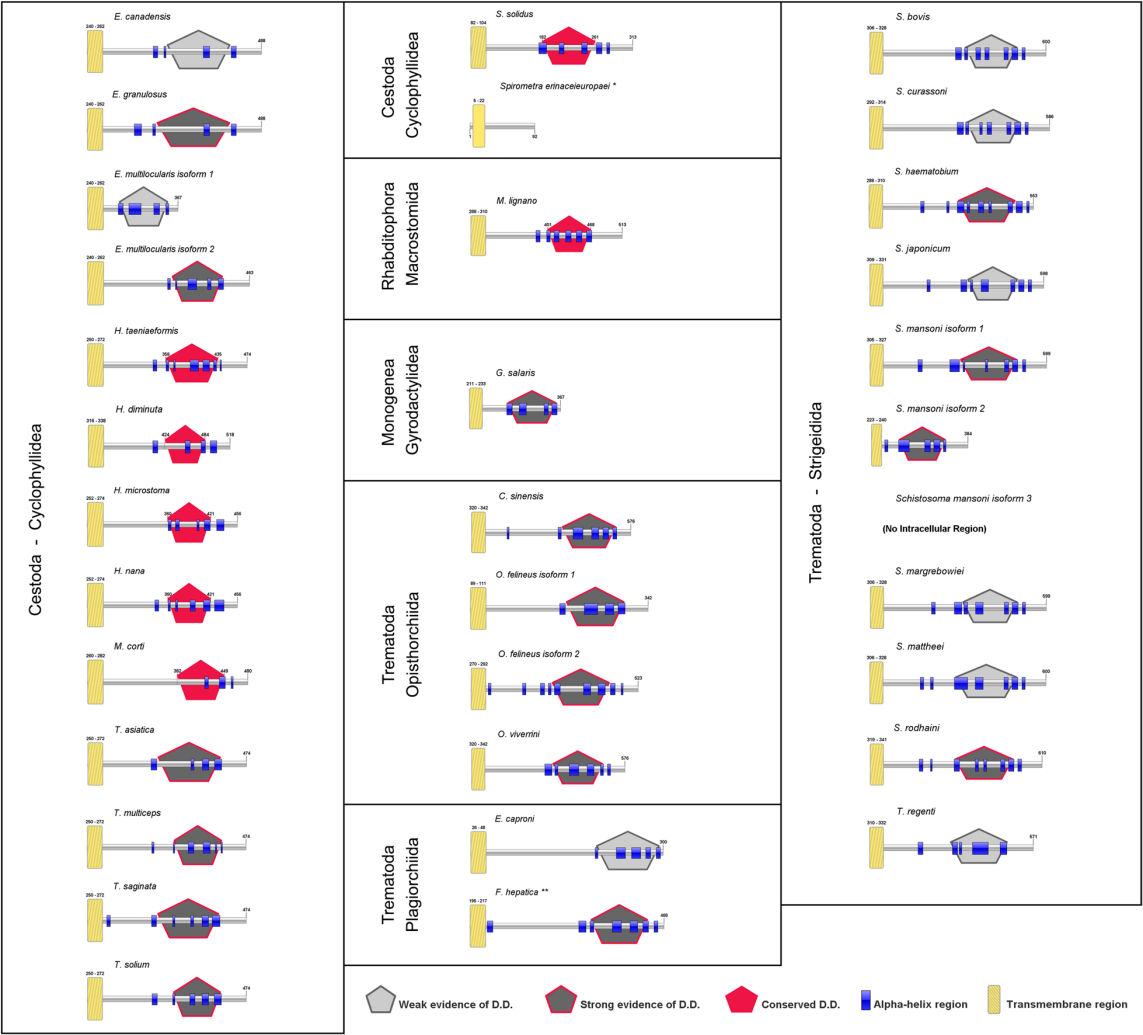
Schematic representation of intracellular elements of platyhelminth TNFRs. *Key*: gray bar, intracellular region of the protein; yellow rectangle, transmembrane region; blue rectangle, α-helix region predicted with JPred; red pentagon, conserved DD; dark gray pentagon with red border, region with strong evidence of DD; light gray pentagon with dark gray borders, region with weak evidence of DD. Numbers indicate amino acid coordinates of the intracellular region and each element

In order to investigate whether TNFR with weak predictions for DD have these conserved structures in the intracellular region we performed a search using JPred4 [35]. The results are shown in Additional file 9: Data S2 and graphically represented in Fig. 3. Almost all platyhelminth TNFR homologs have 5 to 6 α-helices in their intracellular region. This arrangement corresponds to the characteristic number of such structures composing a DD, similarly to those homologs that had a recognizable DD. Moreover, the intracellular regions of TNFR with a conserved DD do not tend to be longer than those from the other TNFRs from platyhelminths (Fig. 3), thus disfavoring the hypothesis of insertion/deletion events.

Based on the results of DD analysis, we classified the platyhelminth TNFR homologs in four categories: (i) conserved DD (recognized by CDD, using the default parameters); (ii) strong evidence of DD (recognized with loose confidence parameters on CDD); (iii) weak evidence of DD (not recognized with loose confidence parameters on CDD, but with more than three α-helices recognized in the intracellular region); and (iv) no evidence of DD (not recognized by CDD and with three or less α-helices in the intracellular region). The classification of DD is summarized in Additional file 3: Table S1 (column AT) and in Fig. 3. We conclude that all complete sequences of platyhelminth TNFR have a predicted and less conserved DD.

### TNF ligand homologs are present in free-living platyhelminths

An important question about the TNFR in platyhelminths regards the presence of an endogenous canonical ligand. Previous searches of the *S. mansoni* genome failed to find any canonical TNF ligand. Therefore, it has been argued that *S. mansoni* TNFR either binds a ligand from the human host or to a non-canonical ligand. The first hypothesis is supported by the fact that *in vitro* incubation with human TNF-α induces changes in the parasite gene expression [13]. However, in our previous analysis [13] we noted the presence of TNFR in free-living platyhelminths, thus indicating that response to host ligand level cannot be the sole function of such receptors.

To revisit this question and obtain more definitive answers, we performed a comprehensive search for TNF ligands using HMMER program using the TNF HMM (PFAM, PF00229) on the predicted proteins from platyhelminth genomes available on the Wormbase Parasite website (https://parasite.wormbase.org/index.html).

As a result, four proteins containing a prediction of a TNF domain with high confidence (e-value < 10^−10^) were found in the genome of *M. lignano*, a free-living platyhelminth. Extensive searches using these proteins as BLASTp queries against other deposited platyhelminth genomes did not produce significant hits. However, TBLASTn searches of these proteins against other platyhelminth RNA-seq sequences from SRA (https://www.ncbi.nlm.nih.gov/sra) allowed the detection and reconstruction of 16 TNF ligands in four species of the Class Rhabditophora: *M. lignano* (6 additional sequences); *Macrostomum tuba* (4 sequences); *Microdalyellia ruebushi* (2 sequences); and *Microdalyellia fusca* (1 sequence) (Additional file 10: Data S3).

**Fig. 4:**
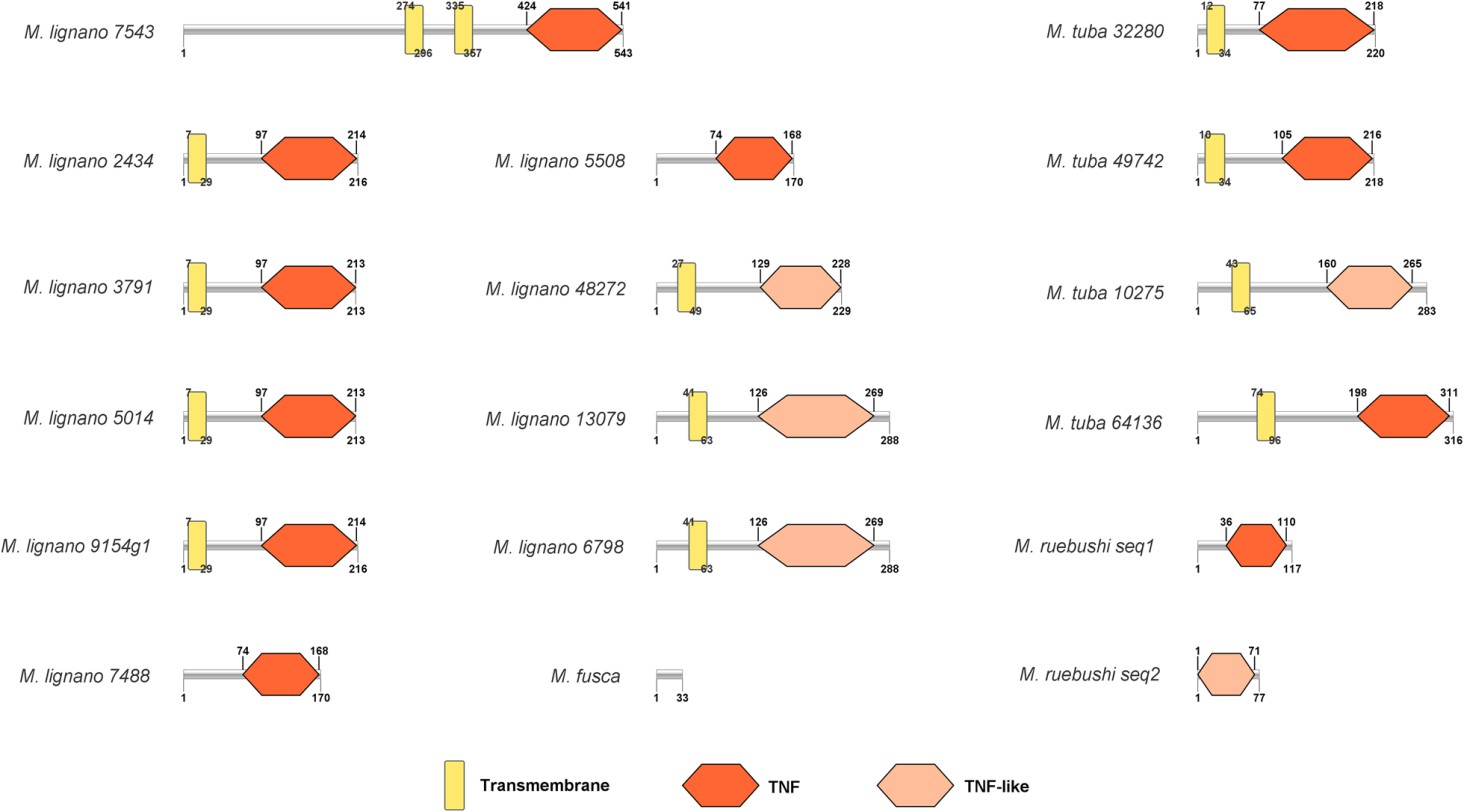
Schematic representation of protein conserved domains in platyhelminth TNF ligands. Each gray bar length is proportionally adjusted based on the total number of amino acids residues, the number of amino acid residues is indicated on the right side of each bar. The coordinates of each identified conserved domain are indicated on top (start) and at the bottom (end) of each element. *Key*: yellow rectangle, transmembrane region; orange hexagon, TNF conserved domain; light orange hexagon, TNF-like domain (belonging to the TNF family)

In almost all of these sequences, a TNF domain was identified and, in several of them, we were detected transmembrane helices (Fig. 4, Additional file 11: Table S4). The presence of transmembrane helices suggests that platyheminth TNF homologs must be subjected to the process of shedding to produce soluble factors, as commonly described for other proteins of this class.

It is also noteworthy that the alignment of these proteins with BLASTp program against the NR database resulted in seven of them having as first hit TNF ligands from fishes, while the others produced no significant hits outside platyhelminths. This suggests that these TNF ligands are not closely related to other proteins from the TNF family described in invertebrates. Alignment of these platyhelminth TNF homologs with human TNF ligand family members showed an identity within the range of 20–30%, with several human TNF proteins (Additional file 12: Figure S5 and Additional file 13: Table S5), including TNF-α.

**Fig. 5:**
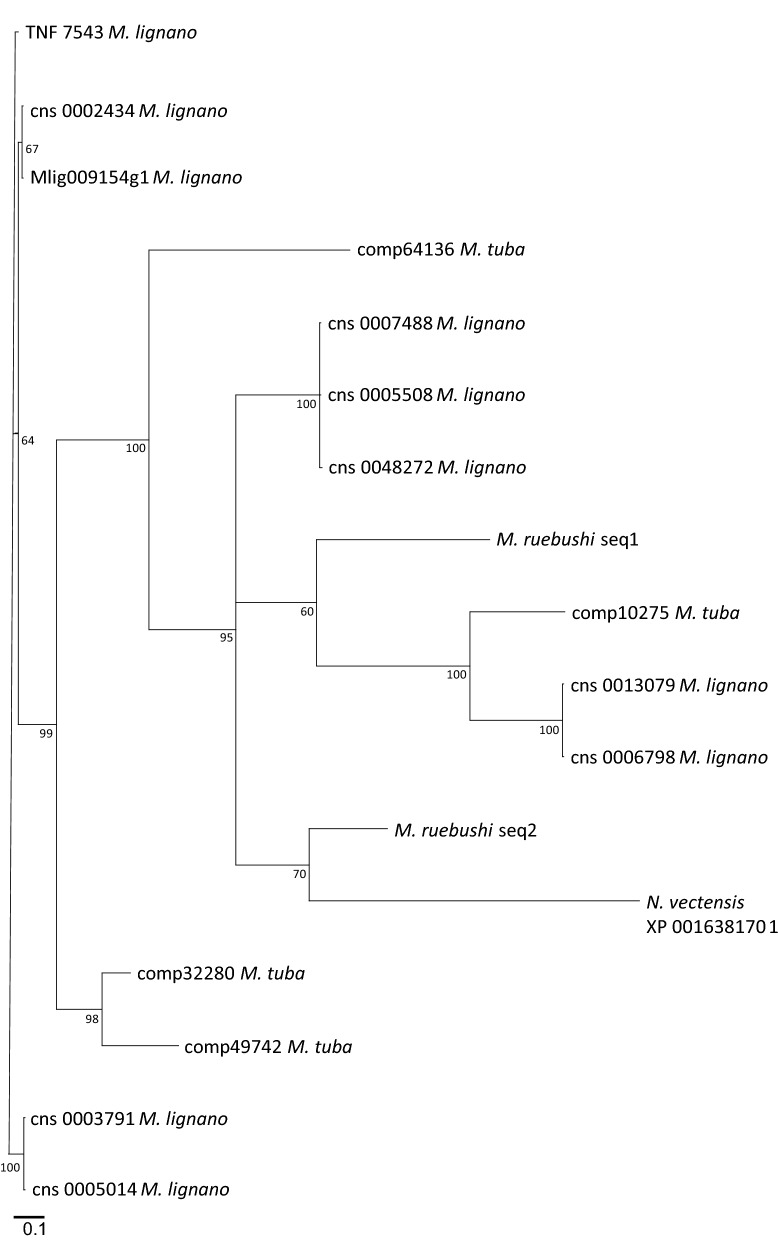
Phylogenetic analysis of conserved TNF domains of Platyhelminth TNFs. The region containing TNF domains of platyhelminth homologs was used to perform an alignment using MUSCLE. Bayesian inference was used to construct a phylogenetic tree using MrBayes. Clade credibility values are indicated at the nodes

Platyhelminth TNF-α conserved domains were aligned (Additional file 14: Figure S6) to perform a phylogenetic analysis by using Bayesian inference (Fig. 5). This result suggests that multiple events of gene duplication of the TNF ligand occurred in the Rhabditophora. Although most of these duplication events appear to be species-specific, the topology suggests that few ancestral events occurred. Most of species-specific duplications results in pairs of proteins with very little distance and interpretation of duplication must be cautious, since the different sequences can be the result of polymorphisms rather than genomic duplication.

## Discussion

TNFR signaling may represent an important system to understand host-parasite crosstalk in Platyhelminthes. Previous descriptions of the influence of human TNF-α in *S. mansoni* gene expression, egg-laying and protein metabolism highlighted the possible responses of the parasite to the immune signaling of the host [7–13]. The fact that *S. mansoni* displays a TNFR, but apparently lacks an endogenous canonical ligand, provided further evidence that this signaling system might have been co-opted to respond to TNF-α levels from the human host. However, similarities between *Sm*TNFR and NGFR raised the possibility that the *Schistosoma* receptor might be binding to a non-canonical ligand as observed for the latter [13].

Considering this scenario, a more comprehensive analysis of TNFRs in platyhelminths was warranted. Our analysis detected TNFRs in 30 out of 39 analyzed platyhelminth genomes, assuming that the sequence conservation can represent conservation of the biological function. This finding suggests that TNF signaling may be important for platyhelminth biology. The placement of the platyhelminth TNFR branch in relation to vertebrate branches remains unclear. It should be noted, however, that the lack of diversification of platyhelminth TNFR might indicate a basal divergence before the diversification observed in TNFR of vertebrates.

We found canonical TNF ligands in some free-living platyhelminth species. The relatively high level of conservation between the different TNFRs of platyhelminths is suggestive that all of these receptors must be able to bind canonical TNF ligands. The failure to find a gene for a canonical TNF ligand in all parasitic species suggests that host-parasite crosstalk might be a common feature. It is important to note that we did not find TNF ligands for all free-living platyhelminths, but this could reflect the incomplete status of the genome sequencing of these organisms. Further genomic sequencing to obtain more reliable assembly is necessary to evaluate whether the presence of a TNF ligand is a common trait in all free-living platyhelminths or just from some evolutionary branches.

**Fig. 6:**
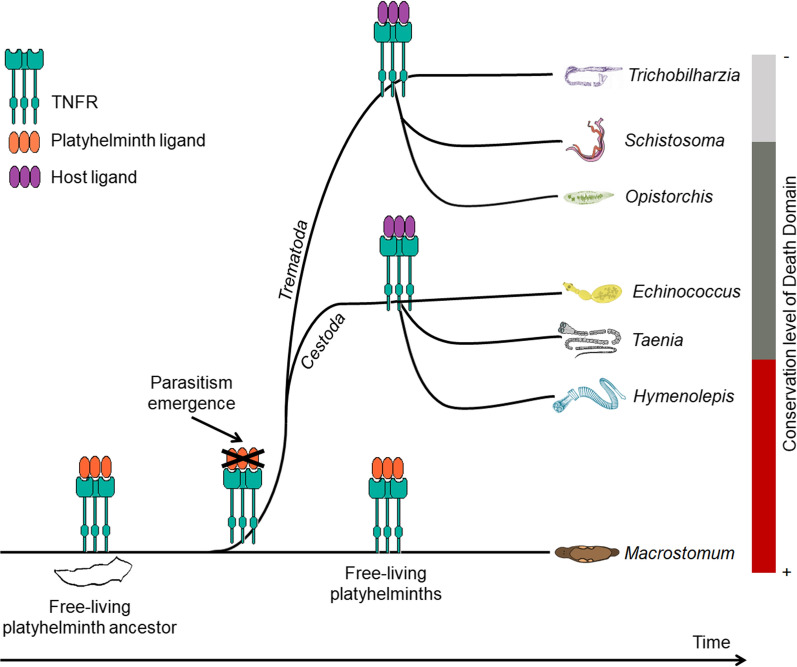
Schematic representation of proposed evolution of TNF signaling in platyhelminths. Here, we hypothesize that an ancestor free-living platyhelminth displayed genes that code for TNF receptor (represented by green sticks) and TNF ligand (represented by orange spheres). The event of parasitism implicated in the substitution of the platyhelminth TNF ligand by the host TNF causing the loss of the coding gene for the former. Probably the loss of the TNF ligand is ancestral, occurring at the base of the monophyletic branch of parasitic platyhelminths. X axis represents a putative temporal line and Y axis in the right represents the conservation level of DD (from the most conserved at the bottom to the lowest conserved at the top)

That scenario given, it is possible to hypothesize that an ancestral free-living platyhelminth displayed both a TNFR and its ligand. Throughout evolution, the parasitic platyhelminth branch acquired the ability to interact with the host TNF ligand and lost its endogenous ligand (Fig. 6). Such a scenario is compatible with the description that all obligatory parasitic species are monophyletic [42] and would indicate that switch from an endogenous to an exogenous ligand occurred early in the adaptation of platyhelminths to a parasitic lifestyle (Fig. 6).

We also observed for the first-time DD in the intracellular region of TNFR from a group of platyhelminths. Our analysis suggests that the presence of a DD might be a widespread feature in platyhelminth TNFR. However, solely TNFR from free-living organisms and those of basal cestodes showed domains with high conservation. The other platyhelminths display poorly conserved domains and it remains to be confirmed that such domains are able to induce apoptotic processes. One possible explanation for the predominance of poorly conserved domains in parasitic platyhelminths is that after change from an endogenous to an exogenous ligand evolutionary pressures that allow the occurrence of mutations in the DD to a more adequate response for a potential new function.

## Conclusions

Considering the widespread presence of TNFR in Platyhelminthes and the additional evidence that these receptors were subjected to changes to perform roles related to the host-parasite relationship, a comprehensive study to verify if other platyhelminths respond and their receptors interact with TNF-α is needed. Moreover, *in vivo* experiments to verify if such signaling induces apoptotic process are also desirable to understand the nature of this molecular crosstalk. The understanding of the molecular mechanism involved in evolutionary adaptation of parasites to the host signals is essential to enhance our knowledge that may help develop new approaches to combat the parasitic diseases.

## Supplementary information

**Additional file 1: Table S1.** Accession numbers of all sequences used in the phylogenetic analysis.

**Additional file 2: Figure S1.** Analysis of the quality parameters of the platyhelminth genome assemblies of all genomes used in this study, compared to the number of conserved TNFR domains that were identified in the original homolog gene predictions, according to each species.

**Additional file 3: Table S2.** Detailed information of platyhelminth TNFR homologs.

**Additional file 4: Data S1.** All manually corrected FASTA sequences of platyhelminth TNFR homologs.

**Additional file 5: Figure S2.** Matrices of BLAST results obtained by the alignment of human TNF receptors and platyhelminth TNF receptors (see detailed description in the figure).

**Additional file 6: Table S3.** BLASTp results of alignments between human TNFR and platyhelminth TNFR homologs.

**Additional file 7: Figure S3.** Alignment of TNFR domains of platyhelminth homologs (see detailed description in the figure).

**Additional file 8: Figure S4.** Phylogenetic analysis of conserved TNFR domains of platyhelminths and TNFR homologs of other species (human and mammals). See detailed description in the figure.

**Additional file 9: Data S2.** JPred analysis documentation of platyhelminth TNFR sequences (see details in the file).

**Additional file 10: Data S3.** All manually corrected FASTA sequences of platyhelminth TNF homologs.

**Additional file 11: Table S4.** Detailed information of platyhelminth TNF homologs.

**Additional file 12: Figure S5.** Matrices of BLAST results obtained by the alignment of human TNF ligands and platyhelminths TNF ligands (see detailed description in the figure).

**Additional file 13: Table S5.** BLASTp results of alignments between human TNF and platyhelminth TNF homologs.

**Additional file 14: Figure S6.** Alignment of TNF domain of platyhelminth homologs (see detailed description in the figure).

## Data Availability

The datasets supporting the findings of this article are included within the article and its additional files.

## Abbreviations

BLAST: Basic Local Alignment Search Tool
CDD: Conserved Domain Database
CRD: cysteine-rich domain
DD: death domain
EST: expressed sequence tag
HMM: hidden Markov model
NGF: nerve growth factor
NGFR: NGF receptor
SCID: severe combined immunodeficient
*Sm*TNFR: *S. mansoni* TNFR
SRA: Sequence Read Archive
TNF: tumor necrosis factor
TNFR: TNF receptor
