# Supplementary material for: The evolution of TNF signaling in platyhelminths suggests the cooptation of TNF receptor in the host-parasite interplay
Source: Parasit Vectors. 2020 Sep 25;13:491. doi: 10.1186/s13071-020-04370-1 (PMC7519573; doi:10.1186/s13071-020-04370-1)
Supplement: Supplementary file 7 — Additional file 7: Figure S3. Alignment of TNFR domains of platyhelminth homologs (see detailed description in the figure). [file 13071_2020_4370_MOESM7_ESM.pdf]

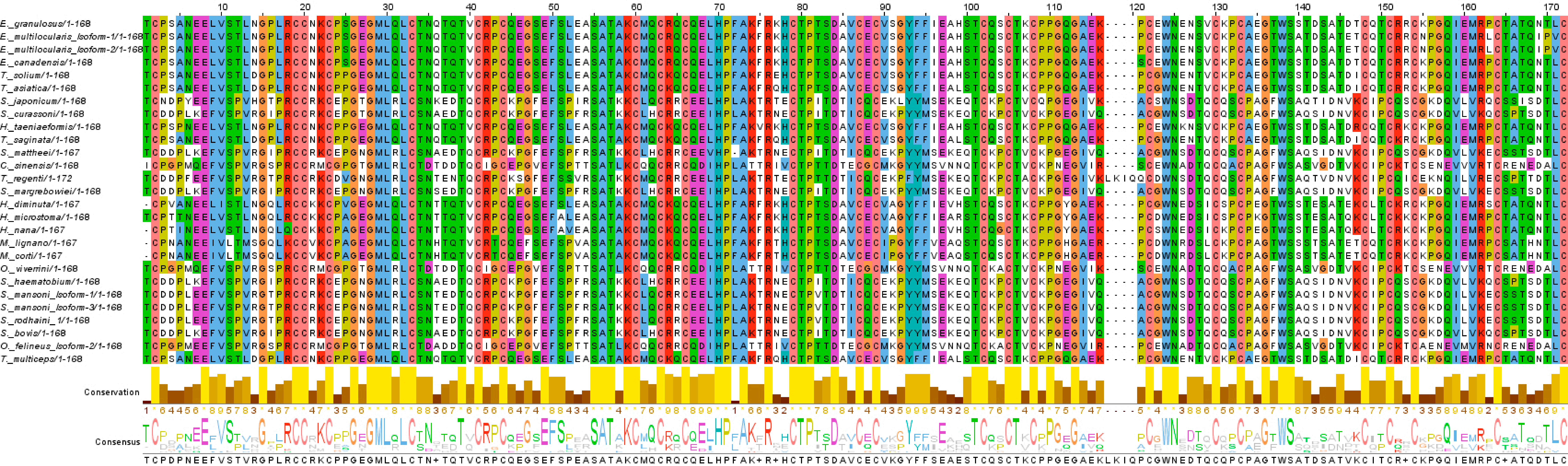

Additional file 7: Figure S3. Alignment of TNFR domains of platyhelminth homologs. Alignment of 25 homolog sequences with four TNFR domains (around 170 amino acid residues) was performed by MUSCLE algorithm (MEGA 7.0 software). Amino acid residues were colored according to CLUSTAL pattern, at the bottom, the conservation level of each residues and consensus sequence are represented, as indicated.
