# Supplementary material for: The evolution of TNF signaling in platyhelminths suggests the cooptation of TNF receptor in the host-parasite interplay
Source: Parasit Vectors. 2020 Sep 25;13:491. doi: 10.1186/s13071-020-04370-1 (PMC7519573; doi:10.1186/s13071-020-04370-1)
Supplement: Supplementary file 8 — Additional file 8: Figure S4. Phylogenetic analysis of conserved TNFR domains of platyhelminths and TNFR homologs of other species (human and mammals). See detailed description in the figure. [file 13071_2020_4370_MOESM8_ESM.pdf]

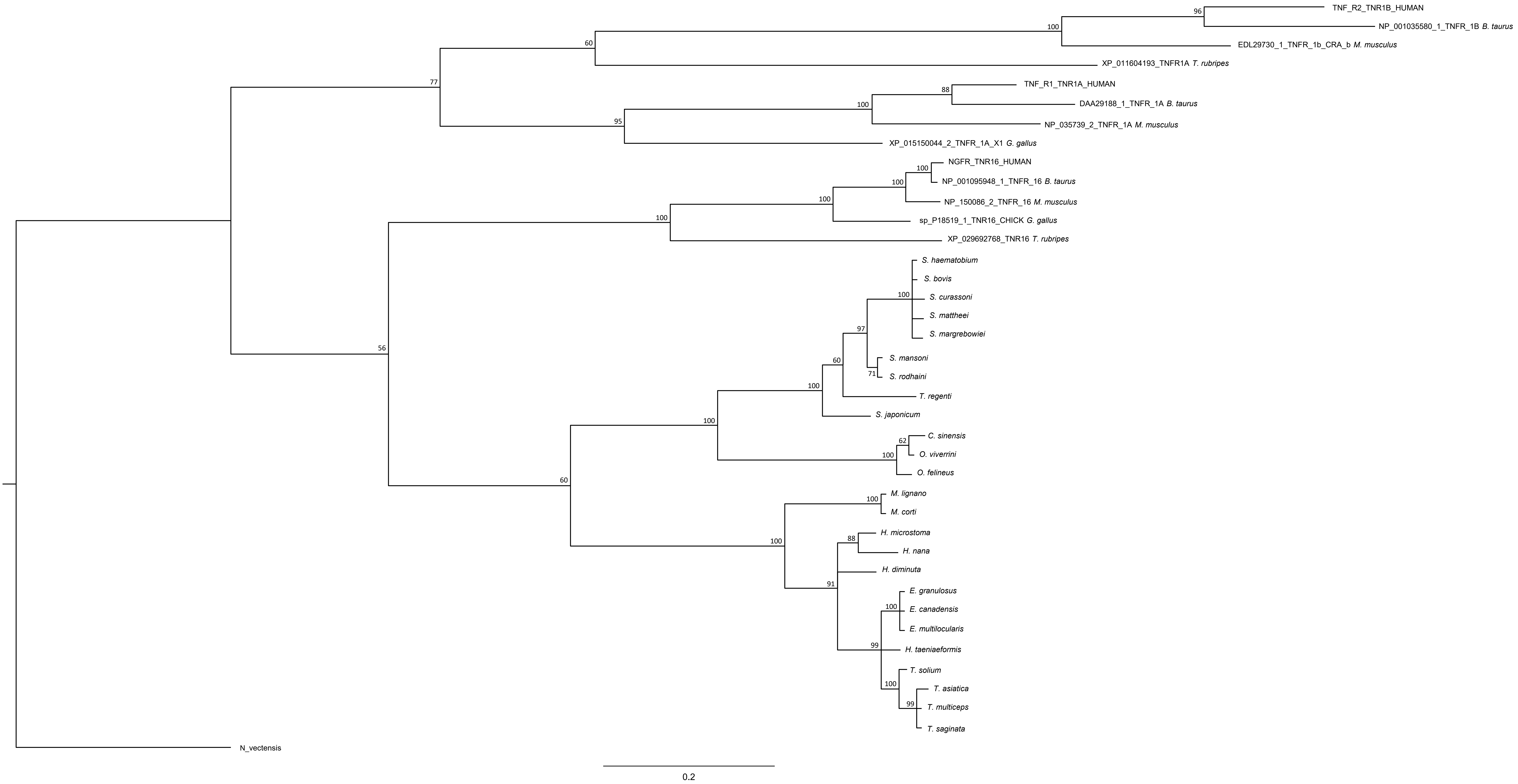

Additional file 8: Figure S4. Phylogenetic analysis of conserved TNFR domains of platyhelminths and TNFR homologs of other species (human and mammals). The regions containing TNFR domains of platyhelminths and other species homologs were used to perform an alignment using the MUSCLE tool. Bayesian inference was used to construct a phylogenetic tree using the MrBayes tool. Clade credibility values are indicated on the left, close to each branch.
