# Supplementary material for: The evolution of TNF signaling in platyhelminths suggests the cooptation of TNF receptor in the host-parasite interplay
Source: Parasit Vectors. 2020 Sep 25;13:491. doi: 10.1186/s13071-020-04370-1 (PMC7519573; doi:10.1186/s13071-020-04370-1)
Supplement: Supplementary file 9 — Additional file 9: Data S2. JPred analysis documentation of platyhelminth TNFR sequences (see details in the file). [file 13071_2020_4370_MOESM9_ESM.docx]

**Additional file 9: Data S2.** JPred analysis documentation of platyhelminth TNFR sequences. In yellow, the transmembrane regions are highlighted. In red, the conserved DD that were recognized by Pfam, CDD and Prosite are highlighted.

> Schistosoma mansoni ISOFORM 1 - ACS92719.1 (Smp_332480.1)

Jnet Rel : 93103111222688622001167873132136777766313336643346677777777777777777777777777676777764102046777666654567777642256777765356777763461367777776321346777667667777763213467776666555566777777777777777777777777777777776532315677776654567765133667776777533357773188303778774143167777667777777777777777777777776777775213134677766677777776653445667777777777776545677654566777677776301167777766278999987116788761120120367777665340223530111101358860799999999999999863367178887430665443455312234332421788764037999931126661671367777777763220389999985318737899999987027841256655541133316788775222101241256777742278 : Jnet Rel
 : 1---------11--------21--------31--------41--------51--------61--------71--------81--------91--------101-------111-------121-------131-------141-------151-------161-------171-------181-------191-------201-------211-------221-------231-------241-------251-------261-------271-------281-------291-------301-------311-------321-------331-------341-------351-------361-------371-------381-------391-------401-------411-------421-------431-------441-------451-------461-------471-------481-------491-------501-------511-------521-------531-------541-------551-------561-------571-------581-------591------ :

OrigSeq : MHRIYQFVHSFDAHTKHTITCMHNSKVFIELLCLTIVWNSVIAGPLIFQSETLEGKTYPAVNTSQMNNSNNTISSNENNETVATTVGTIEGTGDIVEIQTETCDDPLEEFVSPVRGTPRCCRKCEPGNGMLRLCSNTEDTQCRPCKPGFEFSPFRSATKKCLQCRRCEELHPLAKTRNECTPVTDTICQCEKPYYMSEKEQTCKPCTVCKPGEGIVQACGWNSDTQCQSCPAGFWSAQSIDNVKCIPCQSCGKDQILVKECSSTSDTLCCPVNNPNCTHELSMYFDYSAYDQESDISDNNNKSNQMLPIYCSIMGLIIISLLCYVVYKLWRQREASKNAKLADSYNSNKTDLLDRTSCLDNNHLQHRRISSGFVNNSAHLPNHSPQSANELNNIADSTPDNDINNQLQFNDIIYGHEKAPLLGKLDHSNSSFNNFEQPITVIPMNILGVICYRLSQHGWQELASMLDLETSKFDQLPSEITSDLLSATMEAQTTAESHLKQCNQDNSNTIQPITNNNNNNNKNNLTMTVSMFQYMCLQNTVNLGQLMNSLQKLNSPDLVALIQQQIGIIKSKKTINQSNEEYKTKTKSIKSKENFQIEN : OrigSeq

Jnet : ---HHHH-----------E-------EEE----------------------------------------------------------EE---------------------------------------EEE---------------------------------------------------------------------------------------------------------------------------EEEE--------EEEE---------------------------------------EEE------------------------------------------------------------EE----------HHHHHHHHH--------HHH--------------------HHHHHH------HHHHHHHHHHHHHHHHHHH---HHHHHHHH------------HHHHHHHHH--------HHHHHHHH------EEE------------EEHHHHHHHHHHH---HHHHHHHHHHHH----HHHHHHHHHHEEEE---------HHHH---------------- : Jnet

jhmm : -HHHHH---------------------------------------------------------------------------------EE---------------------------------------EE-----------------------------------------------------------------------------------------------------------------------------EE---------EEEE-----------------------------------------------------------------------------------------------------EEEE---------HHHHHHHH--------HHH---------------------------------HHHHHHHHHHHHHHHHHHH---HHHHHHHHH-----------HHHHHHHHHH-------HHHHHH--------EE-------------EEEEHHHHHHHHHH--HHHHHHHHHHH-----HHHHHHHHH-EEEE----------------------------- : jhmm

jpssm : ---HHHHHEE------EEEE------EEEE----------EE--------------------------------------------EEE---------------------------------------EEE----------EEE-----------------EEE-------------------------------------------------EEE------------------E-------------------EEEEE-------EEE---------------------------------------EEEEE-----------------------------------------------------------------------HHHHHHHHH--------HHHHH-----------H------HHHHHHHH----HHHHHHHHHHHHHHHHHHH----HHHHHH------------HHHHHHHH---------HHHHHHHHHH----EEEE------------HHHHHHHHHHHH-----HHHHHHHHHHH-----HHHHHHHHHEEEE---------HHHHHHH---------E--- : jpssm

Lupas 14 : ----------------------------------------------------------------------------------------------------------------------------------------------------------------------------------------------------------------------------------------------------------------------------------------------------------------------------------------------------------------------------------------------------------------------------------------------------------------------------------------------------------------------------------------------------------------------------------------------------------------------- : Lupas 14

Lupas 21 : ----------------------------------------------------------------------------------------------------------------------------------------------------------------------------------------------------------------------------------------------------------------------------------------------------------------------------------------------------------------------------------------------------------------------------------------------------------------------------------------------------------------------------------------------------------------------------------------------------------------------- : Lupas 21

Lupas 28 : ----------------------------------------------------------------------------------------------------------------------------------------------------------------------------------------------------------------------------------------------------------------------------------------------------------------------------------------------------------------------------------------------------------------------------------------------------------------------------------------------------------------------------------------------------------------------------------------------------------------------- : Lupas 28

Jnet_25 : ---BB-BBBBB---B-BBBBB-B-B-BBB-B-BB-BBB-BBBBBBBBBBB--B-BBBBBBBBBB-BBBBBBBBBBB-BB-BB-BB-BBB-B--BBBBB-B--B--B---BBB-------BB--B--B-BBB--B-----B-BBBB-----BB-B------B-BB--B-B-B-BBBB-B-B-BB-BBBBBBB-BBBBB--B--BBBB--B--B-BBB-BB--B---BB--B--BBBBB--B----B--B--B-----BB--B---BBBBBB-B----B--B--BBBBBBB----B--B-------BBBB-BBBBBBB-BBBB----BB-----------B----------B--B----B---BB---------BB-----B-------B--BB--B----B--BB--B-B---B--B--BBBBBB---BB--B-----BBBBBBB-BBBBBBBBB----B--BB-BB-B-B--B--BB--B---BB-BB--B---B---B--B------BB--B-----------B-BBBBBB-BBB---BB-B--BB--B--B----BBBBB---B-BB-----B------B---B--B-----B-B-- : Jnet_25

Jnet_5 : -------B--B------BB-B-----BB-------BB--BBBBB-BBB---------------B--------B---------------B-------------B-----------------B--B-----------------B--------------------------------------------------------------------------------------------B---------B--B------------------B-B----------------------------------------B-BB---------------------------------------------------------------------------------------------------------B-B-----------------B-BBB--BB--BB--B-------BB--B-----------------------------------B----------------------B---B-BB---B------B--B---B-------BB-BB------------------------------------- : Jnet_5

Jnet_0 : ----------------------------------------B------------------------------------------------------------------------------------------------------------------------------------------------------------------------------------------------------------------------------------------------------------------------------------------------------------------------------------------------------------------------------------------------------------------------BB---------------------------------------------------------------------------------------------------------------------------------------------------- : Jnet_0

Jnet Rel : 93103111222688622001167873132136777766313336643346677777777777777777777777777676777764102046777666654567777642256777765356777763461367777776321346777667667777763213467776666555566777777777777777777777777777777776532315677776654567765133667776777533357773188303778774143167777667777777777777777777777776777775213134677766677777776653445667777777777776545677654566777677776301167777766278999987116788761120120367777665340223530111101358860799999999999999863367178887430665443455312234332421788764037999931126661671367777777763220389999985318737899999987027841256655541133316788775222101241256777742278 : Jnet Rel

: 1---------11--------21--------31--------41--------51--------61--------71--------81--------91--------101-------111-------121-------131-------141-------151-------161-------171-------181-------191-------201-------211-------221-------231-------241-------251-------261-------271-------281-------291-------301-------311-------321-------331-------341-------351-------361-------371-------381-------391-------401-------411-------421-------431-------441-------451-------461-------471-------481-------491-------501-------511-------521-------531-------541-------551-------561-------571-------581-------591------ :

> Schistosoma mansoni ISOFORM 2 - Smp_332480.2

Jnet Rel : 898877773464267777761464267777767764211011111056776651126663025631133125775566777777777777611378998747999999899999999873247760302457776640123467776653331267777776420477775212120236777777777664224212111664330334677776565322112232214154345665214378888603678776666543220658999999999999887642678873100026777777762236889999985068707899999987527843267663013112316787775201101231256777744278 : Jnet Rel
 : 1---------11--------21--------31--------41--------51--------61--------71--------81--------91--------101-------111-------121-------131-------141-------151-------161-------171-------181-------191-------201-------211-------221-------231-------241-------251-------261-------271-------281-------291-------301-------311-------321-------331-------341-------351-------361-------371-------381- :

OrigSeq : MAPLPVTNNVIVSDTTIRPSELYVKNSKEYDKFDENEMKVLNWQTLLPRSSSDQLENRTYPMHIQTVFRIDDYSAYDQESDISDNNNKSNQMLPIYCSIMGLIIISLLCYVVYKLWRQREASKNAKLADSYNSNKTDLLDRTSCLDNNHLQHRRISSGFVNNSAHLPNHSPQSANELNNIADSTPDNDINNQLQFNDIIYGHEKAPLLGKLDHSNSSFNNFEQPITVIPMNILGVICYRLSQHGWQELASMLDLETSKFDQLPSEITSDLLSATMEAQTTAESHLKQCNQDNSNTIQPITNNNNNNKNNLTMTVSMFQYMCLQNTVNLGQLMNSLQKLNSPDLVALIQQQIGIIKSKKTINQSNEEYKTKTKSIKSKENFQIEN : OrigSeq

Jnet : ---------EEE---------EEE-------------HHHHH-HH----------------HHHHHEEEEE----------------------HHHHHHHHHHHHHHHHHHHHHHHHHHHH---------------------------------------------------HHHHH-------------------HHHH-----------------------EEEEE---HHHHHHHHHHHHHHHHHHHH----------------HHHHHHHHHHHHHHHHHHHH--------HHH----------HHHHHHHHHHHHH-----HHHHHHHHHHH-----HHHHHHH---EEE----------HHH---------------- : Jnet

jhmm : ---------EEE---------EEE-------------HHHHHHHHH-------HHH------HHEEEEEEE----------------------HHHHHHHHHHHHHHHHHHHHHHHHHHHH-------------------------------EE--------------------------------------HH-HHHHHH---------------------EEEEEE----HHHHHHHHHHHHHHHHHH-----------------HHHHHHHHHHHHHHHHHHHH------HHHHHH---------HHHHHHHHHHHHH----HHHHHHHHHHHH-----HHHHH-----EEE----------------------------- : jhmm

jpssm : ---------EEE---------EEE--------------EEE--EE----------------HHHHHHHHE----------------------HHHHHHHHHHHHHHHHHHHHHHHHHHHH------E---------------------------------------------HHHHHH-------------------EEE-------E----------------HHHHH--HHHHHHHHHHH--HHHHHHH------------HHHHHHHHHHHHHHHHHHHHHHHH-------EEEE-----------HHHHHHHHHHHH-----HHHHHHHHHHH-----HHHHHHHHH-EEE---------HHHHHHH---------E--- : jpssm

Lupas 14 : ------------------------------------------------------------------------------------------------------------------------------------------------------------------------------------------------------------------------------------------------------------------------------------------------------------------------------------------------------------------------------------------------ : Lupas 14

Lupas 21 : ------------------------------------------------------------------------------------------------------------------------------------------------------------------------------------------------------------------------------------------------------------------------------------------------------------------------------------------------------------------------------------------------ : Lupas 21

Lupas 28 : ------------------------------------------------------------------------------------------------------------------------------------------------------------------------------------------------------------------------------------------------------------------------------------------------------------------------------------------------------------------------------------------------ : Lupas 28

Jnet_25 : ---------BBBB---B----BBB-----B--B----B-BB-B-BBB-------B----BB-BB-BBB-B--BB-B-----B---------BBBBBBBBBBBBBBBBBBBBBB-BB--------B-BB-BB-B----BB--B-BB----B----B--BBB--BB-BB---------B---B-------------B--BBB-B---BBBB-B--B---B--B---BB-B-B-BBBBBBB-B---BB--BB--B-B-B--B--BB--B---BB-BB--B-------B--B------BB--BB---------B-BBB-BBB-BB---BB-B--BB--B--B----BBBBB---B--B-----B------B---B--B-----B-B-- : Jnet_25

Jnet_5 : ---------------------------------------------------------------B--BB-B----------------------B-B-B-------B------B--B-----------B-------------------------------------------------B--------------------B--------BB------------B---B--B---BBBBBB--B-------B---B-------------------------------------------B-------------B---B-BB--BB------B--BB--B-------BB-BB------------------------------------- : Jnet_5

Jnet_0 : -----------------------------------------------------------------------------------------------------------------------------------------------------------------------------------------------------------------------------------------B-BB------------------------------------------------------------------------------------------B--B---------------B------------------------------------- : Jnet_0

Jnet Rel : 898877773464267777761464267777767764211011111056776651126663025631133125775566777777777777611378998747999999899999999873247760302457776640123467776653331267777776420477775212120236777777777664224212111664330334677776565322112232214154345665214378888603678776666543220658999999999999887642678873100026777777762236889999985068707899999987527843267663013112316787775201101231256777744278 : Jnet Rel

: 1---------11--------21--------31--------41--------51--------61--------71--------81--------91--------101-------111-------121-------131-------141-------151-------161-------171-------181-------191-------201-------211-------221-------231-------241-------251-------261-------271-------281-------291-------301-------311-------321-------331-------341-------351-------361-------371-------381- :

> Schistosoma mansoni ISOFORM 3 - Smp_332480.3

Jnet Rel : 9324333311327851233047775111133447743320331367764101356414677776777777777777767777765211234677763010012688772461367776535677776346514677777512113677652246777776411576532111112230367777777777777777765332221577777751331467777765456777641466777776511025777318881377877246436777766776777775323427 : Jnet Rel
 : 1---------11--------21--------31--------41--------51--------61--------71--------81--------91--------101-------111-------121-------131-------141-------151-------161-------171-------181-------191-------201-------211-------221-------231-------241-------251-------261-------271-------281-------29 :

OrigSeq : MHRIYQFVHSFDAHTKHTITCMHNSKVFIELLCLTIVWNSVIAGPLIFQSETLEGKTYPAVNTSQMNNSNNTISSNENNETVATTVGTIEGTGDIVEIQTETCDDPLEEFVSPVRGTPRCCRKCEPGNGMLRLCSNTEDTQCRPCKPGFEFSPFRSATKKCLQCRRCEELHPLAKTRNECTPVTDTICQCEKPYYMSEKEQTCKPCTVCKPGEGIVQACGWNSDTQCQSCPAGFWSAQSIDNVKCIPCQSCGKDQILVKECSSTSDTLCCPVNNPNCTHELSMYFGKITKII : OrigSeq

Jnet : ---HHHHHH-------EEE-------EE-----------------------------------------------------------------------EE--------EEE----------------EEE----------EE---------------------------------------------------------------------------------------------------------------EEEEE-------EEE----------------------- : Jnet

jhmm : ---EEEE---------HH--------EE---------------------------------------------------------------------------------EE-----------------EEE----------------------------------------------------------------------------------------------------------------------------EEE--------EEE----------------------- : jhmm

jpssm : --HHHHHHHHH----EEEEE-----HHHHHHH----EE--HH----------------------------------------------EE--------EEEE-------EEE----------------EEEE--------EEEE-----------------------HHHHHHHHHH-----------------------HHHH----------EE-----------------------------EE-------EEEEE-------EEE-------------------EE-- : jpssm

Lupas 14 : ---------------------------------------------------------------------------------------------------------------------------------------------------------------------------------------------------------------------------------------------------------------------------------------------------- : Lupas 14

Lupas 21 : ---------------------------------------------------------------------------------------------------------------------------------------------------------------------------------------------------------------------------------------------------------------------------------------------------- : Lupas 21

Lupas 28 : ---------------------------------------------------------------------------------------------------------------------------------------------------------------------------------------------------------------------------------------------------------------------------------------------------- : Lupas 28

Jnet_25 : --BBB-BBB-B---B-BBB-BB--B-BBB---BB-B-B-BBBBB-BBBB-BBB-B-BBBBB-BBBBBBB-BBBBB---B-BBBBBBBBB-B-B-BB-B-B--B--B---B-B-------BB--B--B-BBB--B-------B--B-----BB--------B--B--B-----B-BB---BB-B--BBBBBB--BB----B-----B--B----BBB----------B--B--BBBBB--B----B--B--B-----BB--B---BBBBBB----B-B----B---B-B--B- : Jnet_25

Jnet_5 : -------B----------------B-B--------B----BBB--B----------B----------------B-------B------B-B-----------B------B----------B--------------------B--------B---------B-------------------------------------------------------------------------B---------B-----------------------B------------------B---- : Jnet_5

Jnet_0 : ---------------------------------------------------------------------------------------------------------------------------------------------------------------------------------------------------------------------------------------------------------------------------------------------------- : Jnet_0

Jnet Rel : 9324333311327851233047775111133447743320331367764101356414677776777777777777767777765211234677763010012688772461367776535677776346514677777512113677652246777776411576532111112230367777777777777777765332221577777751331467777765456777641466777776511025777318881377877246436777766776777775323427 : Jnet Rel

: 1---------11--------21--------31--------41--------51--------61--------71--------81--------91--------101-------111-------121-------131-------141-------151-------161-------171-------181-------191-------201-------211-------221-------231-------241-------251-------261-------271-------281-------29 :

> Schistosoma matheei

Jnet Rel : 827432110115777776535677731010212462331122257777777777777777777776777775310137773221466777677633146777667777634613677765356777763101346777776321346777654567777763221256776777652256777777777777777777777777777777777777777777777767777764133667776776535677773123203777774312367777667777777777777777777777767777764120467776654104677777665555665667677777777777777777777777777777333777777777764146898875412888761278999831140335211121577777777622226761237999999999886441899987516212377775278999999999986203423565316787764120377777763112499999985318737899999987517843267666503222147787776511031123577777742278 : Jnet Rel
 : 1---------11--------21--------31--------41--------51--------61--------71--------81--------91--------101-------111-------121-------131-------141-------151-------161-------171-------181-------191-------201-------211-------221-------231-------241-------251-------261-------271-------281-------291-------301-------311-------321-------331-------341-------351-------361-------371-------381-------391-------401-------411-------421-------431-------441-------451-------461-------471-------481-------491-------501-------511-------521-------531-------541-------551-------561-------571-------581-------591------- :

OrigSeq : MYQTYQSVHSFDAHTKSTIPYMYNFKVFIEFLCLTIVWNSVIAVPLIFQSETMDGKTYPTVNTIAQMNNSNFTINSNENNETVTTTVSSIEGTGGVVEIQTETCDDPLKEFVSPVRGIPRCCRKCEPGNGMLRLCSNAEDTQCRPCKPGFEFSPFRSATKKCLHCRRCEEVHPLAKTRNECTPITDTICQCEKPYYMSEKEQTCKPCTVCKPGEGIVQACGWNSDTQCQSCPAGFWSAQSIDNVKCIPCQSCGKDQVLVKECSSTSDTLCCPLNNPNCTHELSMYFDYSAYDQESDISDNNNKSNQMLPIYCSIMGLIIISLLCYVVYKLWRQREASKNAKLTDSYNSNKTDLLDRTSCLDNNHLQHRRISSGFINNNNNAHLPNHSPQSTTELNNTINSTPDNDINNHLQFNDIIVGHEKAPLLGKLDHSNSSFSNFEQKPISVIPMNILGVICYRLSQHGWQELANIMDLETSKFDQLPPEVTSDLLSAAMEAQNTVESHLKLCNQDNSNTIQSITNNNPKNNLTMTVSMFQYMCLQNTVNLGQLMNSLQKLNRSDLVALIQQHTGIIKSKKSINHSNEEYKDKTKSMKSKENFQIEN : OrigSeq

Jnet : --HHHHHHH------------------EE---------------------------------------------E-----------------------------------EEE-----------------------------------------------------------------------------------------------------------------------------------------------EEE--------------------------------------------------------------------------------------------------------------------------------HHHHHHHHHHH------HHHHHHHHHHHHH--------------------------HHHHHHHHHHHHHHHHHHHHHHHHHH------------HHHHHHHHHHHHHHHHHHHHHHHH--------EEE---------HHHHHHHHHHHHH---HHHHHHHHHHHHH----HHHHHHH---EEE------------HHH-------------- : Jnet

jhmm : --H-----------------------------------------------------------------------------------------------------------EE--------------------------------------------------------------------------------------------------------------------------------------------------------------------------------------------------------------------------------------------------------------------EEE------------HHHHHHHHHHHH-----HHHHHHHH---HHHH------------------------HHHHHHHHHHHHHHHHHHHHHHHHHH--HHHH------HHHHHHHHHHHHHHHHHHHHHHHH--------EEEE--------EEEEHHHHHHHHHH--HHHHHHHHHHHHH----HHHHHHH---EEE------------HHH-------------- : jhmm

jpssm : --HHHHHHHH----------------EEEEEEE---HHHHHH------------------------------EEEE-----EE------------E--------------EEE----------------EEEE---------EEE-----------------EE-----------------------------------------------------------------------E-------------------EEEEE-------EEE----------------------------------------E----------------------------------------------------------------------------HHHHHHHHH---------HHHHHHHHHHH----HHHHH------------EE----HHHHHHHHHHHHHHHHH-HHHHHHHH------------HHHHHHHHHHHHHHHH--HHHHHH--------------------HHHHHHHHHHHH-----HHHHHHHHHHH-----HHHHHHHHHHEEE------------HHH----------E--- : jpssm

Lupas 14 : ---------------------------------------------------------------------------------------------------------------------------------------------------------------------------------------------------------------------------------------------------------------------------------------------------------------------------------------------------------------------------------------------------------------------------------------------------------------------------------------------------------------------------------------------------------------CCCCCCCCCCCCCC------------------------------------------- : Lupas 14

Lupas 21 : ------------------------------------------------------------------------------------------------------------------------------------------------------------------------------------------------------------------------------------------------------------------------------------------------------------------------------------------------------------------------------------------------------------------------------------------------------------------------------------------------------------------------------------------------------------------------------------------------------------------------ : Lupas 21

Lupas 28 : ------------------------------------------------------------------------------------------------------------------------------------------------------------------------------------------------------------------------------------------------------------------------------------------------------------------------------------------------------------------------------------------------------------------------------------------------------------------------------------------------------------------------------------------------------------------------------------------------------------------------ : Lupas 28

Jnet_25 : -B-BB--B-BB------BB-B-B-B-BBB-BBB--BBB-BBBBB-BBBBB-BB--B-B-BBBBBBBBB-BBBBBBBB----BBBBBBBBB-BB-BBB-B---BB--BB--BBB----B--BB--B--B-BB---B--B--B-B--B--B--BB-B-BBB-BB--B--BB-BB-B-BB-B-BB-B-BBBBBB--B-BBBB-BB---BBB-B--BBBBB--B----------B--BBBB---B----B--B--B---BBBB--B---BBBBBB-B--BBB----BBBB-BBBBB--B-BB--------BBB-BBBBB---BBB--B--BB-----B-----B-B--B--B--B-------B--------------B----B-B-BB------B--B---B-------B---B--B-B-BBB--B-BB--B-B--B-B--B----BBBBB--BB-BBBB-BB---B--B--B--B-B------B--BB--BB-BB--B---B--BB-BB-------B--B--------B-BBBBBB-BBBB--BB-BB-BB--B--B----BBBBB--BB-BB-----B------B---B--B-----B-B-- : Jnet_25

Jnet_5 : --------------------B-----------B--B---BBB-----B---BB-------------B---B--B-------B---------------------B-----------------B--B-----------------B------------------B--B---------------B----------------------------B-----------------------B-B---------B-----------------------B-----------------------------------------------------------------------------------------------------------------------------------------------------------------------------------B----B--B-------B---------------------B--------------B----------------------B---B-BB---B------B--B---B-------BB-BB------------------------------------- : Jnet_5

Jnet_0 : -----------------------------------------------------------------------------------------------------------------------------------------------------------------------------------------------------------------------------------------------------------------------------B------------------------------------------------------------------------------------------------------------------------------------------------------------------------------------------------------------------------------------------------------------------------------------------------------------------------------------------ : Jnet_0

Jnet Rel : 827432110115777776535677731010212462331122257777777777777777777776777775310137773221466777677633146777667777634613677765356777763101346777776321346777654567777763221256776777652256777777777777777777777777777777777777777777777767777764133667776776535677773123203777774312367777667777777777777777777777767777764120467776654104677777665555665667677777777777777777777777777777333777777777764146898875412888761278999831140335211121577777777622226761237999999999886441899987516212377775278999999999986203423565316787764120377777763112499999985318737899999987517843267666503222147787776511031123577777742278 : Jnet Rel

: 1---------11--------21--------31--------41--------51--------61--------71--------81--------91--------101-------111-------121-------131-------141-------151-------161-------171-------181-------191-------201-------211-------221-------231-------241-------251-------261-------271-------281-------291-------301-------311-------321-------331-------341-------351-------361-------371-------381-------391-------401-------411-------421-------431-------441-------451-------461-------471-------481-------491-------501-------511-------521-------531-------541-------551-------561-------571-------581-------591------- :

> Clonorchis sinensis

Jnet Rel : 999875321112315631115677776777777777777767777776330467777676667777631771367776311257776322112677776310035777322146777776322056665112313310133677753335677777777777777777777777777777777777777777722766777776322257777643112477776216436777766777777777777777777777753335677777777777777777777777777765323455666666777544324678776331000001100113211439944420315750112223478873100120123456661157777777001111121111111111101632562023232467776644421214443002244434566513227888860367776667777762039999999999999999941103478887408999999999860687008999999875178432566666511132577622467777777889 : Jnet Rel
 : 1---------11--------21--------31--------41--------51--------61--------71--------81--------91--------101-------111-------121-------131-------141-------151-------161-------171-------181-------191-------201-------211-------221-------231-------241-------251-------261-------271-------281-------291-------301-------311-------321-------331-------341-------351-------361-------371-------381-------391-------401-------411-------421-------431-------441-------451-------461-------471-------481-------491-------501-------511-------521-------531-------541-------551-------561-------571--- :

OrigSeq : MNFTKSAFVLSCLLALAYSSPVNNYSSRESLNGTEGAPKKVQHVSAGNKTVVTEFDEQHLEICPGPMQEFVSPVRGSPRCCRMCGPGTGMLRLCTDTDDTQCIGCEPGVEFSPTTSATLKCQQCRRCQDIHPLATTRIVCTPTTDTECGCMKGYYMSVNNQTCKPCTVCKPNEGVIRSCEWNADTQCQACPAGFWSASVGDTVKCIPCKTCSENEVVVRTCRENEDALCCPKTNVNCTLSPVFGFAPAKAGNETKTVNLTTLTPFETQQPADDIWMDGWPKKLTINGSSFYPPVQTAFHIDYTRYEQNGEVSDTSSKQNQMLPIYCSIMGFIIVFLLLYVVYKLWKQREAMTNAKLCEVYTSSGYSTVKLPVNSTQMDSVLNGPCGGDGNGAADHVKSEHISRLTTKHLCGTNRQQERDPLIANFETGASELSYLEIQLVTLQRDVLGMICFQLSRSGWRELATNMDIPTTSLLGPSANDSEFATQLAQAAQEAKHLILKESATQNSPDEDTVKASARLLAKLCQQPTANVRVLLAELERINRSDIIAFISDKLTKVTSSPAIPISSWSATNDPRV : OrigSeq

Jnet : --------------------------------------------------------------------EEEE----------------EEE---------------------------------------------------------------------------------------------------------------------------------------EEE-----------------------------------------------------------------------------------------------HHHHHHHHHHHHHHHHHHHHHHHH-------------------EHH-HHHHHHHHHH----------HHHHHHHHHHHHHHHHHHH---HHHH-----------------EEEEEEEEHHHHHHHHHHHH----HHHHHHH---------------HHHHHHHHHHHHHHHHHHHHHH----------HHHHHHHHHHHH-----HHHHHHHHHHHH-----HHHHHHHH---------------------- : Jnet

jhmm : ---------------------------------------------------------------------EE--------------------------------------------------------------------------------------------------------------------------EE--------------------------------EE-----------------------------------------------------------------------------------------------------EEEEEE-----HHEE----------------------HHHHHHHHHHHHH----------HHHHHHHHHHHHHHHHHHHHH--HHH-------------------EEEEEEEHHHHHHHHHHHH----HHHHHH------------------HHHHHHHHHHHHHHHHH-------------HHHHHHHHHHHH------HHHHHHHHHHH-----HHHHHHH----------------------- : jhmm

jpssm : ------EEEEEEE---EEE------------------------------E------------------EEEE-------E--------EEEE--------EEEE-----EE----------EE-----------EE---------------------------------------------------------------------EE--------EEEE-------EEE---------------------------------------------------------------------------------------------HHHHHHHHHHHHHHHHHHHHHHHHHHH-----HHHHHH------EEE-------HHHHH--------------------------------HHHHHHHHH------------EEEHHHHEH---HHHHHHHHHH--HHHHHHH---------------HHHHHHHHHHHHHHHHHHHHHHHH-------HHHHHHHHHHHHH-----HHHHHHHHHHH-----HHHHHHHHHHEE------------------- : jpssm

Lupas 14 : -----------------------------------------------------------------------------------------------------------------------------------------------------------------------------------------------------------------------------------------------------------------------------------------------------------------------------------------------------------------------------------------------------------------------------------------------cccccccccccccc----------------------------------------------------------------------------------------------------------------------------------- : Lupas 14

Lupas 21 : ------------------------------------------------------------------------------------------------------------------------------------------------------------------------------------------------------------------------------------------------------------------------------------------------------------------------------------------------------------------------------------------------------------------------------------------------------------------------------------------------------------------------------------------------------------------------------------------------ : Lupas 21

Lupas 28 : ------------------------------------------------------------------------------------------------------------------------------------------------------------------------------------------------------------------------------------------------------------------------------------------------------------------------------------------------------------------------------------------------------------------------------------------------------------------------------------------------------------------------------------------------------------------------------------------------ : Lupas 28

Jnet_25 : --B---BBBBBBBBBBBBBBBBB-BB---BB-BB-BBB--B--B--B--BBBBBB----B-BB-B--B-B-B-------BB--B--B-BB---B-----BBBBBB--B-BBB-----B--BBBB-BB-----B--BBB-B-BBBBBBBBBB--B-BBB-B-BBB-BB-BBBBBBBBBBBB-----BB--B--BBBB--------B--B--B-----BB--B--BBBBBBBB--BBBBBBB-BBBBBBB--B-----B-BBBBBB-----B---BBB--BB-B-BB-----BBB--BB---B-BB-------B-B-B----BBBBBBBBBBBBBBBBBBBBBB-BB----BB----BB-BB------BB-B-B-B--B--BB-BBB---B-BB---B----B--BB--BBB-B--------BBB-B--BB--B-BB-B-BBBB---BBBBBBBBBB--BB--BB--B-B-BB-B-B-------BB--BB-BB--B-BBB--------------B-BBB-BBB-BB--B-B-B-BBBB-B--B----BBBBB---B--B-----B-B--B-------- : Jnet_25

Jnet_5 : -------BBBBB-B-BB-BBB---------B---------B----------------------------B----------B--B-----------------B------------------B-------------------------------------------------------------------------B---------B---------------------B-B-------B-----------------------------------------------------------B----------------------------B--B-BBBB-B----B--------------------------------------------------------------------------------B----------------B--B---BB-BBB--B-------B------------------------B----------B------------------B-BB---B------B--BB--B-------BB--B---B----------B----------- : Jnet_5

Jnet_0 : ---------B-----B---------------------------------------------------------------------------------------------------------------------------------------------------------------------------------------------------------------------------------------------------------------------------------------------------------------------------BB-------B----------------------------------------------------------------------------------------------------------B-BB-------------------------------------------------------------------------------B--B---------------B-------------------------- : Jnet_0

Jnet Rel : 999875321112315631115677776777777777777767777776330467777676667777631771367776311257776322112677776310035777322146777776322056665112313310133677753335677777777777777777777777777777777777777777722766777776322257777643112477776216436777766777777777777777777777753335677777777777777777777777777765323455666666777544324678776331000001100113211439944420315750112223478873100120123456661157777777001111121111111111101632562023232467776644421214443002244434566513227888860367776667777762039999999999999999941103478887408999999999860687008999999875178432566666511132577622467777777889 : Jnet Rel

: 1---------11--------21--------31--------41--------51--------61--------71--------81--------91--------101-------111-------121-------131-------141-------151-------161-------171-------181-------191-------201-------211-------221-------231-------241-------251-------261-------271-------281-------291-------301-------311-------321-------331-------341-------351-------361-------371-------381-------391-------401-------411-------421-------431-------441-------451-------461-------471-------481-------491-------501-------511-------521-------531-------541-------551-------561-------571--- :

> Trichobilharzia regenti

Jnet Rel : 8988777777764011267888742121101123367752467777666545667776777777767777764133677631134677766676641466767777642114667776522467777631002057777643123677654566777677777777776533423222057777751133677765356777654566777677654567777777777654567776520246777765320257773111113778772101367777667777777777777777777777767777766414667776777767777766414667776777776545667776677777654567776331577777642288879988603677766511227663212233677777710112211212347537889999874371788872157777777621378999999994998753454898887433301273576034322788898888887405778777614788888157777776603222333144048 : Jnet Rel
 : 1---------11--------21--------31--------41--------51--------61--------71--------81--------91--------101-------111-------121-------131-------141-------151-------161-------171-------181-------191-------201-------211-------221-------231-------241-------251-------261-------271-------281-------291-------301-------311-------321-------331-------341-------351-------361-------371-------381-------391-------401-------411-------421-------431-------441-------451-------461-------471-------481-------491-------501-------511-------521-------531-------541-------551-------561------- :

OrigSeq : MSSNYHKSIHHSLKHTIHCIHRTELLMKLLYFIVLWNSVIAGPMLFHSDTLENKDSLPGNPVLTQTTDKYRILIYFDESKEVVTRSIQQHERKKGDICLQAETCDDPFEEFVSPVRGTPRCCRKCDVGNGMLRLCSNTENTQCRPCKSGFEFSSVRSATKKCMQCRRCEELHPLAKTRTECTPTTDTICQCEKPFYMSEKEQTCKPCTACKPGEGIVKLKIQQCDWNSDTQCQSCPAGFWSAQTVDNVKCIPCQICEKNQILVRECSPTTDTLCCPVNNPNCTHEYALPLDYPTYDQESNMSDNGNKPNQMLPIYCSIMGLIIISLLAYVVYKLWKQREASKNAKLSDAYNIGCNKNNLLDRTTCVDNHHFQHHMISSDLLNHSPQSITEITKEASNNNNNNNNNDLILGHEKEPLLGKSVGLNNSSDCLEQPLTVVPMNILGVICYRLSQHGWQDLANTMNHNPSKQNTWELQEALLSLLYFFAYQVEHSASNLRVLLTNIKCRIIHMDSPMVINRCSYLVLSATVKIFRSDQQSDHATEIYILTLTKYNSKLNSTEYRIRLFSFTYLVL : OrigSeq

Jnet : --------------------------------------------------------------------------------------------------------------------------------------------------------------------------------------------------------------------------------------------------------------------EEE--------------------------------------------------------------------------------------------------------------------------HHHHHHHHHH-------------------------------HHHHHHHHH-----HHHHHHHHHHH--HHHHHHH------------HHHHHHHHHHHHHHHHHHHHHHHHHHHHHHHHEEEE----HHHHHHHHHHHHHHHHHHH---------EEEEEEEE---------HHHHHHHHHHHH-- : Jnet

jhmm : --------------------------------------------------------------------------------------------------------------------------------------------------------------------------------------------------------------------------------------------------------------------------------------------------------------------------------------------------------------------------------------------------HHHHHHHHH---------HHHH-----------------HHHHHHHHHH-----HHHHHHHHHHH--HHHHHH-----------HHHHHHHHHHHHH-HHHHHHHHHHHHHHHHHHHHHHEE----HHHHHHHHHHHHHHHHHHH---------EEEEEEE----------HHHHHHHHHHH--- : jhmm

jpssm : --------------EE--------EEEHHHHHHH----------------------------------------E------EEE---------------------------------------------EEEE--------EEE------------------------------EEEE------------------------------------------------------------------------EE-------EEEEE-------EEE----------------------------------------------------------------------------------------------------E---------HHHHHHHHHHHH----------------HHHHH-----------------EE----HHHHHHHHHHH---HHHHHH-------------HHHHHHHHHHHHHHHH--HHHHHHHHH--EEEEEE----HHHHHHHHHHHHHHHHHHH---------EEEEEEEE----------EEEEEE-HHHH-- : jpssm

Lupas 14 : ------------------------------------------------------------------------------------------------------------------------------------------------------------------------------------------------------------------------------------------------------------------------------------------------------------------------------------------------------------------------------------------------------------------------------------------------------------------------------------------------------------------------------------------------------------------------------------------- : Lupas 14

Lupas 21 : ------------------------------------------------------------------------------------------------------------------------------------------------------------------------------------------------------------------------------------------------------------------------------------------------------------------------------------------------------------------------------------------------------------------------------------------------------------------------------------------------------------------------------------------------------------------------------------------- : Lupas 21

Lupas 28 : ------------------------------------------------------------------------------------------------------------------------------------------------------------------------------------------------------------------------------------------------------------------------------------------------------------------------------------------------------------------------------------------------------------------------------------------------------------------------------------------------------------------------------------------------------------------------------------------- : Lupas 28

Jnet_25 : ----B---B---B--BB-B-----BBBBBBBB-BBB-BBBBBBBBBBB-BB-B--BB--BBBBB-BB--B-BBBBB--B-BBB--BBB-B-B--B--B--B--B--B--BBBBBB-B--BBB--B--B-BBB--B-----B-B--B--BBBBB-B-B-BB-BBBBBBB--B-----BB--B-BB-BBB-BBB---B-B--B--B--B-----B--BB-BBBBB---B----B--B--BBBBB--B----B--B--B----BBB--B---BBBBBB-B-BB-BB--BBBBB-BBB-B--B-BB--BB-B-BBBB--BBBBBBBB-BB-BB--B--------B-------B-----------BB-BB----------B---BB-B-------B---B--B-----B-BBBB-B----BBB-B-----BB-BBBB-B--B-B-BBBBBBBBB-------BB-BB--------B--B---BB--BB-B---B--BB--B--B---B-BBBB-B-B-BBB-BBBBBBBBBBB-BB--------BBBBBBBBBB-B---B----BBBBBB-BBB--- : Jnet_25

Jnet_5 : ------------------B-----B-B-B----B---BBB-B-BBB----------B--------B------B--B------B--------------------B--------------------B----------------------------------------------------------------------------------------------------------------BBB---------B----------------------BB------------------------------------B----B------B-------------------------------------------------------------------B---------------BB-------B-------------B-----------B-BBBB-B-------B---B---------------B---B------B------B--B---B-B-B-------BB--B--BBB---B-B------------BBBB-B-------------B-B-------- : Jnet_5

Jnet_0 : ---------------------------------------------------------------------------------------------------------------------------------------------------------------------------------------------------------------------------------------------------------------------------------B-----------------------------------------------------------------------------------------------------------------------------------------------------------------------B-BB------------------------------------------------------------------------------------------------------------------------------ : Jnet_0

Jnet Rel : 8988777777764011267888742121101123367752467777666545667776777777767777764133677631134677766676641466767777642114667776522467777631002057777643123677654566777677777777776533423222057777751133677765356777654566777677654567777777777654567776520246777765320257773111113778772101367777667777777777777777777777767777766414667776777767777766414667776777776545667776677777654567776331577777642288879988603677766511227663212233677777710112211212347537889999874371788872157777777621378999999994998753454898887433301273576034322788898888887405778777614788888157777776603222333144048 : Jnet Rel

: 1---------11--------21--------31--------41--------51--------61--------71--------81--------91--------101-------111-------121-------131-------141-------151-------161-------171-------181-------191-------201-------211-------221-------231-------241-------251-------261-------271-------281-------291-------301-------311-------321-------331-------341-------351-------361-------371-------381-------391-------401-------411-------421-------431-------441-------451-------461-------471-------481-------491-------501-------511-------521-------531-------541-------551-------561------- :

> Schistosoma margrebowiei

Jnet Rel : 86012301135677776421036775101466777677777777777777777777777777777777777777777777777777767777652125667666777631001367776535677776311136677777641234677721012677776322135565344445677777777777777777777777777777777777776666666677767777776322367777677653567777312321367777630136777766777777777777777777777776777776413366777643133467777776554454456767777777710001277777777777777743222777777777013333222111111111213777646523113016611577777777623226701237999910999886441899988542101777765278999999999986203423565316787764120377777764001499999985318737899999987517843267666503311147787766413211024577777642278 : Jnet Rel
 : 1---------11--------21--------31--------41--------51--------61--------71--------81--------91--------101-------111-------121-------131-------141-------151-------161-------171-------181-------191-------201-------211-------221-------231-------241-------251-------261-------271-------281-------291-------301-------311-------321-------331-------341-------351-------361-------371-------381-------391-------401-------411-------421-------431-------441-------451-------461-------471-------481-------491-------501-------511-------521-------531-------541-------551-------561-------571-------581-------591------ :

OrigSeq : MYQTYQSVHSFDAHTKRTITCMYNFKVFIEFLCLTIVWNSVIAVPLIFQSETPEGKTYPTVNTTAQMNNSNYTISSNEYNETVTTTVNSIEGTGGVVEIQTETCDDPLKEFVSPVRGIPRCCRKCEPGNGMLRLCSNSEDTQCRPCKPGFEFSPFRSATKKCLHCRRCEEIHPLAKTRNECTPITDTICQCEKPYYMSEKEQTCKPCTVCKPGEGIVQACGWNSDTQCQSCPAGFWSAQSIDNVKCIPCQSCGKDQVLVKECSSTSDTLCCPLNNPNCTHELSMYFDYSAYDQESDISDNNNKSNQMLPIYCSIMGLIIISLLCYVVYKLWRQREASKNAKLTDSYNSNKTDLLDRTSCLDNNHLQHRRISSGFINNNNAHLPNHSPQSTTELNNTINSTPDNDINNHLQFNDIIVGHEKAPLLGKLDHSNSSFSNFEQKPISVIPTNILGVICYRLSQHGWQELANIMDLETSKFDQLPSEVTSDLLSAAMEAQNTVESHLKLCNQDNSNTIQSITNNNPKNNLTMTVSMFQYMCLQNTVNLGQLMNSLQKLNRSDLVALIQQHTGIIKSKKSINHSNEEYKNKTKSIKSKENFQIEN : OrigSeq

Jnet : -------------------E------------------------------------------------------------------------------------------EE------------------------------------------------------------------------------------------------------------------------------------------------EEE---------E----------------------------------------------------------------------------------------------------------------------HHHHHHHHHHHHHHHHHHHHHHHHHHHHHH--HHHHH-----------------HHHHHHHHHHHHHHHHHHHHHHHHHHHHHH---------HHHHHHHHHHHHHHHHHHHHHHHH--------EEE---------HHHHHHHHHHHHH---HHHHHHHHHHHHH----HHHHHHH---EEE---------HHHH---------------- : Jnet

jhmm : ---------------------------------------------------------------------------------------------------------------------------------------------------------------------------------------------------------------------------------------------------------------------------------------------------------------------------------------------------------------HHHHHH---------------HHHHH---------HHHHHHHHHHHHHHHHHHHHHHHHHHHHHHHH---HH------------------HHHHHHHHH--HHHHHHHHHHHHHHHHHHHHH-------HHHHHHHHHHHHHHHHHHHHHHHH--------EEEE--------EEEEHHHHHHHHHH--HHHHHHHHHHHHH----HHHHHHH---EEE----------H------------------ : jhmm

jpssm : ---HHH------------EEE----------------------------------------------------------------------------------------EEEE----------------EEE-----------EE------EEE--------EE----------------------------------------------------------------------EE-------------------EEEEE--------EE----------------------------------------E--------E-EE------------------------------------------------------------------EE--------------HHHHHHHHHH----HHHHH------------EE----HHHHHHHHHHHHHHHHH-HHHHHHHH------------HHHHHHHHHHHHHHHH--HHHHHH--------------------HHHHHHHHHHHH-----HHHHHHHHHHH-----HHHHHHHHHHEEE---------HHHHH-----------E--- : jpssm

Lupas 14 : --------------------------------------------------------------------------------------------------------------------------------------------------------------------------------------------------------------------------------------------------------------------------------------------------------------------------------------------------------------------------------------------------------------------------------------------------------------------------------------------------------------------------------------------------------------CCCCCCCCCCCCCC------------------------------------------- : Lupas 14

Lupas 21 : ----------------------------------------------------------------------------------------------------------------------------------------------------------------------------------------------------------------------------------------------------------------------------------------------------------------------------------------------------------------------------------------------------------------------------------------------------------------------------------------------------------------------------------------------------------------------------------------------------------------------- : Lupas 21

Lupas 28 : ----------------------------------------------------------------------------------------------------------------------------------------------------------------------------------------------------------------------------------------------------------------------------------------------------------------------------------------------------------------------------------------------------------------------------------------------------------------------------------------------------------------------------------------------------------------------------------------------------------------------- : Lupas 28

Jnet_25 : -B--B-BBBBB------BB-B---B-BBB--BBB-BBB-BBBBB-BB--B-BB-B-BBBBB--B-BBBBBBBBBB-B-B--BBBB-B-BB-B-BBBBBB-B-BB--B--BBBB---B---BB--B--B-BBB--B--B--B-B--B----BBB-B--BB--B--B--BBBBBBBBBBB-BB--BBBBBBBBB-B-BBBBBBB--B-B--B--B-BBB-BB--B-BB----B--BBBB--------B--B--B----BBB--B---BBBBBB-B--BBBB--BBBBBBBBBBBB----B----BB-BBBB-BBBBBBBBBBBBBB--BB-B-----B---B-BB-------B-BB---B----------------B---B--B---B--BB--B---B-------B---B--B-B-BBB--B-BB--B-B--BBB--B----BB-BB--BBBBBBB-BB--BB--BB-B--B-B------B--BB--BB-BB--B---B--BB-BB------BB--B--------B-BBBBBB-BBBB--BB-BB-BB--B--B----BBBBB--BB-BB-----B------B---B--B-----B-B-- : Jnet_25

Jnet_5 : -------B---------BB-B-----B-----B------B--B---B--B----------------B------B-----------------------------B-----------------B--------------------B------------------B-------------------------------------------------------------------------B---------------------------------B-------------------------------------B--B-BB----BB--------------------------------------------------------------------------------------------------------------------B----B--B---B----B--B-------B---------------------B--------------B----------------------B---B-BB---B------B--B---B-------B--BB------------------------------------- : Jnet_5

Jnet_0 : -------B---------------------------------------------------------------------------------------------------------------------------------------------------------------------------------------------------------------------------------------------------------------------B----------------------------------------------------------------------------------------------------------------------------------------------------------------------------------------------------------------------------------------------------------------------------------------------------------------------------------------- : Jnet_0

Jnet Rel : 86012301135677776421036775101466777677777777777777777777777777777777777777777777777777767777652125667666777631001367776535677776311136677777641234677721012677776322135565344445677777777777777777777777777777777777776666666677767777776322367777677653567777312321367777630136777766777777777777777777777776777776413366777643133467777776554454456767777777710001277777777777777743222777777777013333222111111111213777646523113016611577777777623226701237999910999886441899988542101777765278999999999986203423565316787764120377777764001499999985318737899999987517843267666503311147787766413211024577777642278 : Jnet Rel

: 1---------11--------21--------31--------41--------51--------61--------71--------81--------91--------101-------111-------121-------131-------141-------151-------161-------171-------181-------191-------201-------211-------221-------231-------241-------251-------261-------271-------281-------291-------301-------311-------321-------331-------341-------351-------361-------371-------381-------391-------401-------411-------421-------431-------441-------451-------461-------471-------481-------491-------501-------511-------521-------531-------541-------551-------561-------571-------581-------591------ :

> Gyrodactylus salaris

Jnet Rel : 8856777776312534777221267777667777777777765456677767777777777777677777531002478875301213677766777774312025777773100136777776357213677403667776777765356777731783037888731887136777766777777777777777777777666777773101035777201478774211101232011111111100216887226776316877771124545888887643334671122789999998877640201004325777775213346788752478999987434448999999985268999 : Jnet Rel
 : 1---------11--------21--------31--------41--------51--------61--------71--------81--------91--------101-------111-------121-------131-------141-------151-------161-------171-------181-------191-------201-------211-------221-------231-------241-------251-------261-------271-------281-------291-------301-------311-------321-------331-------341-------351-------361---- :

OrigSeq : MLKLCTDTEDTLCSACISNVTFSDQASAIGKCQDCSKCNEASSTLLIFTYRIFYFFLDKGQKYTTYKIDENFEVGIACSLTQDIKCKCKSGFYRENELESCLRCTSCERLNKSVIKECNDINGDSVCGNCDSNQFLEDGKSGNERVCRNCRVCDKDEIVLKNCTSTSNTECCRIDDLVCRGHIYPKHWFVRTAIAILQDRSTGVNSYNDNYIIIICCTILSLIILSTTLYILYKLLRQYRSGILKIAKRTSLRNERNYQIDALLTNKEGLHYSQEEKTYQEIKKVKVADIDYAKRTELKEILCRVEDEVKFVTILRVFDNSKFDDIEGWFNNSENRPYLLIKALRRINKKAEAKRIMNILKNYNVEK : OrigSeq

Jnet : ------------EEE---------------------------------------------------------------------EEE--------------------------EE----------EEE----------------------------EEEE--------EEEEE---------------------------------------EEE-------E-------HHHH----HHHHHHHHHHH--------EEEEEEE-------HHHHHHHHHHHHHHH-----HHHHHHHHHHHHHHHHHHHEEEEEEE-------------------HHHHHHHHHHHHHHHHHHHHHHHHHH----- : Jnet

jhmm : -------------EE--------------------------------------------------------------------------------------------------------------EE------------------------------EE----------EEE----------------------------------------------------------EE-------------------------EEEEEEE------HHHHHHHHHHHHHHHH-----HHHHHHHHHHHHHHHHHHEEEEEEEE-------------------HHHHHHHHHHHHHHHHHHHHHHHHHH----- : jhmm

jpssm : -----------EEEE-----EE-------------------------------------------------EEEEE------EEEEE-------------EEE---------EEEE---------EEEE---------------------------EEEEE-------EEEEE--------------------------------------EEEEE-----EE------HHHHHHHHHHHHHHHHHHHHHH------EEEEEE----------HHHHHHHHHHHHHH-------HHHHHHHHHHHHHHHHHEEEEHH-------------------HHHHHHHHHHH--HHHHHHHHHHHH------ : jpssm

Lupas 14 : ----------------------------------------------------------------------------------------------------------------------------------------------------------------------------------------------------------------------------------------------------------------------------------------------------------------------------------------------------cccccccccccccc------------- : Lupas 14

Lupas 21 : ------------------------------------------------------------------------------------------------------------------------------------------------------------------------------------------------------------------------------------------------------------------------------------------------------------------------------------------------------------------------------- : Lupas 21

Lupas 28 : ------------------------------------------------------------------------------------------------------------------------------------------------------------------------------------------------------------------------------------------------------------------------------------------------------------------------------------------------------------------------------- : Lupas 28

Jnet_25 : -B--B-------B--B--B--BB-BB-B---B--B--B--B--BBBBBBBBBBBBB---BBBB-BB-B--BBBB-B-----B-BBB-B--BBBBB-----B--B--B--B--BBBB-B------B-B--B--BBBBB------B--B--B--B----BBB--B----BBBB--B--B-BB-BBBB--BBBBBBBB-B-BBBB-BBBB---BBBBBBB--BBBBBB-B-B-BBB--B--B---BBBBB--B-B-B-B-B-B-BBB----BB--------B--B----B--B--B-B--B---B--B---B-BBBBBBBB----B--B--BB-----BBBBBB-BB--B---B-B--BB-BB--B-B-- : Jnet_25

Jnet_5 : ------------B--B---------------B--B----------------------------------------------------B------------B------------B---B--------B-------------------B-----------------------B--------------------B-----------B--------BB--------B-------B----B-----------------------B------------------------------------------------B--B-BB-B--------B------------BBB-BB-----------B--BB------- : Jnet_5

Jnet_0 : -------------------------------------------------------------------------------------------------------------------------------------------------------------------------------------------------------------------------------------------------------------------------------------------------------------------------------------------------------------------B----------- : Jnet_0

Jnet Rel : 8856777776312534777221267777667777777777765456677767777777777777677777531002478875301213677766777774312025777773100136777776357213677403667776777765356777731783037888731887136777766777777777777777777777666777773101035777201478774211101232011111111100216887226776316877771124545888887643334671122789999998877640201004325777775213346788752478999987434448999999985268999 : Jnet Rel

: 1---------11--------21--------31--------41--------51--------61--------71--------81--------91--------101-------111-------121-------131-------141-------151-------161-------171-------181-------191-------201-------211-------221-------231-------241-------251-------261-------271-------281-------291-------301-------311-------321-------331-------341-------351-------361---- :

> Hymenolepis diminuta

: 1---------11--------21--------31--------41--------51--------61--------71--------81--------91--------101-------111-------121-------131-------141-------151-------161-------171-------181-------191-------201-------211-------221-------231-------241-------251-------261-------271-------281-------291-------301-------311-------321-------331-------341-------351-------361-------371-------381-------391-------401-------411-------421-------431-------441-------451-------461-------471-------481-------491-------501-------511----- :
Jnet Rel : 83200001111111111110032678753123677776676777777633046777653567777631111267777631003577732223677777632236666666677777777777652256777766777776531256776513222315777763212156765456677777775333577730321136777763323677776677777776545667777766414677776414667776677776641467632132577754432223567777765210256776667777654567777776642132212220012113887601450220321016522567776546777766644533651467650026777642010677302899986764210200111016556777652200146777777651167899999312230156787703677777516887178999999981157777777777777889 : Jnet Rel

OrigSeq : MELISRLFSATWLLLFSLQCEFICSLPQLSTIDLNPIQQETCPVANEELISTLNGQLRCCKKCPVGEGMLQLCTNTTQTVCRPCQEGSEFSLEASATAKCMQCKQCQELHPFARFRKHCTPTSDAVCECVAGYFFIEVHSTCQSCTKCPPGYGAEKPCGWNEDSICSPCPEGTWSSTESATEKCLTCKRCKPGQIEMRSCTATQNTLCCPLHNPNCDDNYEDDEFDEMPPATSPHQQQQLGFFQMQVEINSPESTKAPTSNWVSFSEKGINHDLNGTLNSMNIQTAFRVEDPKTFQSVGHFSDGSFPQQMAYGDDYPMITIYCSLLGLVILTLLIYVFYKLWQQKLSMEDAKSIEAGVFHPAFTGIKTTGGKPKSSRLTVSVKDTNDKEHLLGESQQYSYAPQKSIPDHLLTELSQGLAVENHWKQVGTKLGFTEESLQKFEKPKISEDSGDAISAAKRMLTSWYSSRSSTDSSPLTSLLVVLESTSGTNELARRLKEYLKPSSAVTSTNSSQTSNNQ : OrigSeq

Jnet : ------HHHHHHHHHHHHHH-----------------------------------------------EEE---------------------------------------------------------------------------------------------------------------------------EEEE---------EE-----------------------------------------------------------------------------------------------------------------------EEEEE--HHHHHHHH-----HHHHHHH-------------------------EEEE------E---------------HHHHHHHHHHHH----HHHHH--------------------------HHHHHHHHHH------------HHHHHHHHHH----HHHHHHHHHHHH------------------ : Jnet

jhmm : --------------------------------------------------------------------------------------------------------------------------------------------------------------------------------------------------------------EE-----------------------------------------------------------------------------------------------------------------------EEEEE--EEEEHHHH-----HHHHHHHH------------------------EEEE-----EEE----------------HHHHHHHHHH---HHHHHHH--------------------------HHHHHHHH-------------HHHHHHHHHH----HHHHHHHHHHH------------------- : jhmm

jpssm : --HHHHHHHHHHHHHHHHHHHH------EEE------------------E-----------------EEEE--------EEEE-----EEE---------EE-------------------------------------------------EEEEE--------EEE--------------------------EEEEE--------EE------------------------------------------------------------EEE-----------------------------------------------------HHHHHHHHHHHHHHHHHHH----HHH-----------------------------EEEE--------------EEE-----HHHHHHHHHHH---------------------H--------------HHHHHHHHHHHHHH----------HHHHHHH------HHHHHHHHHHH------------------ : jpssm

Lupas 14 : -------------------------------------------------------------------------------------------------------------------------------------------------------------------------------------------------------------------------------------------------------------------------------------------------------------------------------------------------------------------------------------------------------------------------------------------------------------------------------------------------------------------------------------- : Lupas 14

Lupas 21 : -------------------------------------------------------------------------------------------------------------------------------------------------------------------------------------------------------------------------------------------------------------------------------------------------------------------------------------------------------------------------------------------------------------------------------------------------------------------------------------------------------------------------------------- : Lupas 21

Lupas 28 : -------------------------------------------------------------------------------------------------------------------------------------------------------------------------------------------------------------------------------------------------------------------------------------------------------------------------------------------------------------------------------------------------------------------------------------------------------------------------------------------------------------------------------------- : Lupas 28

Jnet_25 : ---B--BBBBBBBBBBBBBBBBBBBBBBBBBB-B--B---BB-BB---BBB---B-B-BB--B--B-BBB--B-----B-BBBB--B-BBB---B-B-BB--B-----BB-BBBBBB-BBBBBBBBB-BBBBBBB---B-BBB-BBB--B-BBBBB--------B---B--BBBBB--B----B--B--B----BBB--B-----BBB-BB-B--B---B----B-BBB--BBB-BBB-B--B---BBB-B--B--B----BB---B-BBB--B-B-B----B--BB-B----BB--B-BB--------BBB-----BB--BB-BBBBBBBBBB-BB-B--BB--BB--BB--B--BBB---B-BB----B---B--BBBBB-B------B------B-B-B---BB-BB---B--BB-B---BB-B-B-B-B----B--B----B------BB-BB-BBB-BBBB--B-----BB-BBBBBB-BB-----BB--B--BB----BB-B---------- : Jnet_25

Jnet_5 : ------BBB--B-B---B-----B----BB-----------B-----------------B--B-----------------B--------B---------B-------------------------B---------------------------------------------------------B-----------------------B-------------------------------------------------------------B---B--------------------B---------------------------B---B--B------B--------------------------------------------B-------------------------------B---------B--------------------------------B--BB--------------B-BBBBBB--------BB--B---------------------- : Jnet_5

Jnet_0 : --------------------------------------------------------------------------------------------------------------------------------------------------------------------------------------------------------------------------------------------------------------------------------------------------------------------------------------B--B------------------------------------------------------------------------------------------------------------------------------B---------------------B---B--------B---B---------------------- : Jnet_0

Jnet Rel : 83200001111111111110032678753123677776676777777633046777653567777631111267777631003577732223677777632236666666677777777777652256777766777776531256776513222315777763212156765456677777775333577730321136777763323677776677777776545667777766414677776414667776677776641467632132577754432223567777765210256776667777654567777776642132212220012113887601450220321016522567776546777766644533651467650026777642010677302899986764210200111016556777652200146777777651167899999312230156787703677777516887178999999981157777777777777889 : Jnet Rel

: 1---------11--------21--------31--------41--------51--------61--------71--------81--------91--------101-------111-------121-------131-------141-------151-------161-------171-------181-------191-------201-------211-------221-------231-------241-------251-------261-------271-------281-------291-------301-------311-------321-------331-------341-------351-------361-------371-------381-------391-------401-------411-------421-------431-------441-------451-------461-------471-------481-------491-------501-------511----- :

> Hymenolepis microstoma

Jnet Rel : 997776543012221111111111020423346777767767777776330467776413667776311112677776431135777322236777766322367777776400100367777776545676333156776777777777630301245677732134677631466777677653567777317871367777630136777766777777777777777767765456545667776777777721232101137887323578999999886245677777777777733777777777777777777777777766666777776408999999862368888761288887437618888860477643302143223222221066515787135899998873088872158999999987425435777766777889 : Jnet Rel
 : 1---------11--------21--------31--------41--------51--------61--------71--------81--------91--------101-------111-------121-------131-------141-------151-------161-------171-------181-------191-------201-------211-------221-------231-------241-------251-------261-------271-------281-------291-------301-------311-------321-------331-------341-------351-------361-------371-------381-------391-------401-------411-------421-------431-------441-------451--- :

OrigSeq : MGLISRLFSAAWILLFSFQFAFTFGLPQLSTIGLDPIQQETCPTTNEELVSTLNGQLRCCKKCPAGEGMLQLCTNTTQTVCRPCQEGSEFALEASATAKCMQCKQCQELHPFAKFRKHCTPTSDAVCECVAGYFFIEARSTCQSCTKCPPGYGAEKPCDWNEDSICSPCPKGTWSSTESATQKCLTCKKCKPGQIEMRPCTATQNTLCCPLHNPNCDENYEEDEFDDSKSLPGVGHLPENSFPPQFTYGDDYPMITIYCSLLGLVILTLLIYVFYKLWQQKLSIEDAKNIEAGIFHPAFTGIKTSGGKSKSSKLAVSAKDTNDRQHLLGESQQYSYDPQKSAIPDQLLMELSQGLAVDNRWKNVGTKLGFNEESLQNFEKPKISEDSGDVISVVKHMLTSWYSSRIYIDPSPLNSLLVVLESAPETNKLARHLKEYLKSSCAINSSTNSSPISNNQ : OrigSeq

Jnet : ---------HHHHHHHHHHHHHHH-------------------------------------------EEE---------------------------------------------------------------------------------------------------------------------------EEEEE--------E--------------------------------------------------------H-------HHHHHHHHHHHHHHH-------------------------------------------------------HHHHHHHHHH--------HHHHHHHH----HHHHHH------HHH----HHHHHHHHHHHHHH----HHHHHHHHHHHH------HHHHHHHHHHHHH----------------- : Jnet

jhmm : --------------------------------------------------------------------------------------------------------------------------------------------------------------------------------------------------EEE-----------------------------------------------------------EEEEHHHHHE-----HHHHHHHHHHHHHHH---------------EE--------------------------------------HHHHHHHHHH--------HHHHHHHH----HHHHHH------HH-----HHHHHHHH--HHHH----HHHHHHHHHHHHH-----HHHHHHHHHHHHH----------------- : jhmm

jpssm : --------HHHHHHHHHHHHHHHHHH---EE------------------E-----------------EEEE--------EEEE-----EEE---------EE-------------------------------EE------------------E----------EEE--------------------------EEEEE--------EE----------------------------------------------------------------HHHHHHHHHHHHH-------------------------------------------------------HHHHHHHHHHH---------HHHHHHH---HHHHHHHH-----HHHH---HHHHHHHHHHHHHH-----HHHHHHHHHHH------HHHHHHHHHHHHH----------------- : jpssm

Lupas 14 : ------------------------------------------------------------------------------------------------------------------------------------------------------------------------------------------------------------------------------------------------------------------------------------------------------------------------------------------------------------------------------------------------------------------------------------------------------------------------ : Lupas 14

Lupas 21 : ------------------------------------------------------------------------------------------------------------------------------------------------------------------------------------------------------------------------------------------------------------------------------------------------------------------------------------------------------------------------------------------------------------------------------------------------------------------------ : Lupas 21

Lupas 28 : ------------------------------------------------------------------------------------------------------------------------------------------------------------------------------------------------------------------------------------------------------------------------------------------------------------------------------------------------------------------------------------------------------------------------------------------------------------------------ : Lupas 28

Jnet_25 : ----B--BBBBBBBBBBBBBBBBBBBBBBBBBBB-BB--BBB------B-B-----B-BB--B--B-BB---B-----B-BBBB--B-BB----BBB--B--B-BBB---BBBBB---B--B---B--BB-BBBBB--B---B-BB-B---BBBB-BBB--BBBBB--BB-BBBB--------B--B--B----B-B--B----BBBB-BB--B-B---BB-------BB-BBB--BB-B-BBB-BBB---B-BBBBBBBBBB--B--B-BBBBB-BB--------B------B--B---B-------B--B-BBB-B--------B--------B-B-----B--BB--BB--------B--BB--B-B---BB--B----BB--BB---B-BBBBBBBBB---BBBB--BB-BBB-BB--B-----BB-BB--BB-BBB-B--------B---- : Jnet_25

Jnet_5 : --------BBB-B-B-B---B-B-----B------------------------------B-----------------------------B---------B---------------------------------BB--------------------------------------B---------------------------------B---------------------------------B-----------B--B-B--B---------------B------------------------------------B--------------------------------B---------------BB--B-------------------------B--BB-------------B--BB--BB--------BB--B---B---B--------------- : Jnet_5

Jnet_0 : -----------------------------------------------------------------------------------------------------------------------------------------------------------------------------------------------------------------------------------------------------------------------------------------------------------------------------------------------------------B---------------------------------------------B--B------------------B--B-------------B----------------------- : Jnet_0

Jnet Rel : 997776543012221111111111020423346777767767777776330467776413667776311112677776431135777322236777766322367777776400100367777776545676333156776777777777630301245677732134677631466777677653567777317871367777630136777766777777777777777767765456545667776777777721232101137887323578999999886245677777777777733777777777777777777777777766666777776408999999862368888761288887437618888860477643302143223222221066515787135899998873088872158999999987425435777766777889 : Jnet Rel

: 1---------11--------21--------31--------41--------51--------61--------71--------81--------91--------101-------111-------121-------131-------141-------151-------161-------171-------181-------191-------201-------211-------221-------231-------241-------251-------261-------271-------281-------291-------301-------311-------321-------331-------341-------351-------361-------371-------381-------391-------401-------411-------421-------431-------441-------451--- :

> Hymenolepis nana (New Assembly)

Jnet Rel : 999843011111111112221110023212225777776677777777777777777766677776311034677776642025777300236777776322012677764233036777777777777777654567777777777765211122157777777777777777777777777653567777317820367777633236777766777777777677777664123566767677766677771146201333322221378998810553344566777777777777777777777777777777777777777777777777776521399998721678887641366643046636776314323100137663066999987465037877468999998751661199999999999986146567777767777889 : Jnet Rel
 : 1---------11--------21--------31--------41--------51--------61--------71--------81--------91--------101-------111-------121-------131-------141-------151-------161-------171-------181-------191-------201-------211-------221-------231-------241-------251-------261-------271-------281-------291-------301-------311-------321-------331-------341-------351-------361-------371-------381-------391-------401-------411-------421-------431-------441-------451--- :

OrigSeq : MGLISRLFSATWLLLFSFQIAFTFGLPQLSTIGLDPIQQETCPTINEELVSTLNGQLQCCKKCPAGEGMLQLCTNTTQTVCRPCQEGSEFAVEASATAKCMQCKQCQELHPFAKFRKHCTPTSDAVCECVAGYFFIEVHSTCQGCTKCPPGYGAEKPCSWNEDSICKPCPEGTWSSTESATQKCLTCRKCKPGQIEMRPCTATQNTLCCPLYNPNCDENYVEDEFDDSKSLTGVGQLPENSFPPQFTYGDDYPMITIYCSLLGLVILTLLIYVFYKLWQQKLSMEDAKSIDAGMFHPAFTGIKASGGKSKSSKSSVSVKDSNDRQHLLGETQQYSYDPQKSGIPDQLLLELSQGLAVDNRWKHVGTKLGFNEESLQTFEKPKISEESGDAISVVKRMLTSWYSSRICTDPTPLNSLLVVLESSHGTSKLARHLKEYLKSSCAINSSTNSCPISYNQ : OrigSeq

Jnet : ------HHHHHHHHHHHHHHHHHH-------------------------------------------EE-------------------E--------------------------------------------------------------------------------------------------------EEEE---------EE-----------------------------------------------EEEEHHHHHHHHHHHHHHHHHHH---------------------------------------------------------------HHHHHHHHH---------HHHHHHHH----HHHHHHHHH--H--------HHHHHHHHHHH-------HHHHHHHHHHH---HHHHHHHHHHHHHHHH----------------- : Jnet

jhmm : --------------------------------------------------------------------------------------------------------------------------------------------------------------------------------------------------EE----------EE----------------------------------------------EEEEHHHHHHHHHHHHHHHHHHH------------------------------------------------------------------HHHHHH----------HHHHHHHH----HHHHHHHHHHHHHHH-----HHHHHHHHHHH-------HHHHHHHHHHH----HHHHHHHHHHHHHHH----------------- : jhmm

jpssm : -----HHHHHHHHHHHHHHHHHHHHHHHHHH------------------------------------EEEE-----------------EEE---------EE----------HH-------------------------------------HHHHH-------------------------------------EEEEE--------EE------------------------------------------------EEEEEE-------HHHHHHHHH---------------------------------------------------------------HHHHHHHHH---------HHHHHHHH----HHHHHH-------------HHHHHHHHHHHHH------HHHHHHHHHH---HHHHHHHHHHHHHHHHH----------------- : jpssm

Lupas 14 : ------------------------------------------------------------------------------------------------------------------------------------------------------------------------------------------------------------------------------------------------------------------------------------------------------------------------------------------------------------------------------------------------------------------------------------------------------------------------ : Lupas 14

Lupas 21 : ------------------------------------------------------------------------------------------------------------------------------------------------------------------------------------------------------------------------------------------------------------------------------------------------------------------------------------------------------------------------------------------------------------------------------------------------------------------------ : Lupas 21

Lupas 28 : ------------------------------------------------------------------------------------------------------------------------------------------------------------------------------------------------------------------------------------------------------------------------------------------------------------------------------------------------------------------------------------------------------------------------------------------------------------------------ : Lupas 28

Jnet_25 : -BB-B-BBBBBBBBBBBBBBBBBBBBBBBBBBBB-BB---BB--B---B-------B-BB--B--B-BB---B-------B--B-----B----B----B--B-BB--B--B--B---BBBB-BBBBBBB--BBBBBBB--BB----B-----BB--B-B--B--B-BB--BBBBB-------B--B--B------B--B----B-BBB-B----B----B--------B--B-B-BB--BBBB-BBB--B-BBBBBBBBBBB-BB--BB-B-BB-BB---------------BB-B----B----B--B-B-B---B--------B------B-B-B---------BB-B---B-----BB-BB--B-B---BB--B----B------B--BBBBBBB-BB---BB-BB-BBB-BB-BB-B--BB-BB---B--BB--BB-B------B-B---- : Jnet_25

Jnet_5 : ------B-BB-B--------B-B-B-B--B-B---------------------------B--------------------B------------------B-----------------------------------------------------------------------------------------------------------B------------------------------------------------B-B-BB-------------------------------------------------------------------------------------B------------B--BB--B------------------------BB--BB---B----------B--BB--B----BB--B---B---B---B--------------- : Jnet_5

Jnet_0 : ------------------------------------------------------------------------------------------------------------------------------------------------------------------------------------------------------------------------------------------------------------------B-----------------------------------------------------------------------------------------------------------------------------------------B---------------B--B------------B---B----------------------- : Jnet_0

Jnet Rel : 999843011111111112221110023212225777776677777777777777777766677776311034677776642025777300236777776322012677764233036777777777777777654567777777777765211122157777777777777777777777777653567777317820367777633236777766777777777677777664123566767677766677771146201333322221378998810553344566777777777777777777777777777777777777777777777777776521399998721678887641366643046636776314323100137663066999987465037877468999998751661199999999999986146567777767777889 : Jnet Rel

: 1---------11--------21--------31--------41--------51--------61--------71--------81--------91--------101-------111-------121-------131-------141-------151-------161-------171-------181-------191-------201-------211-------221-------231-------241-------251-------261-------271-------281-------291-------301-------311-------321-------331-------341-------351-------361-------371-------381-------391-------401-------411-------421-------431-------441-------451--- :

> Macrostomum lignano

Jnet Rel : 992588743677776677776641466777667777631113677776667777631001367777667777777777777777777777776677777632134677767777776322234677775133366777776311123677535677775456777766414677765235677777632236777765456677766777776641466777677777777777777777777777777777777777777777777777777777650336777766777777777776677776421256777777764433567777777777777777777777777777777777777777777777777764565112999998624788875178888615775466755550478876247999999861588861899999987448711999999999311111112235677777777777771001110277640211079 : Jnet Rel
 : 1---------11--------21--------31--------41--------51--------61--------71--------81--------91--------101-------111-------121-------131-------141-------151-------161-------171-------181-------191-------201-------211-------221-------231-------241-------251-------261-------271-------281-------291-------301-------311-------321-------331-------341-------351-------361-------371-------381-------391-------401-------411-------421-------431-------441-------451-------461-------471-------481-------491-------501-------511 :

OrigSeq : MPRVRAALVKPADGQPQKCRPGTESVGSLCCRPCAKGYGMITKCYRDEATGKFHDTECVPCSSGQMRTFSAAVSALYNCEKCRPCNRPSERLVSECTAERDTICPCADGYYRLRGGDCAPCASCPIGQGLADPASCNGSRVADCAPCPAGSFSAPLAGLPNRICRLCATACPPGYQLLPETCQRPSGPVCRSEASGLLANATELSPGAVTADSVFRDLQQQQQQQPAEEDSFLRKQQKQPQSRPLSDWKELIDNGNPRPPINLARFNLTQVARTEEPVGRHSGGRNDMIPVYCAVLGAIIVSLIVWIVFKYFRSRTYSRKTQQHLPASNALIPPQSPMYPPPRSCQPAALLKRRSVDSGFGGSEALAASVSSSTRPLKEISDSRFQRLENILSNNPDPAAWKELATELGFSADDVQEFEAQADTLRQSPIRLMLRTWSQRPGATIGLFGRCLQKAGRKDALILFKELAERQHQQQHQQQHGAGRRQSAQPRQTGQQQQQQLAEEPSLQEMCQV : OrigSeq

Jnet : ---EEEEE------------------------------EE-----------------EE------------------------------------------------------------------------------------------------------------------------------------------------------------------------------------------------------------------------------------------------------------------------------------------------------------------------------------HHHHHHHHH--------HHHHHHH----HHHHHHHH-------HHHHHHHHHHH-------HHHHHHHHHH----HHHHHHHHHHHHHHHHHH-------------------HHHHHH-----HHHH--- : Jnet

jhmm : ---EEEEE----------------------------------------------------------------------------------------------------------------------------------------------------------------------------------------------------------------------------------------------------------------------------------------------------------------------------------------------------------------------------------------HHHHHHHH--------HHHHHHH----HHHHHHHH-------HHHHHHHHHHH-------HHHHHHHHHH-----HHHHHHHHH--------------------------HHHHHHHH----HHHHH-- : jhmm

jpssm : ---EEEEE------------------------------EEE---------------EEEE-----------------------------------------EEE--------------EEEE--------EE----------EEEE------------------------------------------EE-----------------------------------------------------------------------------------------E------------------------------------------------------------------------------------------------------HHHHHHHHHH--------HHHHHHH----HHHHHHHHH--------HHHHHHHHHH-----HHHHHHHHHHH---HHHHHHHHHHHHHHHHHHHH-----------------------------HHHH--- : jpssm

Lupas 14 : --------------------------------------------------------------------------------------------------------------------------------------------------------------------------------------------------------------------------------------------------------------------------------------------------------------------------------------------------------------------------------------------------------------------------------------------------------------------------------------------------------------------------------- : Lupas 14

Lupas 21 : --------------------------------------------------------------------------------------------------------------------------------------------------------------------------------------------------------------------------------------------------------------------------------------------------------------------------------------------------------------------------------------------------------------------------------------------------------------------------------------------------------------------------------- : Lupas 21

Lupas 28 : --------------------------------------------------------------------------------------------------------------------------------------------------------------------------------------------------------------------------------------------------------------------------------------------------------------------------------------------------------------------------------------------------------------------------------------------------------------------------------------------------------------------------------- : Lupas 28

Jnet_25 : ---BBBBBBBB----B--B----B-----BB--B--B-B-B--B------B-B--B-BB-B--B-BB-B-----BB--B--B---B-BBB--BBBBB---BBBBB-B---BBB-B-BB--BB--B-B--BBB-B-B-----B-B--B--BBBB----B--B--B--B---B--B--B----B---BB-BB------B-BBBB-BB-BBBBB---B--BB-----------BB--B-----BB-BB-BB--BB----B---BB---B-BB-B--B---B--B-BB-B-BB-BBB-BB--B-B-BBBBBBB-BB--------B-------------B--B------B--------------BB----B---B--B---B--B---BB--B--BB--------B-BBB--B-B----B--B---B------BBB-BB--B-----BBB-BBB-BB--B-B--BB--B---B---B--------------B--B----------B--------BB-- : Jnet_25

Jnet_5 : -----B-BB---------B-----------B--B-----------------------B--------------------B--------------------------------------B-------------------------B-------------------B------B--------------------------------B---------------------------------------------------------------------------------------------------------------------------------------------------------------------------------------B------------B--BB--B------B--B-----------B--BB----------B--BB-BB-------BB--B------------------------------------------------- : Jnet_5

Jnet_0 : -----B-------------------------------------------------------------------------------------------------------------------------------------------------------------------------------------------------------------------------------------------------------------------------------------------------------------------------------------------------------------------------------------------------------------------------------------------B-------------B---B-------B----------------------------------------------------- : Jnet_0

Jnet Rel : 992588743677776677776641466777667777631113677776667777631001367777667777777777777777777777776677777632134677767777776322234677775133366777776311123677535677775456777766414677765235677777632236777765456677766777776641466777677777777777777777777777777777777777777777777777777777650336777766777777777776677776421256777777764433567777777777777777777777777777777777777777777777777764565112999998624788875178888615775466755550478876247999999861588861899999987448711999999999311111112235677777777777771001110277640211079 : Jnet Rel

: 1---------11--------21--------31--------41--------51--------61--------71--------81--------91--------101-------111-------121-------131-------141-------151-------161-------171-------181-------191-------201-------211-------221-------231-------241-------251-------261-------271-------281-------291-------301-------311-------321-------331-------341-------351-------361-------371-------381-------391-------401-------411-------421-------431-------441-------451-------461-------471-------481-------491-------501-------511 :

> Opisthorchis viverrini

Jnet Rel : 999876113100012035677777777777777777777666777763100034777766667777631001367777226677776310134677776311215776331356777777432203333304677610121256777777777777777777777777777777777777777777777777777777777776503367777632210357877202036777766777777777777777777777777777777777777777777777777777777766666666777777777777777777777753022889999999999999999987230764211001267873453077775211102367777777776321111110244211787764133231211257777766201000002034246555677613237888860367776667777762039999999999999999920120578877408999999999860687002234999875078432688887311357777522567777777889 : Jnet Rel
 : 1---------11--------21--------31--------41--------51--------61--------71--------81--------91--------101-------111-------121-------131-------141-------151-------161-------171-------181-------191-------201-------211-------221-------231-------241-------251-------261-------271-------281-------291-------301-------311-------321-------331-------341-------351-------361-------371-------381-------391-------401-------411-------421-------431-------441-------451-------461-------471-------481-------491-------501-------511-------521-------531-------541-------551-------561-------571--- :

OrigSeq : MNFTKSAFVLSCLMVLAYSSPVNNHSSRESLNGTEGAPKNVQHVNTGNKTVVTEFSEQHLETCPGPMQEFVSPVRGSPRCCRMCGPGTGMLRLCTDTDDTQCIGCEPGVEFSPTTSATLKCQQCRRCQDIHPLATTRIVCTPTTDTECGCMKGYYMSVNNQTCKACTVCKPNEGVIKSCEWNADTQCQACPAGFWSASVGDTVKCIPCKTCSENEVVVRTCRENEDALCCPKTNVNCTLSPIFGFAPPRIENETKTVNLTTLTPLETQQPADDIWMDGWPKKLTINGSSFYPPVQTAFHIDYTRYEQNGEASDTSSKQNQMLPIYCSIMGFIIVFLLLYVVYKLWKQREAMTNAKLCEVYTSSGYSTVKLPVNSTQMDSVLNGPCGGDGGGAADHVKSEHISRLTTKHLCGTNRQQERDPLIANFETGASELSYLEIQLGTLQRDVLGMICFQLSRSGWREMATNMDIPITSLLGPSANDSEFATQLAQAAQEAKHLILKESTTQNSPDEDTVKASARLLAKLCQQPTANVRVLLTELERINRSDIIAFISDQLTKVTPSPAIPISSWSASKDPRV : OrigSeq

Jnet : ----------HH-------------------------------------EE------------------EE------------------------------------------------------------------------------------------------------------------------------------------------EEEE-------EEE-----------------------------------------------------------------------------------------------HHHHHHHHHHHHHHHHHHHHHHHHHH------HHEE------EEEE--------------------------------HHHHH--------------------------EEEHHHEHHHHHHHHHHHHHH----HHHHHHH---------------HHHHHHHHHHHHHHHHHHHHH-----------HHHHHHHHHHHH-----HHHHHHHHHHHH-----HHHHHHHH---------------------- : Jnet

jhmm : ----------EE------------------------------------------------------------------EE----------------------------------------------------------------------------------------------------------------------------------------------------------------------------------------------------------------------------------------------------EE-HHHHHHHHHHHHHHHHHHHHHHHH-----EEEEE-----EEEE--------------------------------HHHHHH------------------------EHHHHHHHHHHHHHHHHHHHHH----HHHHHH------------------HHHHHHHHHHHHHHHHH-------------HHHHHHHHHHHH------EEEEHHHHHHH-----HHHHHH------------------------ : jhmm

jpssm : --------HHHHHHH---------------------------------EEEEE---------------EEEE----------------EEEE--------EEE------E-----------EE--HHHHH----------------------------------------------------------------------------E--------EEEEE------EEE----------------------------------------------------------------------------------------------HHHHHHHHHHHHHHHHHHHHHHHHHH-----HHHHHH------EEE-------HHHHH--------------HHHHHHHHHHH---------HHHH-HH------------EEEEEEEHHHHHHHHHHHHHHH--HHHHHHH---------------HHHHHHHHHHHHHHHHHHHHHHH--------HHHHHHHHHHHHH-----HHHHHHHHHHH-----HHHHHHHHHHH-------------------- : jpssm

Lupas 14 : -----------------------------------------------------------------------------------------------------------------------------------------------------------------------------------------------------------------------------------------------------------------------------------------------------------------------------------------------------------------------------------------------------------------------------------------------cccccccccccccc----------------------------------------------------------------------------------------------------------------------------------- : Lupas 14

Lupas 21 : ------------------------------------------------------------------------------------------------------------------------------------------------------------------------------------------------------------------------------------------------------------------------------------------------------------------------------------------------------------------------------------------------------------------------------------------------------------------------------------------------------------------------------------------------------------------------------------------------ : Lupas 21

Lupas 28 : ------------------------------------------------------------------------------------------------------------------------------------------------------------------------------------------------------------------------------------------------------------------------------------------------------------------------------------------------------------------------------------------------------------------------------------------------------------------------------------------------------------------------------------------------------------------------------------------------ : Lupas 28

Jnet_25 : -----BB-BBBBBBBBBBBBBBB--BB-BBBBBB-BBB--B--B--BBBBBBBBBB---B-BB-B----BBB-------BB--B--B-BBB--B-----BBB--B--B-BBB-----BB-BB-BBBBBBBB-B-BBBB-B-BBBBBBBBBB--BBBB--B-BBBB--BBBB-BBBB-BB----B-B---B--BBBBB-------B--B--B--B--BB--B---BBBBBBB---B-BBBBBBBBBBBB-B-----BB-B--B--B-B---BB-BBB--BB--B-B-----BBB-B-BBBBB-BB-B-----B--BB----BBBBBB-BBB-BBBB-BBBBBB-BB--------B-BB-BB---BB-BB-BBB----B--BB-BBBBB--------B----B--BB--BBB----------BBB-B--B---B-BB-B-BB-B---BBBBBBBBB---BB--BB--B-B-BB-BBB-------BB--BB-BB--B-BBB--------------B-BBB-BBB-BB--B-B-B-BBB--B--B----BBBBB---B--B-----B-B--B-------- : Jnet_25

Jnet_5 : ---------B-B-BB-B------------BB---------B--B-----BBB----------B-----------------B--------------------B------------------B-------------------------------------------------------------------------B---------B---------------------B-B-------B----------------------------------------------------------------------------------------B--BB-BBB---------B-----------B-----------B--------------------------------B-------------------B-------------B----------BB-BBB--B-----------B---B----------------B----------B------------------B-BB---B------B--BB--B-------BB--B------------B-B----------- : Jnet_5

Jnet_0 : -------------------------------------------------------------------------------------------------------------------------------------------------------------------------------------------------------------------------------------------------------------------------------------------------------------------------------------B---B-------------------------------------B-------------------------------------------------------------------------------B-BB-------------------------------------------------------------------------------B--B---------------B-------------------------- : Jnet_0

Jnet Rel : 999876113100012035677777777777777777777666777763100034777766667777631001367777226677776310134677776311215776331356777777432203333304677610121256777777777777777777777777777777777777777777777777777777777776503367777632210357877202036777766777777777777777777777777777777777777777777777777777777766666666777777777777777777777753022889999999999999999987230764211001267873453077775211102367777777776321111110244211787764133231211257777766201000002034246555677613237888860367776667777762039999999999999999920120578877408999999999860687002234999875078432688887311357777522567777777889 : Jnet Rel

: 1---------11--------21--------31--------41--------51--------61--------71--------81--------91--------101-------111-------121-------131-------141-------151-------161-------171-------181-------191-------201-------211-------221-------231-------241-------251-------261-------271-------281-------291-------301-------311-------321-------331-------341-------351-------361-------371-------381-------391-------401-------411-------421-------431-------441-------451-------461-------471-------481-------491-------501-------511-------521-------531-------541-------551-------561-------571--- :

> Schistosoma haematobium

Jnet Rel : 8413421789999993112001111111111202367787777651222314677777777765455133667776777776413367774311346777666667777631013467777764123467776676677777531134677776641046777513336777766777777777777767777751222315777776533357632236677767650336777761572026787721641677776677777777777777777777777677777763111677776654567777777777777777777777777777776512117777677777777777777656777612788887514787603210111677765455420111101003999871335677776407999999999870365577764167888775663789999998546844339999942123577751210177777641113999999853187378999999875077432888774157579 : Jnet Rel
 : 1---------11--------21--------31--------41--------51--------61--------71--------81--------91--------101-------111-------121-------131-------141-------151-------161-------171-------181-------191-------201-------211-------221-------231-------241-------251-------261-------271-------281-------291-------301-------311-------321-------331-------341-------351-------361-------371-------381-------391-------401-------411-------421-------431-------441-------451-------461-------471-------481-------491-------501-------511-------521-------531-------541-------551 :

OrigSeq : MAYMYNFKVFIEFLCLTIVWNSVIAVPLIFQSETLEGKTYPTVDTIAQMNNSNYTISSNENNETVTTTVSSIEGTGGVVEIQTETCDDPLKEFVSPVRGIPRCCRKCEPGNGMLRLCSNAEDTQCRPCKPGFEFSPFRSATKKCLHCRRCEEIHPLAKTRNECTPITDTICQCEKPYYMSEKEQTCKPCTVCKPGEGIVQACGWNSDTQCQSCPAGFWSAQSIDNVKCIPCQSCGKDQVLVKQCSPTSDTLCCPLNNPNCTHELSMYFDYSAYDQESDISDNNNKSNQMLPIYCSIMGLIIISLLCYVVYKLWRQREASKNAKLTDSYNSNKTDLLDRTSCLDNNHLQHRRISSGFINNNNNAHLPNHSPQSTTELNNTINSTPDNDINNHLQFNDIIVGHEKAPLLGKLDYSNSSFSNFEQKPISVIPMNILGVICYRLSQHGWQELANIMDLETSKFDQLPSEVTSDLLSAAMEAQNTVESHLKVCNQDNSNTIQSITHNNPKNNLTMTVSMFQYMCLQNTVNLGQLMNSLQKLNRSDLVALIQQHTGIIK : OrigSeq

Jnet : -------HHHHHHHHHH----HHHHHHHHHHHH------------------------------------------------------------------------------------------------------------------------------------------------------------------------------------------------------------EEEE--------EEEE----------------------------------------E--------------------------------------------EEE---------------------------HHHHHHHHHH-----HHHHHHH------------HHHHHH-H-HHHHHHH-----------HHHHHHHHHHHH----HHHHHHH-----------HHHHHHHHHHHHHHHHHHHHHHHHH-------EEEE--------EEHHHHHHHHHHH---HHHHHHHHHHHHH----HHHHHHH------ : Jnet

jhmm : -------HHHHHHHH------------------------------------------------------------------------------------------------------------------------------------------------------------------------------------------------------------------------------EEE----------EEE----------------------------------------EE-------------------------------------------EEEE--------------------------HHHHHHHHHH-----HHHHHHHH---------------------HHHHH------------HHHHHHHHHHHH----HHHHHHH-----------HHHHHHHHHHHHH----HHHHHEEE-------EEEEE-------EEEEHHHHHHHHHH--HHHHHHHHHHHHH----HHHHHHH------ : jhmm

jpssm : -------HHHHHHHHHHH--HHHHHHHHHHHHHH------------HHHH------------------E---------------E------EEEE----------------EEEE----------EE----------------EEEE------------------EE-----------------------------EEEE----------------EE-----------E--------EEEE-------EEE----------------------------------------E----------------------------------------------------------------------------HHHHHHHHH-------HHH-------------HHHHHHHHHHHHHHHHHH---------HHHHHHHHHHHHHH---HHHHHH------------HHHHHHHHHH-HHHHHHHHHHHHHHH------------------HHHHHHHHHHHH-----HHHHHHHHHHH-----HHHHHHH------ : jpssm

Lupas 14 : ---------------------------------------------------------------------------------------------------------------------------------------------------------------------------------------------------------------------------------------------------------------------------------------------------------------------------------------------------------------------------------------------------------------------------------------------------------------------------------------------------------------------------------------------CCCCCCCCCCCCCC-------------- : Lupas 14

Lupas 21 : ------------------------------------------------------------------------------------------------------------------------------------------------------------------------------------------------------------------------------------------------------------------------------------------------------------------------------------------------------------------------------------------------------------------------------------------------------------------------------------------------------------------------------------------------------------------------- : Lupas 21

Lupas 28 : ------------------------------------------------------------------------------------------------------------------------------------------------------------------------------------------------------------------------------------------------------------------------------------------------------------------------------------------------------------------------------------------------------------------------------------------------------------------------------------------------------------------------------------------------------------------------- : Lupas 28

Jnet_25 : --B-B-B-BBB-BBBB-B-B-BB-BBBBBBBBBBB---BBB-B-BB-BBB-B-BBBBB---B-BBBBBBBBB-BB-BBB-B----B--B---B-B---B---BB--B--B-B-B--B--B--B-B-BB-----BB-B--BB--BB-B--B--BB-BBBBBB-BBBB-BBBB------BBB------B-B--B--B-BBB-BB----B--B--B--BBBBB--B----B--B--B-----BB--B---BBBBBB-B----B--B--BBBBB-BB-B-B-BB---------BBBBBB-BBB-BBBBBBB--B-B-----------B-----B---------BBBB-------------------B-B---B--BB--B-------BB--B---B-B----B------BB--B-BB-BBB--B-----B---B-BBBBBBBBB----B--BB-BB------B------BB--BB-BB--B--BB--BB--B-------B--B--------B-BBBBBB-BBBB--BB-BB-BB--B--B----BBBBB-----B-- : Jnet_25

Jnet_5 : ---------B--B--------BB------B----B----------B--B----B--B-------B--------------------B------B----------B--------------------B------------------B-------------------------------------------------------------------------B---------B----------------------BB----------------------------------------BBBB-------------------------------------------------------------------------------------------B------------------------B--BB--------------BB-BBB-BB-------BB--B-------------B---BB---------B---B----------------------B-B-B-BB---B------B--B---B-------BB-BB-------- : Jnet_5

Jnet_0 : -----------------------------B--------------------------------------------------------------------------------------------------------------------------------------------------------------------------------------------------------------------------------------------------------------------------------B------------------------------------------------------------------------------------------------------------------------------------B--------------------------------------------------------------------------------------------------------------------- : Jnet_0

Jnet Rel : 8413421789999993112001111111111202367787777651222314677777777765455133667776777776413367774311346777666667777631013467777764123467776676677777531134677776641046777513336777766777777777777767777751222315777776533357632236677767650336777761572026787721641677776677777777777777777777777677777763111677776654567777777777777777777777777777776512117777677777777777777656777612788887514787603210111677765455420111101003999871335677776407999999999870365577764167888775663789999998546844339999942123577751210177777641113999999853187378999999875077432888774157579 : Jnet Rel

: 1---------11--------21--------31--------41--------51--------61--------71--------81--------91--------101-------111-------121-------131-------141-------151-------161-------171-------181-------191-------201-------211-------221-------231-------241-------251-------261-------271-------281-------291-------301-------311-------321-------331-------341-------351-------361-------371-------381-------391-------401-------411-------421-------431-------441-------451-------461-------471-------481-------491-------501-------511-------521-------531-------541-------551 :

> Schistosoma curassoni
 586 - 18AA = 568 (from 2^nd^ Met – found an “O” before)

Jnet Rel : 7228715886452111689999998621283321204765312340010111111101157777777654567777632134677766666777763177126777764311346775333567777776545677777775133157742304677776777777777777777777777533115677776545677777777777777765224677776303202678772021215777676777777777777777777777767777764123467776301125677777776665555667777777777777777722777777777777652356777777776421011016777764222345772261002567112344306651322231467787762189999999987035788887442033210276430139999743312129999999923111211221017777554112499999985318737899999987517843256665502122157787776511031123577777742278 : Jnet Rel
 : 1---------11--------21--------31--------41--------51--------61--------71--------81--------91--------101-------111-------121-------131-------141-------151-------161-------171-------181-------191-------201-------211-------221-------231-------241-------251-------261-------271-------281-------291-------301-------311-------321-------331-------341-------351-------361-------371-------381-------391-------401-------411-------421-------431-------441-------451-------461-------471-------481-------491-------501-------511-------521-------531-------541-------551-------561----- :

OrigSeq : MTNIVHSTHLLAIEILSLQLHLILIFYDHLNEISITQSKLIGLSTLENLLSIKIHSSWITNTSVFTFTIETCDDPLKEFVSPVRGIPRCCRKCEPGTGMLRLCSNAEDTQCRPCKPGFEFSPFRSATKKCLHCRRCEEIHPLAKTRNECTPITDTICQCEKPYYMSEKEQTCKPCTVCKPGEGIVQACGWNSDTQCQSCPAGFWSAQSIDNVKCIPCQSCGKDQVLVKQCSPTSDTLCCPLNNPNCTHELSMYFDYSAYDQESDISDNNNKSNQMLPIYCSIMGLIIISLLCYVVYKLWRQREASKNAKLTDSYNSNKTDLLDRTSCLDNNHLQHRRISSGFINNNNNAHLPNHSPQSTTELNNTINSTPDNDINNHLQFNDIIVGHEKAPLLGKLDHSNSSFSNFEQKPISVIPMNILGVICYRLSQHGWQELANIMDLETSKFDQLPSEVTSDLLSAAMEAQNTVESHLKLCNQDNSNTIQSITNNNPKSNLTMTVSMFQYMCLQNTVNLGQLMNSLQKLNRSDLVALIQQHTGIIKSKKSTNHSNEEYKDKTKSMKSKENFQIEN : OrigSeq

Jnet : --EEE--------HH-HHHHHHHHHHH-------------------EEHHHHHHHHH----------------------------------------EEEE---------------------------------------------------------------------------------------------------------------------------EEE--------EEE-------------------------------------------------------------------------------------------------------------------------HHH--------------------EE-----HHHHHH--------------------HHHHHHHHHHHH---HHHHHHHHHHHHHH------HHHHHHHHHHHHHHHHHHHHHHHHHHHHHHHHHHH---------HHHHHHHHHHHH---HHHHHHHHHHHHH----HHHHHHHH--EEE------------HHH-------------- : Jnet

jhmm : ---EE-------HHHHHHHHHHHHHHHHH-HHH-------------EEE-------------------------------------------------EE----------------------------------------------------------------------------------------------------------------------------------------------------------------------------------------------------------------------------------EE-------------------------------HHHH---------------EE-EEE----HHHHHHHH--------------------HHHHHHHHHHH---HHHHHHHHHHHHHHHH-------HHHHHHHHH---HHHHHHHH----HHHHHHHHH--------EEEHHHHHHHHHH--HHHHHHHHHHHHH----HHHHHHH---EEE------------HHH-------------- : jhmm

jpssm : -EEEEE----------HHHHHHHHHHH-------E-----------HHHHHHHHHHHH--------------------EEE----------------EEEE--------EEEE------------------------------EE-----EE------------------------------------------------------------------------EEEE-------EEEE---------------------------------------EE----------------------------------------------------------------------------HHHH----------------------------------------HHHHH----------HHHHHHHHHHHHH--HHHHHHH------------HHHHHHHHHH--HHHHHHHHHHHHHHHHH---------------HHHHHHHHHHHH-----HHHHHHHHHHH-----HHHHHHHHHHEEE------------HHH----------E--- : jpssm

Lupas 14 : -------------------------------------------------------------------------------------------------------------------------------------------------------------------------------------------------------------------------------------------------------------------------------------------------------------------------------------------------------------------------------------------------------------------------------------------------------------------------------------------------------------------------------CCCCCCCCCCCCCC------------------------------------------- : Lupas 14

Lupas 21 : ---------------------------------------------------------------------------------------------------------------------------------------------------------------------------------------------------------------------------------------------------------------------------------------------------------------------------------------------------------------------------------------------------------------------------------------------------------------------------------------------------------------------------------------------------------------------------------------- : Lupas 21

Lupas 28 : ---------------------------------------------------------------------------------------------------------------------------------------------------------------------------------------------------------------------------------------------------------------------------------------------------------------------------------------------------------------------------------------------------------------------------------------------------------------------------------------------------------------------------------------------------------------------------------------- : Lupas 28

Jnet_25 : --BBBB-BBB--B-BBB-BBBBBBBBB--B--BBB---BBBBBB-BBBBBBBBBBBBBBBBBBBBBB-BB-B-B---BB-B-------BB--B-BB-BBB--B-------B--B--BB-BB-B---B--B-BB---B-B--BBBB---B--B-BBBB-BB---BBB--B---B----B--B--BB--B-----B-B--B---BBBB--B----B--B--B-----BB--B---BBBBBB-B----B--B--BBBBBBB----B-BB---B----BBB-BBBBB--BBBB-BB--BB--B-------B--BB-B----BBB-B-BB-BBB---------B--B--------B------BB--B---B--B----B---B--B---B------BB--B-B----B--B----BB-----BBBBBBBBB----B--B--BB--BB--B--B---BB--BB-BB------B---B--B---B--BB--B--------B-BBBBBB-BBBB--BB-BB-BB--B--B----BBBBB--BB-BB-----B------B---B--B-----B-B-- : Jnet_25

Jnet_5 : ---BB---------B----B-BBB-----B----B----B-----B---BB--B-BBBB---B-----B--B----------------B----------B----------B----------------------------------------------------B-------------------------------------------------B----------------------B----------------B-------------------------B--B---B--------------------------------------------------------------------------------------------------------B-------------------------BBB-BB----------B---B------------------B--B---------------------------------B---B-BB---B------B--B---B-------BB-BB------------------------------------- : Jnet_5

Jnet_0 : -------------------B-----------------------------------------------------------------------------------------------------------------------------------------------------------------------------------------------------------------------------------------------------------------------------------------------------------------------------------------------------------------------------------------------------------------B-------------------------------------------------------------------------------------------------------------------------------------------------- : Jnet_0

Jnet Rel : 7228715886452111689999998621283321204765312340010111111101157777777654567777632134677766666777763177126777764311346775333567777776545677777775133157742304677776777777777777777777777533115677776545677777777777777765224677776303202678772021215777676777777777777777777777767777764123467776301125677777776665555667777777777777777722777777777777652356777777776421011016777764222345772261002567112344306651322231467787762189999999987035788887442033210276430139999743312129999999923111211221017777554112499999985318737899999987517843256665502122157787776511031123577777742278 : Jnet Rel

: 1---------11--------21--------31--------41--------51--------61--------71--------81--------91--------101-------111-------121-------131-------141-------151-------161-------171-------181-------191-------201-------211-------221-------231-------241-------251-------261-------271-------281-------291-------301-------311-------321-------331-------341-------351-------361-------371-------381-------391-------401-------411-------421-------431-------441-------451-------461-------471-------481-------491-------501-------511-------521-------531-------541-------551-------561----- :

> Schistosoma japonicum

Jnet Rel : 9601113403217886431052122220214432134653356666655456777767777766414667776777777777777777777777654567653567775167136777653567777634612467777643113467765225677777777777654211123332112577775125677777777777777777777766421135677765225677765456677777653212577751221127787721643677776677777777777777777777777777777776553100112111234677432111220335544223235677777777777777102765021146777766666667777777777776534567655415783302322156524643333025562111456676654566611137899999998633663677750367887777652789999999999822351112467777776321414687776512111399999985318737899999987437742588877642303325776400026899 : Jnet Rel
 : 1---------11--------21--------31--------41--------51--------61--------71--------81--------91--------101-------111-------121-------131-------141-------151-------161-------171-------181-------191-------201-------211-------221-------231-------241-------251-------261-------271-------281-------291-------301-------311-------321-------331-------341-------351-------361-------371-------381-------391-------401-------411-------421-------431-------441-------451-------461-------471-------481-------491-------501-------511-------521-------531-------541-------551-------561-------571-------581-------591----- :

OrigSeq : MTRVYQIIHFFNINNKQTSTCVYTFSIFIELLYFIIIWNSVLAGPFIFQIEHMEKKNDELVNITSQLLENNKTTGRNNYNETETTTPGTTDGTGDVVEIQTETCNDPYEEFVSPVHGTPRCCRKCEPGTGMLRLCSNKEDTQCRPCKPGFEFSPIRSATKKCLQCRRCEELHPLAKTRTECTPITDTICQCEKLYYMSEKEQTCKPCTVCQPGEGIVKACSWNSDTQCQSCPAGFWSAQTIDNVKCIPCQSCGKDQVLVRQCSSISDTLCCPLNNPNCTHELSSVFHLDDYSTYDQDGDIPENGNKSNQMLPIYCSIMGLIIISLLCYVVYKLWRQREASKNAKLTDSYNITIGCNKTDLLDRTSCLDNHHLQQQRISPGFVNTAITNINNNTNVSNHSPQSISEIDKIVSVTSDNNNNNQYNDLTVGHEKAPLLGKLQRLSVCYTNQKNYEQPVTGIPLNILGVICYRLSQNGWRELASTMNLDTSQFNQLPLEVTSDLLSAAMEAQTNAESQLKSSHPNNSNTLQQTKTDNNHENDLRMTVSMFRYICIQNTVNLGQLINYLHKINRSDLVGLLQQQQTGTMKTKKSSEEHKNKTK : OrigSeq

Jnet : ---HEEEEEEEE------EEEE------HHHHHH---------------------------------------------------------------------------EEEE----------------EEE----------------------------------------------------------------------------------------------------------------------------EEE--------EEE--------------------------------------------HHHHHHHH-------------------------------------------------EE-----------------------------HHHHHHHHH-----HHHHHH----------------------------------EHHHHHHHHHHHHHH----HHHHHH------------HHHHHHHHHHHHHH------------------EE-----------HHHHHHHHHHHHH---HHHHHHHHHHHH-----HHHHHHHH--EEEE-----HH------ : Jnet

jhmm : ---EEEEEEEEE------EEE---------HHH----------------------------------------------------------------------------EEE-----------------EE-----------------------------------------------------------------------------------------------------------------------------------------EE-------------------------------------------------EEE------------------------------------------EEE---EEE-----------------------------HHHHHHHHH---HHHHHHHH----------------HHHH-------------EEEHHHHHHHHHHHHH----HHHHH-------------HHHHHHHHHHHHH--------------------E-----------EEEEHHHHHHHHHH--HHHHHHHHHHHH-----HHHHHHHH--EEEE------------- : jhmm

jpssm : --HHHHHHEEE-------EEEEEEHHHHHHHHHHHE-------------------------------------------------------------------------EEEE----------------EEEE--------EEEE-------------------------HHHHHHH---------------------------------------------------------------------EE-------EEEEE-------EEE-------------------------------------------HHHHHHHHHH-------HHHHHH------------------------------------------------------------------HHHHHHHHH------------------EEEE------------------------HHHHHHHHHHHHHH----HHHHHH------------HHHHHHHHHHHHHHH----------------EEEE---------HHHHHHHHHHHHH-----HHHHHHHHHHH-----HHHHHHHHHH-HH------HH------ : jpssm

Lupas 14 : ---------------------------------------------------------------------------------------------------------------------------------------------------------------------------------------------------------------------------------------------------------------------------------------------------------------------------------------------------------------------------------------------------------------------------------------------------------------------------------------------------------------------------------------------------------------------------------------------------------------------- : Lupas 14

Lupas 21 : ---------------------------------------------------------------------------------------------------------------------------------------------------------------------------------------------------------------------------------------------------------------------------------------------------------------------------------------------------------------------------------------------------------------------------------------------------------------------------------------------------------------------------------------------------------------------------------------------------------------------- : Lupas 21

Lupas 28 : ---------------------------------------------------------------------------------------------------------------------------------------------------------------------------------------------------------------------------------------------------------------------------------------------------------------------------------------------------------------------------------------------------------------------------------------------------------------------------------------------------------------------------------------------------------------------------------------------------------------------- : Lupas 28

Jnet_25 : ----B-BBBBB-B----B--B-B-B-BBB-BBBBBBBB-BBBBBBB-BBBB-B---B--B--B-B-B-B---BBBBB-B--B--BBBBBBB--B-B--B-B-BB-B--BBBBB---B--BBB--B--B-BBB--B-----B-BBBB--BBBBB-B---B--BBBB-B---B--B--BB--BBBBBBBBBBBB-BBBBB--B-BBBBB--B----BBB-BBBBB-B-BBB-B--BBBBB-------B--B--B----BB---B---BBBBBB-B--B-BB-BBB-BBBB--B--BBB-B--B--B---BBBBBBBBBBBBBBBBBBBBBBBB-BB-----B--B-BB-B----B---B--B---BBBBB-BBB----B---BB---B-------BBB-B-------B--BB-BB----B--BB--B-B----B-BBB----B-BBB--B-----BB-BBBB-BBBBBBBBB----B--BB-BB-B-B--B--BB--BB--BB-BB--B---B---B--B---B--BB------------B-BBBBBB-BBBB--BBBBB-BB-BB--B----BBBBB------BB-------------- : Jnet_25

Jnet_5 : -------B------------B----------BB-BB------B--B-B-B---------B------B-----------------B------------------B-----------------B--B------------------------------------B-------------------------------------------------------------------------B---------B-----------------------B--------------------------------------------B-BB--BB-------B---B--------B----------------------B---------------------------------------B---B--------------B------------------------------------BB-BBB--B-------BB--B-------------B---BB-------------------------------------B-B-B-BB--BB------B--B---B-------B--BB---------------------- : Jnet_5

Jnet_0 : ---------------------------------------------------------------------------------------------------------------------------------------------------------------------------------------------------------------------------------------------------------------------------------------------------------------------------------------------------------------------------------------------------------------------B----------------------------------------------------------BBB----------------------------------------------------------------------------------B------------------------------------------------ : Jnet_0

Jnet Rel : 9601113403217886431052122220214432134653356666655456777767777766414667776777777777777777777777654567653567775167136777653567777634612467777643113467765225677777777777654211123332112577775125677777777777777777777766421135677765225677765456677777653212577751221127787721643677776677777777777777777777777777777776553100112111234677432111220335544223235677777777777777102765021146777766666667777777777776534567655415783302322156524643333025562111456676654566611137899999998633663677750367887777652789999999999822351112467777776321414687776512111399999985318737899999987437742588877642303325776400026899 : Jnet Rel

: 1---------11--------21--------31--------41--------51--------61--------71--------81--------91--------101-------111-------121-------131-------141-------151-------161-------171-------181-------191-------201-------211-------221-------231-------241-------251-------261-------271-------281-------291-------301-------311-------321-------331-------341-------351-------361-------371-------381-------391-------401-------411-------421-------431-------441-------451-------461-------471-------481-------491-------501-------511-------521-------531-------541-------551-------561-------571-------581-------591----- :

> Hydatigera taeniaeformis

Jnet Rel : 998020111111101157777645667776777777666777763101347776511467777631103467777753100356753566777777652267877502000001357888731013467776677777777765357775311346777641213477743121467777663333377731883037787720203677776677777667777640123677776677777666777763200367765565555523225777676666666677777777777777777777777777777777777777777777777777652029999982236888774027887633664167611236011122222116661279999997898703785689999998704885689998343777210567777777777767777764113667777889 : Jnet Rel
 : 1---------11--------21--------31--------41--------51--------61--------71--------81--------91--------101-------111-------121-------131-------141-------151-------161-------171-------181-------191-------201-------211-------221-------231-------241-------251-------261-------271-------281-------291-------301-------311-------321-------331-------341-------351-------361-------371-------381-------391-------401-------411-------421-------431-------441-------451-------461-------471- :

OrigSeq : MPILPIIWTLISFFRCSAMPQALQQPLNSTQLDPIRQETCPSPNEELVSTLNGPLRCCNKCPPGEGMLQLCTNQTQTVCRPCQEGSEFSLEASATAKCMQCKQCQELHPFAKVRKHCTPTSDAVCECVSGYFFIEAHSTCQSCTKCPPGQGAEKPCEWNKNSVCKPCAEGTWSSTDSATDRCQTCRKCKPGQIEMRPCTATQNTLCCPLHNPNCQDTFEEEEFDEPKFVQTQGKCTDDLAASQLAYGDDYPMITIYCSLLGLVILTLLIYVFYKLWQQRLSAEDVKTIELDVCYPAFTGIKPGGGKLRSSKLSSPIKDTTDRQHLLEGSQQYSYAPQHPTINDALLTELSQGLAVENRWKRVGGLLGFSEESLQNFEKMAASDELSNSVNSAAVATRLMLTSWYSARAATDPNPLMTLLMVLERTPSTEHLCRSLKACIKHSNASSNCAMSPAQPLSQPPTTTISTEGQKPSNH : OrigSeq

Jnet : -----HHHHHHHHH---------------------------------------------------EE------------E----------------------------HHHH-------------------------------------------------------------------------------EEEE--------EE--------------------------------------------------------------------------------------------------------------------------------------HHHHHHHHH----------HHHHHHH----HHHHH-----HHHHHHHHH----HHHHHHHHHHHHHHH----HHHHHHHHHHH----HHHHHHHHHHHHH----------------------------------- : Jnet

jhmm : ------------------------------------------------------------------------------------------------------------------------------------------------------------------------------------------------EE---------------------------------------------------------------------------------------------------------------------------------------------------HHHHHH-----------HHHHHHH-----HHH-----HHHHHHHHHHH---HHHHHHHHHHHHHH-----HHHHHHHHHH-----HHHHHH-HHHHH------------------------------------ : jhmm

jpssm : ----HHHHHHHHHHH------------------------------EEEE----------------EEEE--------EEEEE-------------------------HHHHHHHH-------EEEE------------------------EEEE--------EEE-----EEE----------EEE-----EEEEE-------EEE----------------------EE----------------------EEE--------------E---------------------------------------------------------------------HHHHHHHHHH---------HHHHHHH----HHHHHH------------------HHHHHHHHHHHHHH----HHHHHHHHHHH----HHHHHHHHHHHHHH-----------------------E---------- : jpssm

Lupas 14 : ------------------------------------------------------------------------------------------------------------------------------------------------------------------------------------------------------------------------------------------------------------------------------------------------------------------------------------------------------------------------------------------------------------------------------------------------------------------------------------------ : Lupas 14

Lupas 21 : ------------------------------------------------------------------------------------------------------------------------------------------------------------------------------------------------------------------------------------------------------------------------------------------------------------------------------------------------------------------------------------------------------------------------------------------------------------------------------------------ : Lupas 21

Lupas 28 : ------------------------------------------------------------------------------------------------------------------------------------------------------------------------------------------------------------------------------------------------------------------------------------------------------------------------------------------------------------------------------------------------------------------------------------------------------------------------------------------ : Lupas 28

Jnet_25 : ----BBB-BBBBBB-BBBB--BB-BBBBBB-B--B---BB----B-B-------B-BB--B--B-BB---B------BBBBB-----B----B-B--B--B---B--B--BBBB--B--B-B-B--BB---B-BB-B---B-B--B----BBBB-B---BBBBB--B--BBB-B-------B--B-----B-BBB--B----BBBB--B--B-B----B-B--B-B--B--B--B----BBB-BBB-----BBBB-BB-B-BBB-BBBB-BBB--B--B-----B--B-B--B--BBBBB---B--B-B--BBBBBB--B--B-------BB----------B--BB---B-------BBBBBB-B-B----B--B-----B--B------B--BBB-BB--BB---BB--B-BBB-BB--B----BB--B--BB--BB--B-BB--B-B-------------BB--------- : Jnet_25

Jnet_5 : ----------B--B-B--B--------------------------------------B--------------------B------------------B-------------------------------------------------------------------------B---------B-----------------------B-----------------------------------------------B--B--B-----------B-------------------------------------------------------------------------B------------B--BB--------------------------------B--BB--------------BB-BB-------B---B---B--------------------------------------- : Jnet_5

Jnet_0 : -------------------------------------------------------------------------------------------------------------------------------------------------------------------------------------------------------------------------------------------------------------------------------B----------------------------------------------------------------------------------------------------------------------------------------------B---B-----------B---B--------------------------------------- : Jnet_0

Jnet Rel : 998020111111101157777645667776777777666777763101347776511467777631103467777753100356753566777777652267877502000001357888731013467776677777777765357775311346777641213477743121467777663333377731883037787720203677776677777667777640123677776677777666777763200367765565555523225777676666666677777777777777777777777777777777777777777777777777652029999982236888774027887633664167611236011122222116661279999997898703785689999998704885689998343777210567777777777767777764113667777889 : Jnet Rel

: 1---------11--------21--------31--------41--------51--------61--------71--------81--------91--------101-------111-------121-------131-------141-------151-------161-------171-------181-------191-------201-------211-------221-------231-------241-------251-------261-------271-------281-------291-------301-------311-------321-------331-------341-------351-------361-------371-------381-------391-------401-------411-------421-------431-------441-------451-------461-------471- :

> Taenia solium

Jnet Rel : 961322100333333357777777777777777777777777777777777777776667777631111267777753101377732123677776643046777656666554246777750330577764033677777765356555411567777652015677640234677767765356777730321137787721643677776677777777777777777777777777777776777765021422776677201000011123453222221357777777777777777777777652106777766777777777777777764229999988516787776102320035664048871133677777777777752378999885311378771789999998616733029999999998621677777777777777777776545667777889 : Jnet Rel
 : 1---------11--------21--------31--------41--------51--------61--------71--------81--------91--------101-------111-------121-------131-------141-------151-------161-------171-------181-------191-------201-------211-------221-------231-------241-------251-------261-------271-------281-------291-------301-------311-------321-------331-------341-------351-------361-------371-------381-------391-------401-------411-------421-------431-------441-------451-------461-------471- :

OrigSeq : MSVLLLIWTLISPFWCSALPQALQQPLNSTQLDPIQQETCPSANEELVSTLDGPLRCCNKCPPGEGMLQLCTNQTQTVCRPCQEGSEFSLEASATAKCMQCKQCQELHPFAKFREHCTPTSDAVCECVSGYFFIEAHSTCQSCTKCPPGQGAEKPCGWNENTVCKPCAEGTWSSTDSATDICQTCRRCKPGQIEMRPCTATQNTLCCPLHNPNCEDTFEEEEFDEPKSMQSQGKCTDDQAASQLAYGDDYPMITIYCSLLGLVILTLLIYVFYKLWQQRLSAEDTKTIEADVCYPAFTGLKSGRDKLRSSKISAPSKDTTDRQHLLGGSQQYSYAPQNPTIHEALLTELSQGLAVEDRWKHVGGLLGFGEESLQNFEKVGASDETSNPVDSAAIATRLMLTSWYSARAATDPNPLTSLLMVLERTPSTGHLCRCLKEYIKQSSTSSPYAMSPTQPPSQLPTSAISPEAQNTSNH : OrigSeq

Jnet : --HHHHHH---------------------------------------------------------EEE-----------E---------------------------------------------------------------------------------------------------------------EEEE--------EEE------------------------------------------------------------EEEHHHH--------------------------------------EEE--------------------------HHHHHHHHH--------HHHHHH-------HHHHH------------------HHHHHHHHHHHH-----HHHHHHHHHHHH-----HHHHHHHHHHHHHH--------------------------------- : Jnet

jhmm : ------------------------------------------------------------------------------------------------------------------------------------------------------------------------------------------------------------EE--------------------------------------------------EE------EEEEEEEE---------------------------------------EEE---------------------------HHHHHHHH--------HHHHHH-------HHHH-------------------HHHHHHHHHH-------HHHHHHHHHHHH------HHHHHHHHHHHHH--------------------------------- : jhmm

jpssm : --HHHHHHHHHHHHH--------------------------------------------------EEEE--------EEEE-----EEE---------E------------------------EE--------E-------------------------------------EE------------------EEEEE-------EEE-----------------------------------------------E-------------HHHHHHHHH----HHHH----------------------------E--------------------------HHHHHHHHHH---------HHHHH------HHHHHHHH-----------------HHHHHHHHHHH------HHHHHHHHHHH---HHHHHHHHHHHHHHH---------------------------------- : jpssm

Lupas 14 : ------------------------------------------------------------------------------------------------------------------------------------------------------------------------------------------------------------------------------------------------------------------------------------------------------------------------------------------------------------------------------------------------------------------------------------------------------------------------------------------ : Lupas 14

Lupas 21 : ------------------------------------------------------------------------------------------------------------------------------------------------------------------------------------------------------------------------------------------------------------------------------------------------------------------------------------------------------------------------------------------------------------------------------------------------------------------------------------------ : Lupas 21

Lupas 28 : ------------------------------------------------------------------------------------------------------------------------------------------------------------------------------------------------------------------------------------------------------------------------------------------------------------------------------------------------------------------------------------------------------------------------------------------------------------------------------------------ : Lupas 28

Jnet_25 : --BB-BBBBBBBBBBB--BB-BB--BB-BB-B--B-B--B------B---B---B-BB--B--B-BB---B-------B-BB--B-BB----B-B--B--B--B--BB----B-----BB--BBBBB---BBBBB-B--B--BB---BB-BBBB-----B------B--BBBBB-------B--B--B-----BB--B---BBBBB--B----B--B---B-B-B---B--B--B----BBB-B-B--B--BBBBBBBBBBBBBB-BBBBBBB--B----------------B---B-B----------BBBBB-B--B-------------BB--B-------B--BB---------B--BB--B-B---BB--B----B-------------BBBBBBB-BB---BBBB--BB-BB--B------B--BB-BB--BB-------BBBB------------BB---B------ : Jnet_25

Jnet_5 : -----B---------B--B--------------------B-----------------B--B-----------------B------------------B-------------------------------------------------------------------------B---------------------------------B----------------------------------------------BB--BBBBB-----B--B-------------------------------------------B------------------------------B-------------B--BB--B----------------------------BB--BB-------------BB-BB--B---------B---B--------------------------------------- : Jnet_5

Jnet_0 : -----------------------------------------------------------------------------------------------------------------------------------------------------------------------------------------------------------------------------------------------------------------B--------------------------------------------------------------------------------------B-----------------B--------------------------------B--B--------------B---B-------------------------------------------------------- : Jnet_0

Jnet Rel : 961322100333333357777777777777777777777777777777777777776667777631111267777753101377732123677776643046777656666554246777750330577764033677777765356555411567777652015677640234677767765356777730321137787721643677776677777777777777777777777777777776777765021422776677201000011123453222221357777777777777777777777652106777766777777777777777764229999988516787776102320035664048871133677777777777752378999885311378771789999998616733029999999998621677777777777777777776545667777889 : Jnet Rel

: 1---------11--------21--------31--------41--------51--------61--------71--------81--------91--------101-------111-------121-------131-------141-------151-------161-------171-------181-------191-------201-------211-------221-------231-------241-------251-------261-------271-------281-------291-------301-------311-------321-------331-------341-------351-------361-------371-------381-------391-------401-------411-------421-------431-------441-------451-------461-------471- :

> Echinococcus multilocularis ISOFORM 1 - pathogen_EmW_scaffold_02.6656(.1)

Jnet Rel : 3024443211111001336777777777777777765456777764136677763111126777763176157773212367777764314664433112012346777777777777765356777632134677776513313323345677777776545667776776535677775167212677776332367777667777777777777777777777777777654567763212124677541221446899999998515777710112328999987517887046889999999986888753121287105788764278887677511577524666562000114157899 : Jnet Rel
 : 1---------11--------21--------31--------41--------51--------61--------71--------81--------91--------101-------111-------121-------131-------141-------151-------161-------171-------181-------191-------201-------211-------221-------231-------241-------251-------261-------271-------281-------291-------301-------311-------321-------331-------341-------351-------361---- :

OrigSeq : MLILLLILTLLSSFRWIQFNGGPEFMPETCPSANEELVSTLNGPLRCCNKCPSGEGMLQLCTNQTQTVCRPCQEGSEFSLEASATAKCMQCRQCQELHPFAKFRKHCTPTSDAVCECVSGYFFIEAHSTCQSCTKCPPGQGAEKPCEWNENSVCKPCAEGTWSATDSATETCQTCRRCNPGQIEMRLCTATQIPVCCPLHNPNWQDTFAEAEFHEPKSIQNQGQCAEDQAASQLAYGDDYPMITIYCSLLGLVILTLLIYVFYKLWQQRLSAEDAKTIEADVFYPAFMGLKSGGGKSRSSKMSASSKVTTDRQHLLGGLQQYSCTLQNPTLHDALLTELSQGLAVENRWKDVGGLLGRLNYQANIKP : OrigSeq

Jnet : --HHHHHHHHHHHH-----------------------------------------EEE---------EEEE---------------------------------------------------------------------------------------------------------------EEE---------EE--------------------------------------------------------------HHHHHHHHHHH-------HHHHHHHHHHHHHHH----HHHHHHHHHHHHHHHHHHHHHHHHHHHH--------HHHHHHHHHHH-----HHHHHHHHHHEE-------- : Jnet

jhmm : --------------------------------------------------------------------EEE---------------------------------------------------------------------------------------------------------------EE----------EE--------------------------------------------------------------HHHHHHHHHHH------HHHHHHHHHHHHHHHH----HHHHHHHHHHHHHHHHHHHHHH---HH---------HHHHHHHHHHH-----HHHHHHH---EEE------- : jhmm

jpssm : --HHHHHHHHHHHHHHH--------------------------------------EEEE--------EEEE-----EEE---------E----------------------------------------EEE---------EE--------------------------------------EEEEE--------EE---------------------------------------------EEEEE-------HH---HHHHHHHHHHH------------HHHHHHHHH-------HHHHHHHHHHHHHHHHHH--HHHHHH--------HHHHHHHHHHH-----HHHHHHHHHHH--------- : jpssm

Lupas 14 : ------------------------------------------------------------------------------------------------------------------------------------------------------------------------------------------------------------------------------------------------------------------------------------------------------------------------------------------------------------------------------- : Lupas 14

Lupas 21 : ------------------------------------------------------------------------------------------------------------------------------------------------------------------------------------------------------------------------------------------------------------------------------------------------------------------------------------------------------------------------------- : Lupas 21

Lupas 28 : ------------------------------------------------------------------------------------------------------------------------------------------------------------------------------------------------------------------------------------------------------------------------------------------------------------------------------------------------------------------------------- : Lupas 28

Jnet_25 : --BBBBBBBBBBBBBBBBBBBBB-BBBBBB--B---BB--------BB--B--B-BB---B-----B-B--B--B-BBB-----B--B-BB--BB-B--B--B---B--BB-BBBBBB--BBBB--B-BBBBB--BB--BBBB-BB-------B--B--BBBBB--B----B--B--B----BBB--B----B-BB-BB----B-B--B-B-BB-B--B--B--BB---B-B-BBBB-BBBBBB-BB-B-BBBBBB-BBBBBB--BB--B-B--B-BBB--BBB-BB--BB---B----B-BBB-BB--B----B-B-B--B---B--B-BB--BB--B--BB-B---B--BB--BB-B-B---B-- : Jnet_25

Jnet_5 : -------BB----B-BB-B----------------------------B--------------------B--------B---------B-------------------------------------------------------------------------B---------B-----------------------B----------------------------------------------B---B-------B------B---B----------------B--BB-----------------------------------------------BB--B----------------B----------- : Jnet_5

Jnet_0 : -------------B----------------------------------------------------------------------------------------------------------------------------------------------------------------------------------------------------------------------------------------------------------------------------------------------------------------------------------------------------------------- : Jnet_0

Jnet Rel : 3024443211111001336777777777777777765456777764136677763111126777763176157773212367777764314664433112012346777777777777765356777632134677776513313323345677777776545667776776535677775167212677776332367777667777777777777777777777777777654567763212124677541221446899999998515777710112328999987517887046889999999986888753121287105788764278887677511577524666562000114157899 : Jnet Rel

: 1---------11--------21--------31--------41--------51--------61--------71--------81--------91--------101-------111-------121-------131-------141-------151-------161-------171-------181-------191-------201-------211-------221-------231-------241-------251-------261-------271-------281-------291-------301-------311-------321-------331-------341-------351-------361---- :

> Echinococcus multilocularis ISOFORM 2 - pathogen_EmW_scaffold_02.6656(.2)

Jnet Rel : 8210111111110000135777777677677777763304677776666777763111126777763100347773312567777763223676664421222136777775225677776677777531013466654333146777776302467774312156776766535677775167712677776332367777667777777777777777777777777776522567775114212100111023310132017777641001025677777777777777777777777777776545677777777777777777777777766666677777642888874266417764025665556762178999999999873312333687121378777635788732389999986223677774146677766777776545667777889 : Jnet Rel
 : 1---------11--------21--------31--------41--------51--------61--------71--------81--------91--------101-------111-------121-------131-------141-------151-------161-------171-------181-------191-------201-------211-------221-------231-------241-------251-------261-------271-------281-------291-------301-------311-------321-------331-------341-------351-------361-------371-------381-------391-------401-------411-------421-------431-------441-------451-------461 :

OrigSeq : MLILLLILTLLSSFRWIQFNGGPEFMPETCPSANEELVSTLNGPLRCCNKCPSGEGMLQLCTNQTQTVCRPCQEGSEFSLEASATAKCMQCRQCQELHPFAKFRKHCTPTSDAVCECVSGYFFIEAHSTCQSCTKCPPGQGAEKPCEWNENSVCKPCAEGTWSATDSATETCQTCRRCNPGQIEMRLCTATQIPVCCPLHNPNWQDTFAEAEFHEPKSIQNQGQCAEDQAASQLAYGDDYPMITIYCSLLGLVILTLLIYVFYKLWQQRLSAEDAKTIEADVFYPAFMGLKSGGGKSRSSKMSASSKVTTDRQHLLGGLQQYSCTLQNPTLHDALLTELSQGLAVENRWKDVGGLLGFSEESLQNFEVGASEKTSNSVDSAAVATRLMLTSWYSARAATDADPLRSLLMVLGCTPSTGHLCRWLKEYMKHSSIAPCYAIPPTESPSQSPATAISPEAQNISNH : OrigSeq

Jnet : --EHHHHHHHHHHHH----------------------------------------EEE----------------------------------------------------------------------------------------------------------------------------EEEE--------EE----------------------------------------------------EEEEEE----EEEEE---------H---------------------------------------------------------------------------HHHHHHH----HHHHH-------------HHHHHHHHHHHHHHHH--------HHHHHHHHHH------HHHHHHHHHHHH---------------------------------- : Jnet

jhmm : -EEEE---------------------------------------------------------------------------------------------------------------------------------------------------------------------------------EEE---------EE------------------------------------------------EEEEEEEEHHHHHEEEEEEE------------------------------------------------------------------------------------HHHHHHH-----HHHH-----------HHHHHHHHHHHHHHHH---------HHHHHHHHHHH------HHHHHHHHHHHH---------------------------------- : jhmm

jpssm : --HHHHHHHHHHHHHHHH-------------------E-----------------EEEE--------EEEE-----E-----------EE---------HHHH-------------------------EEEEE--------E----------EE------EEE------------------EEEEE--------EE--------------------------------------------------------EE----------------HHHH--------------------------------------------------------------------------HHHHHHH----HHHHHH------------HHHHHHHHHHHHHHHHHHH------HHHHHHHHH-------HHHHHHHHHH----------------------------------- : jpssm

Lupas 14 : ------------------------------------------------------------------------------------------------------------------------------------------------------------------------------------------------------------------------------------------------------------------------------------------------------------------------------------------------------------------------------------------------------------------------------------------------------------------------------- : Lupas 14

Lupas 21 : ------------------------------------------------------------------------------------------------------------------------------------------------------------------------------------------------------------------------------------------------------------------------------------------------------------------------------------------------------------------------------------------------------------------------------------------------------------------------------- : Lupas 21

Lupas 28 : ------------------------------------------------------------------------------------------------------------------------------------------------------------------------------------------------------------------------------------------------------------------------------------------------------------------------------------------------------------------------------------------------------------------------------------------------------------------------------- : Lupas 28

Jnet_25 : ---BBBBBBBBBBBBBBBBBB-B-BBBBBB--B---B---B---B-BB--B--B-BB---B-----B-BBBB--B--B------B--B--B----BB----BB---B--B--BB-BBB--BBBB--BBBB--B-BBBB---BBB-B----B-BB--B--BBBBB--BB---B--B--B-----BB--B----B-BB--B----B----B-B-BB-B--B----BB--B-B-B-B-BB-B-BBBBBBBBBBBBBBBBBB--BBB--B--B-----B----B--B--BBBBB---------B-BBB-B-BBBB---B---------------BB--BB------------B--BBB-B-B----B--B----B---B--BBBBB-BBB-BB--BBB--BB----BBBBBBBBB-B------BB-BB--BB----BBBBBBBB------------B---B--B--- : Jnet_25

Jnet_5 : -------BB------BB-B----------------------------B--B-----------------B------------------B-----------------------------------------------------------------------------------B-----------------------B----------------------------------------------B---BBBBBBB-----------------------------------------------------------------------------------------------B--BB--B---------------------B--BB-BB--BB--------------B-BBB-BB--------BB--B---B-------B--B------------------------ : Jnet_5

Jnet_0 : ----------------------------------------------------------------------------------------------------------------------------------------------------------------------------------------------------------------------------------------------------------BB---------------------------------------------------------------------------------------------------BB---------------------------B---B---------------------B------------B---B--------------------------------------- : Jnet_0

Jnet Rel : 8210111111110000135777777677677777763304677776666777763111126777763100347773312567777763223676664421222136777775225677776677777531013466654333146777776302467774312156776766535677775167712677776332367777667777777777777777777777777776522567775114212100111023310132017777641001025677777777777777777777777777776545677777777777777777777777766666677777642888874266417764025665556762178999999999873312333687121378777635788732389999986223677774146677766777776545667777889 : Jnet Rel

: 1---------11--------21--------31--------41--------51--------61--------71--------81--------91--------101-------111-------121-------131-------141-------151-------161-------171-------181-------191-------201-------211-------221-------231-------241-------251-------261-------271-------281-------291-------301-------311-------321-------331-------341-------351-------361-------371-------381-------391-------401-------411-------421-------431-------441-------451-------461 :

> Echinococcus granulosus

Jnet Rel : 95300111110231310135787531204677777653256777766667777631013467777641551477733333667777643040000125667654246677751156776322057776403465125643222057777765325667776777777777765333577731110136777763223677776677777777777777777777777777777776677777751253001100101111001325720177777777643101101133677751333333211057869999886113221678877640788873112300275023102001123677227777777227777227676611113366677777777764166788888741313434677651002034551588871278999986231677751204677766777642125667777889 : Jnet Rel
 : 1---------11--------21--------31--------41--------51--------61--------71--------81--------91--------101-------111-------121-------131-------141-------151-------161-------171-------181-------191-------201-------211-------221-------231-------241-------251-------261-------271-------281-------291-------301-------311-------321-------331-------341-------351-------361-------371-------381-------391-------401-------411-------421-------431-------441-------451-------461-------471-------481----- :

OrigSeq : MLIPLLIWTLLSSFRWIRFNGGPEFMQETCPSANEELVSTLNGPLRCCNKCPSGEGMLQLCTNQTQTVCRPCQEGSEFSLEASATAKCMQCRQCQELHPFAKFRKHCTPTSDAVCECVSGYFFIEAHSTCQSCTKCPPGQGAEKPCEWNENSVCKPCAEGTWSSTDSATDTCQTCRRCKPGQIEMRPCTATQNTLCCPLHNPNCQDTFAEEEFDEPKSMQSQGQCAEDQAASQLAYGDDYPMITIYCSLLGLVILTLLIYVFYKLWQQRLSAEDAKTIEADVFYPAFMGLKSGKGKSRSSKMSAPSKVTTDRQHLLGGLQQYSCTPQNPTLHDALLTELSQGLAVENRWKHVGGLLGRLNYQANIKPYEKTQSLPSITFTGFSEESLQNFEKVGASEKTSNSADSAAVAARLMLTSWYSARAATDPDPLRSLLMVLGCTPSTGHLCRCLKEYMKYSSIAPCYAIPPTEPPSQSPATAISPEAQNTNNH : OrigSeq

Jnet : ----HHHHHH----------------------------------------------------------EEE---------------------------------------------------------------------------------------------------------------EEE----------------------------------------------------------------HHHH--HHHHHHH-------------------------------------------HHHHHHHHHHHHHHHHHH---------HHHHHHH---E-----HHH------------------------------------------------------HHHHHHHHHHHHH----------HHHHEEEE------HHHHHHHHHHHHH--------------------------------- : Jnet

jhmm : --------------------------------------------------------------------EEE----------------------------------------------------------------------------------------------------------------------------------------------------------------------------------------------------EEE------------------------------------HHHHHHHHHHHHHHHHH---------HHHHH-----EEE---EE--------E---EE-------EE----EE-----HHHHHH---------------HHHHHHHHHHHHH------------HHEEEE------HHHHHHHHHHHHH--------------------------------- : jhmm

jpssm : --HHHHHHHHHHH-HEEEE-----EEE----------------------------EEEE--------EEEE-----EEE---------E-------------------------------EE--------E--------EEEE--------------------------------------EEEEE--------EE----------------------------------------------------HHHHHHHHHHHHHHHH----------------HHHHHHHHH-------HHHHHHHHHHHHHHHHHHHHHHHHHH---------HHHHHHHHHHH-----HHHHHHHHHHH----------------------------------------------HHHHHHHHHHHH------------HHHHEEEEE------HHHHHHHHHH--------E-------------------------- : jpssm

Lupas 14 : -------------------------------------------------------------------------------------------------------------------------------------------------------------------------------------------------------------------------------------------------------------------------------------------------------------------------------------------------------------------------------------------------------------------------------------------------------------------------------------------------------- : Lupas 14

Lupas 21 : -------------------------------------------------------------------------------------------------------------------------------------------------------------------------------------------------------------------------------------------------------------------------------------------------------------------------------------------------------------------------------------------------------------------------------------------------------------------------------------------------------- : Lupas 21

Lupas 28 : -------------------------------------------------------------------------------------------------------------------------------------------------------------------------------------------------------------------------------------------------------------------------------------------------------------------------------------------------------------------------------------------------------------------------------------------------------------------------------------------------------- : Lupas 28

Jnet_25 : --BBB-B-BBBBBBBBBBB--BB-BB-B-B--B---BBB-------BB--B--B-BBB--B------BBBBB----BB------B--B--B-B--B---BBBB-----BBB-BB-BBB-BBBBB--B-B-B-BBBBBB--BBB--B----BBB---B--BBBBB-------B--B-----BBBBB--B----BBBB--B--B-B--BBB---BBBBB-B-----B---BB-BBBBBBBB--BBBBBBBBBBB-B----BBBBB-B---B-B---B----B-BBB-BBB-B--B-BB--BB-B--BB-B---B--BB--B--B-B----B-B--BBB--B---B-B---B--B---B--B-B---B--------BB-BBB-BB---BB--B--B-B---------BBBBBB-BBBBBBB-B-B-----BB-BBBBBB-BB-----BB-BB--BB----BBBBBBBB-----------BB---B------ : Jnet_25

Jnet_5 : ---------------B--B----------------------------B--B-----------------B--B---------------B-----------------------------------------------------B-----------------------------B-----------------------B-------------------------------------B-------B--B-BB-------------B--------------------B------B---------------------------------------------B---------------B---B-------------------------------------------------B-B-B---B--------------B--BBBBB--------BB--B---B-------B--B------------------------ : Jnet_5

Jnet_0 : ------------------------------------------------------------------------------------------------------------------------------------------------------------------------------------------------------------------------------------------------------------------------------------------------------------------------------------------------------------------------------------------------------------------------------------------------------------B---B--------------------------------------- : Jnet_0

Jnet Rel : 95300111110231310135787531204677777653256777766667777631013467777641551477733333667777643040000125667654246677751156776322057776403465125643222057777765325667776777777777765333577731110136777763223677776677777777777777777777777777777776677777751253001100101111001325720177777777643101101133677751333333211057869999886113221678877640788873112300275023102001123677227777777227777227676611113366677777777764166788888741313434677651002034551588871278999986231677751204677766777642125667777889 : Jnet Rel

: 1---------11--------21--------31--------41--------51--------61--------71--------81--------91--------101-------111-------121-------131-------141-------151-------161-------171-------181-------191-------201-------211-------221-------231-------241-------251-------261-------271-------281-------291-------301-------311-------321-------331-------341-------351-------361-------371-------381-------391-------401-------411-------421-------431-------441-------451-------461-------471-------481----- :

> Echinococcus Canadensis

Jnet Rel : 99721111100221011336777777776777776412346777653567777631013467777631003577732223677777210144322222336777777777777777777533357776512567777651010315777776533356777777777777765356777730321137787721013677776677777777777777777777777777777676777775112231011111111111011677872266431010256114650010256776766663257777652000102023567777777652288876771315675245541230314673777777777777775456676666666645667777777612789999998731013434677651002034551588871278999986223677751214677766777765325667777889 : Jnet Rel
 : 1---------11--------21--------31--------41--------51--------61--------71--------81--------91--------101-------111-------121-------131-------141-------151-------161-------171-------181-------191-------201-------211-------221-------231-------241-------251-------261-------271-------281-------291-------301-------311-------321-------331-------341-------351-------361-------371-------381-------391-------401-------411-------421-------431-------441-------451-------461-------471-------481----- :

OrigSeq : MLIPFLVWTLLSSFRWIRFNGGPEFMQETCPSANEELVSTLNGPLRCCNKCPSGEGMLQLCTNQTQTVCRPCQEGSEFSLEASATAKCMQCRQCQELHPFAKFRKHCTPTSDAVCECVSGYFFIEAHSTCQSCTKCPPGQGAEKSCEWNENSVCKPCAEGTWSSTDSATDTCQTCRRCKPGQIEMRPCTATQNTLCCPLHNPNCQDTFAEEEFDEPKSMQSQGQCAEDQAASQLAYGDDYPMITIYCSLLGLVILTLLIYVFYKLWQQRLSAEDAKTIEADVFYPAFMGLKSGGGKSRSSKMSASSKVTTDRQHLLGGLQQYSCTPQNPTLHDALLTELSQGLAVENRWKHVGVLLGRLNYQANIKPYEKTQSLPSITFTGFSEESLQNFEKVGASEKTSNSVDSAAVATRLMLTSWHSARAATDPDPLRSLLMVLGCTPSTGHLCRCLKEYMKHSSIAPCYAIPSTEPPSQSPATAISPEAQNTNNH : OrigSeq

Jnet : ----HHHHHH---------------------------------------------------------------------------------------------------------------------------------------------------------------------------EEEE---------------------------------------------------------------HHHHHHHHHHHHH---------------------E----E------------------------HEEE----------------HHHHHHHHH------HHHHHHHHH-----------------------------------------------HHHHHHHHHHHHHH-----------HHHHEEEE------HHHHHHHHHHHH-------EE------------------------- : Jnet

jhmm : --------------------------------------------------------------------------------------EE------------------------------------------------------------------------------------------------------------------------------------------------------------------------------------EE-----------EE-----------------------------EEEE-----------------HHHHHHH-------HHHHHHHH------H----------------------------------------HHHHHHHHHHHHHHHH------------HHEEEE------HHHHHHHHHHHH-------EE------------------------- : jhmm

jpssm : ---HHHHHHHHHHHHHHH-------------------EE----------------EEEE--------EEEE-----EEE------------HHHHHHHH--------------------------------------------E-------------------------------------EEEEE-------EEE------------------------------------------------EEHHHHHHHHHHHHHHHH-------------------------E-----------------------HHHHHH---------------HHHHHHHHHH-----HHHHHHHHHH-----------------------------------------------HHHHHHHHHHHH------------HHHHEEEEE------HHHHHHHHHH--------E-------------------------- : jpssm

Lupas 14 : -------------------------------------------------------------------------------------------------------------------------------------------------------------------------------------------------------------------------------------------------------------------------------------------------------------------------------------------------------------------------------------------------------------------------------------------------------------------------------------------------------- : Lupas 14

Lupas 21 : -------------------------------------------------------------------------------------------------------------------------------------------------------------------------------------------------------------------------------------------------------------------------------------------------------------------------------------------------------------------------------------------------------------------------------------------------------------------------------------------------------- : Lupas 21

Lupas 28 : -------------------------------------------------------------------------------------------------------------------------------------------------------------------------------------------------------------------------------------------------------------------------------------------------------------------------------------------------------------------------------------------------------------------------------------------------------------------------------------------------------- : Lupas 28

Jnet_25 : --B-BBB-BBBBBBBBBBB--B--BBB-BB--B---BB----B-B-BB--B--B-BBB--B-----B-B-BB--B-BB----B----B--B--B--B--BBBB--BBBBB--BB-BB---BBBBB-B--BB-BBBBB-BBBBB-BB------BB--B--BBBBB-------B--B-----B-BBB--B---BBBBB--B--B-B--BBB---BB-BB-BB--B-B---BBBB-BBBBBB--BBBB-BBBBBBBB-BB--B-BB-BB--B-B---B--B-B-BBB-BBB-B-----BB----B------B-----BBB-B--B-B----B-B---BBB-B--BB-B--BB--BBB-BB-B-B-B-B--------B--B-B-BB---BB--B--B-B---B---B--BBBBB-BBBBBBB-B-B-----BB-BBBBBB-BB-----BB-BB--BB----BBBBBBB---------B--BB---B------ : Jnet_25

Jnet_5 : ------------B--B--B----------------------------B--B-----------------B------------------B-----------------------------------------------------------------------------------B-----------------------B-------------------------------------B------------B-BB--B--------B--------------------B--B----------------------------B---B---------------BB--B------------BB--B----------------------------------------------------BB--BB--------------B--BBBBB--------BB--B-----------B--B------------------------ : Jnet_5

Jnet_0 : ----------------------------------------------------------------------------------------------------------------------------------------------------------------------------------------------------------------------------------------------------------B----------------------------------------------------------------------------------------------------B--------------------------------------------------------------------------------------------B---B--------------------------------------- : Jnet_0

Jnet Rel : 99721111100221011336777777776777776412346777653567777631013467777631003577732223677777210144322222336777777777777777777533357776512567777651010315777776533356777777777777765356777730321137787721013677776677777777777777777777777777777676777775112231011111111111011677872266431010256114650010256776766663257777652000102023567777777652288876771315675245541230314673777777777777775456676666666645667777777612789999998731013434677651002034551588871278999986223677751214677766777765325667777889 : Jnet Rel

: 1---------11--------21--------31--------41--------51--------61--------71--------81--------91--------101-------111-------121-------131-------141-------151-------161-------171-------181-------191-------201-------211-------221-------231-------241-------251-------261-------271-------281-------291-------301-------311-------321-------331-------341-------351-------361-------371-------381-------391-------401-------411-------421-------431-------441-------451-------461-------471-------481----- :

> Taenia asiatica

Jnet Rel : 962011100220001336777777777654566777676777777654567776411367777631103467777653221577733333667676654567777777643222234677752156777652256777777777776431111121577777545677632121567767665356777731783037787721643677776677777777777777777777777777777666777774202411131444322221124777752122336777777777777777777710177777777777777777777771127776522899999885167887764123432146763113211244677777777777764137899931000115771689999998625888625689999998530777777777777777777776545667777889 : Jnet Rel
 : 1---------11--------21--------31--------41--------51--------61--------71--------81--------91--------101-------111-------121-------131-------141-------151-------161-------171-------181-------191-------201-------211-------221-------231-------241-------251-------261-------271-------281-------291-------301-------311-------321-------331-------341-------351-------361-------371-------381-------391-------401-------411-------421-------431-------441-------451-------461-------471- :

OrigSeq : MSILLLIWTLISSFWCSALPQALHQPLNSTQLDPIQQETCPSANEELVSTLDGPLRCCNKCPPGEGMLQLCTNQTQTVCRPCQEGSELSLEASATAKCMQCKQCQELHPFAKFRQHCTPTSDAVCECVSGYFFIEALSTCQSCTKCPPGQGAEKPCGWNENTVCKPCAEGTWSSTDSATDICQTCRRCKPGQIEMRPCTATQNTLCCPLHNPNCEDTFEEEELDEPKSIQSQGKCTDDQAASQLAYGDDYPMITIYCSLLGLVILTLLIYVFYKLWQQRLSAEDAKTIEADVCYPAFTGLKSGRDKLRSSKISAPSKDTTDRQHLLGGSQQYSYPPQNPTIHEALLTELSQGLAVENRWKHVGGLLGFNEESLQNFEKVGASDETSNPVDSAAVVTRLMLTSWYSARAATDPNPLTSLLMVLERTPSTGHLCRCLKEYIKQSSTSSPYAMPPTQPPSQLPTSAISPEAQNTSNH : OrigSeq

Jnet : ---HHHHH---HH------------------------------------------EE--------EE----------------------------------------------------------------------------------------------------------------------------EEEE--------EEE-----------------------------------------------EEEE-HHHHHHHHHHHHH-------------------------------------------------------------------HHHHHHHHHH----------HHHHHH------HHHH-------------------HHHHHHHHHHH------HHHHHHHHHHHH------HHHHHHHHHHHH---------------------------------- : Jnet

jhmm : -------------------------------------------------------EE---------------------------------------------------------------------------------------------------------------------------------------EE----------EE-------------------------------------------------EEEHHHHHHHHHHHHH---------------------------------EEE----------------------EEE-------HHHHHHHHH----------HHHHHH-----------------------------HHHHHHH----------HHHHHHHHHHHH------HHHHHHHHHHHHH--------------------------------- : jhmm

jpssm : --HHHHHHHHHHHHHH----------------------------------------E--------EEEE--------EEE------EEE---------------------HHHHHH--------------------------------HHHHHHH---------------EEE------------------EEEEE-------EEE----------------------------------------------EEEE----H---------EE-------HHHH-------------------------------------------------------HHHHHHHHHH---------HHHHHHH-----HHHHHHHH------------------HHHHHHHHHHH-----HHHHHHHHHHH-----HHHHHHHHHHHH----------------------------------- : jpssm

Lupas 14 : ------------------------------------------------------------------------------------------------------------------------------------------------------------------------------------------------------------------------------------------------------------------------------------------------------------------------------------------------------------------------------------------------------------------------------------------------------------------------------------------ : Lupas 14

Lupas 21 : ------------------------------------------------------------------------------------------------------------------------------------------------------------------------------------------------------------------------------------------------------------------------------------------------------------------------------------------------------------------------------------------------------------------------------------------------------------------------------------------ : Lupas 21

Lupas 28 : ------------------------------------------------------------------------------------------------------------------------------------------------------------------------------------------------------------------------------------------------------------------------------------------------------------------------------------------------------------------------------------------------------------------------------------------------------------------------------------------ : Lupas 28

Jnet_25 : --B--B-BBBBBBB-B--BB-BBB-B--B--B--B-B-BB--B---B------BB-BB--B--B-BBB--B-------BB-B--B-BB----B----B--B--------BBBBB--B--BBBBBBBBB---BBB--B---BB-B-B--B-BBB-BB--B-B--B--B--BBBBB-------B--B--B----BBB--B---BBBBB--B----B--B------BBBB-B--B--B-----BB--BBB---BBBBBBBBBBBB-B---B-BBBB-BB----B-----------B-------------B--------------------------B-------B--BB--B---------BB-BBB-B-B---BB--B------------B-----BBBBBBB-BB---B-BB--BB-BBB-B---------BB-BB--BB-------BB-B--------B---BB---B------ : Jnet_25

Jnet_5 : -----B--B------B--B--------------------B-----------------B--B-----------------B------------------B-------------------------B---------------------------------------------B-B---------B-----------------------B--------------------------------------------------BBBBB-B---------------------------------------------------------------------------------BB------------B--BB--B----------------------------BB--BB---B---------BB-BB--B---------BB--B---B----------------------------------- : Jnet_5

Jnet_0 : -----------------------------------------------------------------------------------------------------------------------------------------------------------------------------------------------------------------------------------------------------------------B--------------------------------------------------------------------------------------B----------------BB--------------------------------B-BB--------------B---B----------------B--------------------------------------- : Jnet_0

Jnet Rel : 962011100220001336777777777654566777676777777654567776411367777631103467777653221577733333667676654567777777643222234677752156777652256777777777776431111121577777545677632121567767665356777731783037787721643677776677777777777777777777777777777666777774202411131444322221124777752122336777777777777777777710177777777777777777777771127776522899999885167887764123432146763113211244677777777777764137899931000115771689999998625888625689999998530777777777777777777776545667777889 : Jnet Rel

: 1---------11--------21--------31--------41--------51--------61--------71--------81--------91--------101-------111-------121-------131-------141-------151-------161-------171-------181-------191-------201-------211-------221-------231-------241-------251-------261-------271-------281-------291-------301-------311-------321-------331-------341-------351-------361-------371-------381-------391-------401-------411-------421-------431-------441-------451-------461-------471- :

> Taenia saginata

Jnet Rel : 214688202333033156777777777777777777777777777777777777776667777631103467777631003577732223677776633046766766640010036777751014777775101567777777776310111101236777777777777777777777765356777731783037787721643677776677777777777777777777777777777666777763453361011101001012777701378999931033677777777777777777777765116677767777777777777777642299999885267887770241321306762167753115777777777776640348999841324046751899999998626737899999998753067777777777777777777776545667777889 : Jnet Rel

: 1---------11--------21--------31--------41--------51--------61--------71--------81--------91--------101-------111-------121-------131-------141-------151-------161-------171-------181-------191-------201-------211-------221-------231-------241-------251-------261-------271-------281-------291-------301-------311-------321-------331-------341-------351-------361-------371-------381-------391-------401-------411-------421-------431-------441-------451-------461-------471- :

OrigSeq : MSILLLIWTLISSFWCSALPQALHQPLNSTQLDPIQQETCPSANEELVSTLDGPLRCCNKCPPGEGMLQLCTNQTQTVCRPCQEGSEFSLEASATAKCMQCKQCQELHPFAKFRQHCTPTSDAVCECVSGYFFIEALSTCQSCTKCPPGQGAEKPCGWNENTVCKPCAEGTWSSTDSATDICQTCKRCKPGQIEMRPCTATQNTLCCPLHNPNCEDTFEEEELDEPKSIQSQGRCTDDQAASQLAYGDDYPMITIYCSLLGLVILTLLIYVFYKLWQQRLSAEDAKTIEADVCYPAFTGLKSGRDKLRSSKISAPSKDTTDRQHLLGGSQQYSYPPQNLTIQEALLTELSQGLAVENRWKHVGGLLGFNEESLQNFEKVGASDETSNPVDSAAVATRLMLTSWYSARAATDPNPLTSLLMVLERTPSTGHLCRCLKGYIKQSSTSSPYAMPPTQPPSQLPTSAISPEAQNTSNH : OrigSeq

Jnet : --HHHHH----------------------------------------------------------EE----------------------------------------------------------------------------------HHHHHH------------------------------------EEEE--------EEE----------------------------------------------EEE----EEEE------------HHHHHHHHHH------------------------------------------------------HHHHHHHHHH-------HHHHHHHH-----HHHHHHH-----------------HHHHHHHHHHHHH-----HHHHHHHHHH----HHHHHHHHHHHHH------------------------------------ : Jnet

jhmm : HHHHHH------------------------------------------------------------------------------------------------------------------------------------------------------------------------------------------EE----------EE----------------------------------------------EEE--EEEEEEEEEEEEE----HHHHHHHHH-----------------------------EE--------------------------HHHHHHHHH-------HHHHHHHHH-----HHHHHH-----------------HHHHHHH--H-HH-----HHHHHHHHHH----HHHHHHHHHHHHHH----------------------------------- : jhmm

jpssm : --HHHHHHHHHH-HH--------------------------------------------------EEEE--------EEEE-----EEE---------E-------------------------------------------------HHHHHHHHH----------------------------------EEEEE-------EEE----------------------------------------------EEE----------------------HHHHHHHHHH---------------------------------------------------HHHHHHHHHH-----------HHHH------HHHHHHH----------------HHHHHHHHHHHHH-----HHHHHHHHHHHH--HHHHHHHHHHHHHH------------------------------------ : jpssm

Lupas 14 : ------------------------------------------------------------------------------------------------------------------------------------------------------------------------------------------------------------------------------------------------------------------------------------------------------------------------------------------------------------------------------------------------------------------------------------------------------------------------------------------ : Lupas 14

Lupas 21 : ------------------------------------------------------------------------------------------------------------------------------------------------------------------------------------------------------------------------------------------------------------------------------------------------------------------------------------------------------------------------------------------------------------------------------------------------------------------------------------------ : Lupas 21

Lupas 28 : ------------------------------------------------------------------------------------------------------------------------------------------------------------------------------------------------------------------------------------------------------------------------------------------------------------------------------------------------------------------------------------------------------------------------------------------------------------------------------------------ : Lupas 28

Jnet_25 : --B--BBBBBBBBB-B--BB-BBBBBB-B--B--B---BB--B--BB-------B-BB--B--B-BB---B-------B-BB--B--B------B--B--BB-B--B--BB-BB-BBBBB-BBB-BB--BBBBB-BB--BB-B-BB----BBB-BB-BB----B--BB-BBBBB-------B--B--B----BBB--B---BB-BB--B----B--B---B-BB----B--B--B-----B--BBBBB---BBBBBBBBBBBBBBBBBBBB-B--B---B------------B----------------B-BBBBB--B-------------BB--B----B--BB--B--B------BB-BBB-B-B----B--B--------B--BB--B--BBBBBBBBBB---BBBB--BB-BBB-B------B--BB-BB--BB-------BB-B--------B---BB---B------ : Jnet_25

Jnet_5 : ---------------B--B--------------------B-----------------B--B-----------------B------------------B-------------------------------------------------------------------------B---------B-----------------------B----------------------------------------------BB--BBBBBBBBB-------------------------------------------------------------------------------B-------------B--BB--B----------------------------BB--BB-------------BB-BB--B---------B---B--------------------------------------- : Jnet_5

Jnet_0 : -------------------------------------------------------------------------------------------------------------------------------------------------------------------------------------------------------------------------------------------------------------------BB-----------------------------------------------------------------------------------B-----------------B--------------------------------B--B--------------B---B------------B------------------------------------------- : Jnet_0

Jnet Rel : 214688202333033156777777777777777777777777777777777777776667777631103467777631003577732223677776633046766766640010036777751014777775101567777777776310111101236777777777777777777777765356777731783037787721643677776677777777777777777777777777777666777763453361011101001012777701378999931033677777777777777777777765116677767777777777777777642299999885267887770241321306762167753115777777777776640348999841324046751899999998626737899999998753067777777777777777777776545667777889 : Jnet Rel

: 1---------11--------21--------31--------41--------51--------61--------71--------81--------91--------101-------111-------121-------131-------141-------151-------161-------171-------181-------191-------201-------211-------221-------231-------241-------251-------261-------271-------281-------291-------301-------311-------321-------331-------341-------351-------361-------371-------381-------391-------401-------411-------421-------431-------441-------451-------461-------471- :

> Mesocestoides corti

Jnet Rel : 994122010000126777777777667777642333667776677777667777640123677765356777763100126777763166146776545667777764315763322222000013267633222303677777777777777777765402315677777767777776545667776776535677773178213677776322367777667777777777777777777777777777677767764221023667776677101105653565122222211110177777777777777777777710017777777777777777777777777777777777776422999998851677776512210277776504421146777777777777653114588886024123577752348999999861188710688831021227777777777889 : Jnet Rel
 : 1---------11--------21--------31--------41--------51--------61--------71--------81--------91--------101-------111-------121-------131-------141-------151-------161-------171-------181-------191-------201-------211-------221-------231-------241-------251-------261-------271-------281-------291-------301-------311-------321-------331-------341-------351-------361-------371-------381-------391-------401-------411-------421-------431-------441-------451-------461-------471------- :

OrigSeq : MLNTQLLPTLIALLACHEALTLPTATGRTGQHLATSTNSTGLDHIQQETCPNANEEIVLTMSGQLKCCVKCPAGEGMLQLCTNHTQTVCRTCQEFSEFSPVASATAKCMQCKQCQELHPFAKFRTHCTPTSDAVCECIPGYFFVEAQSTCQSCTKCPPGHGAERPCDWNRDSLCKPCPAGTWSSSTSATETCQTCRKCKPGQIEMRPCSATHNTLCCPLHEPNCSDTLEMDAIRDEPSQTNRRQATGDQMPDHVAYGDDYPMITVYCSLLGLVILTLLIYVFYKLWQQHLTAVDAKAGMVEAAGGNALHPAFAELTKSRSVSAATSGHSSLSKQKASAASRPDCVRQHLLSESTQYSSLPQRPPISSELMEELSQALSADDRWKQVGGMLGYSDEALQRFENSSEHENDDVTKETTGATVAARRMLSSWFASRPSTETNPLESLISVIELIPGTANLCVLLKEYSSVKSPPPHPPLGQPH : OrigSeq

Jnet : ----EEEHHH------------------------------------------------------------------EE---------EEEE--------------------------------------------------------------------------------------------------------------EEEE--------------------------------------------------------EEEE------------EEEE-------HHHHHHHHHHHH-----------------------EE----------------------------------------HHHHHHHHH--------HHHH---------HHHH-------------------HHHHHHHHH----------HHHHHHHHHHHHH-----HHHHHHH------------------ : Jnet

jhmm : --EEEEE---------------------------------------------------------------------------------EEE---------------------------------------------------------------------------------------------------------------EE---------------------------------------------------------EEE------------EEEEE-------HHHHHHHHHHHHH---------------------EEEE----------------------------------------HHHHHHHH--------HHHHHH------HHHEE--------------------HHHHHHH-----------HHHHHHHHHHHHHH---HHHHHH-----EE------------- : jhmm

jpssm : -----HHHHHHHH--------------------EE----------------------EE----------------EEEE--------EEEE-----------------E-------HHHHHHHHHH----HHHHHH-------------------------EE--------------------------------------EEEEE--------EE------------------------------------------------EE--------------------------------------------------------------------------------------------------HHHHHHHHHH---------------------HHHH------------------HHHHHHHHHH----------HHHHHHHHHHHHH------HHHHHHHH---------------- : jpssm

Lupas 14 : ------------------------------------------------------------------------------------------------------------------------------------------------------------------------------------------------------------------------------------------------------------------------------------------------------------------------------------------------------------------------------------------------------------------------------------------------------------------------------------------------ : Lupas 14

Lupas 21 : ------------------------------------------------------------------------------------------------------------------------------------------------------------------------------------------------------------------------------------------------------------------------------------------------------------------------------------------------------------------------------------------------------------------------------------------------------------------------------------------------ : Lupas 21

Lupas 28 : ------------------------------------------------------------------------------------------------------------------------------------------------------------------------------------------------------------------------------------------------------------------------------------------------------------------------------------------------------------------------------------------------------------------------------------------------------------------------------------------------ : Lupas 28

Jnet_25 : ---BBBBBBBBBBB-B--B--B--BBB-B---BBBBB--B---BB---BB--B---BBB-------BB--B--B-BBB--B-------B--B-----B------B--B--B--------B--BB---B-B--BBB-BB-BBBBBB---BBBBB--BB-B-BBBB-B-B--------B--BBBB----B---B--B--B--B--BB--B----BB-B--B--B-BB-BB---BB-----------B---BBBBBBB-----BBBB-BBB---B--B-B-BB-BB-BB-----B--B---B-----B---B--B--B--B--BB-BBB----------------BB----B---B-BBB-B----------B--B---B-----BB-BBB-B-B---BB--B----------B----B-B-BBBBBBBBBBB----B----BBBBBBBBBB-B-----BB-BB---B-B-BB---------- : Jnet_25

Jnet_5 : ------B-BBBBB--B--B-------------BB-B-------------B-----------------B--B-----------------B--B-----B---------B-------------------------------------------------------------------------B---------B-----------------------B----------------------------------------------------------------------------------------------------------------------------------------------------------------------B--BB--B------------------------------BB--BB---B----------B-BBB-BB--------BB--B------------------- : Jnet_5

Jnet_0 : -------------------------------------------------------------------------------------------------------------------------------------------------------------------------------------------------------------------------------------------------------------------------------------------------------------------------------------------------------------------------------------------------------------------------------------B--B---------------B--B---B-------------------------------- : Jnet_0

Jnet Rel : 994122010000126777777777667777642333667776677777667777640123677765356777763100126777763166146776545667777764315763322222000013267633222303677777777777777777765402315677777767777776545667776776535677773178213677776322367777667777777777777777777777777777677767764221023667776677101105653565122222211110177777777777777777777710017777777777777777777777777777777777776422999998851677776512210277776504421146777777777777653114588886024123577752348999999861188710688831021227777777777889 : Jnet Rel

: 1---------11--------21--------31--------41--------51--------61--------71--------81--------91--------101-------111-------121-------131-------141-------151-------161-------171-------181-------191-------201-------211-------221-------231-------241-------251-------261-------271-------281-------291-------301-------311-------321-------331-------341-------351-------361-------371-------381-------391-------401-------411-------421-------431-------441-------451-------461-------471------- :

> Schistosoma rodhaini

Jnet Rel : 8368999874177763311210133213315777752356331466553564134225667771277777710544443110331467777777777776677711124134632116777624613677765356777763164367777776310136771112467777763223666666667777777777777777777777777777777777771016502046777667622777764200667776776535677773178303778772101367777667777777777777777772277766777776304106777532230677411127777777777767777751110337722266665456666666667777777765278888861267787701227877411267310001325777766566554432102763689999998703518834210244432245641223899999310330139998751578877500431477763331278999985318737899999987437742577777640122147887766400103024577777642278 : Jnet Rel
 : 1---------11--------21--------31--------41--------51--------61--------71--------81--------91--------101-------111-------121-------131-------141-------151-------161-------171-------181-------191-------201-------211-------221-------231-------241-------251-------261-------271-------281-------291-------301-------311-------321-------331-------341-------351-------361-------371-------381-------391-------401-------411-------421-------431-------441-------451-------461-------471-------481-------491-------501-------511-------521-------531-------541-------551-------561-------571-------581-------591-------601------- :

OrigSeq : MYRIYQFVHSFDAHTKHTITCMHNSKLFIELLCLTIVWNSVIAGPLIFQSEILEGKIYPPVNTSQINNSNNTISXXXXXXXXXXXXXXGNENNETVATTVGTTEGTGDIVEIQTETCDDPLEEFVSPVRGTPRCCRKCEPGNGMLRLCSNTEDTQCRPCKPGFEFSPFRSATKKCLQCRRCEELHPLAKTRNECTPVTDTICQCEKPYYMSEKEQTCKPCTVCKPGEGIVQACGWNSDTQCQSCPAGFWSAQSIDNVKCIPCQSCGKDQILVKECSSTSDTLCCPVNNPNCTHELSMYFDYSAYDQESDISDNNNKSNQMLPIYCSIMGLIIISLLCYVVYKLWRQREASKNSKLTDSYNSNKTDLLDRTSCLDHNHLQHRRISSGFVNNNAHLPNNSPQSATELNNTVDSTPDNDINNHLQFNDIIVGHEKAPLLGKLDHSNSSFNNFEQQPITVIPMNILGVICYRLSQHGWQELVNILDLETSKFDHLPSEITSDLLSATMGAQTTAESHLKQCNQDNSNTIQPITNNNNNNLTMTVSMFQYMCLQNTVNLGQLMNSLQKLNRPDLVSLIQQQIGIIKSKKTINQSNEEYKTKTKSIKSKENFQIEN : OrigSeq

Jnet : --HHHHHHHHH-------EEEE---HHHHH-------------------------------------------------EEE-----------------------EEEEE-------------EEE----------------EEE------------------EE--------------------------------------------------------------EE------------------EEE------------------EEEE---------------------------------------------------EEEE------EEE----------------------------EEE----------------------------------HHHHHHHH--------HHHHHHHHHH---------------------------EE----HHHHHHHHHHH--HHHHHHH-------------HHHHHHHHHHH---HHHHHHHHH---------E---------EEEHHHHHHHHHH---HHHHHHHHHHHH-----HHHHHHHH-EEEE----------HH----------------- : Jnet

jhmm : --HHHHHHHHH-------EEEE---HHHHH---------------------------------EE------EE------EEE----------------------EEEEE--------------EE------------------EE-----------------EEE---------------------------------------------------------EEE--EE----------EE------EEE-------------------EE--------------------------------------EE-------------EEE------EEEE---HHHHH------------------HHHH-H--HHH---------------------------HHHHHHHH-------HHHHHHHH------------------------------EEE---HHHHHHHHHH----HH-EEEE------------HHHHHHHHH--------HHHHHH--------EE---------EEEEHHHHHHHHHH--HHHHHHHHHHHH-----HHHHHHHH-EEEE----------------------------- : jhmm

jpssm : --HHHHHHHH------HHHHHHHH--HEEE-----------E------------------------------------HHHHHH------------------------EEE---EE-------EEE----------------EEE----------EEE-----------------EE--------------------------------------------------EEE------------------E-------------------EEEEE-------EEE----------------------------------------EEE--------E----------------------------EEEE----------------------------------HHHHHHHH----------HHHHHHHH----HHHHHH-----------------------HHHHHHHHHHH--HHHHHHHH-----------HHHHHHHHHHHHHHHHHHHHHHHH-------------------HHHHHHHHHHHH-----HHHHHHHHHHH-----HHHHHHHHHHEEE---------HHHHH-----------E--- : jpssm

Lupas 14 : ---------------------------------------------------------------------------------------------------------------------------------------------------------------------------------------------------------------------------------------------------------------------------------------------------------------------------------------------------------------------------------------------------------------------------------------------------------------------------------------------------------------------------------------------------------------------------------------------------------------------------------- : Lupas 14

Lupas 21 : ---------------------------------------------------------------------------------------------------------------------------------------------------------------------------------------------------------------------------------------------------------------------------------------------------------------------------------------------------------------------------------------------------------------------------------------------------------------------------------------------------------------------------------------------------------------------------------------------------------------------------------- : Lupas 21

Lupas 28 : ---------------------------------------------------------------------------------------------------------------------------------------------------------------------------------------------------------------------------------------------------------------------------------------------------------------------------------------------------------------------------------------------------------------------------------------------------------------------------------------------------------------------------------------------------------------------------------------------------------------------------------- : Lupas 28

Jnet_25 : ---BB-BB--B-----BBBBBBB-B-BBB-BBBB-BBB--BB-BBBBB-B--B-B-B-BB--BB-B-----BB----BBBBBBBBBB-B-----B--BB-BBB-BBB--B-B----B------BB--BB----BB--B-BB--BB--B-------B--B---BBBB-B--BB--B--B--B-----B--B---B--B---BB--B-BBBB-----B------B--B-BBB--B-----B-B-----BBBB---B----B--B--B------B--B---BBBBB--B----B--B--BBBB-BB------BB---B--B-BBB-BBBBBB--BBBB-BB-BB-B-----------------B--BBBB---BB--------B-----B------B----B-----B--BB-------B--BB--B-B-BBB----BB--B--B---B--B---BBB-B-B-BBBBBBBBBB-B-B--BB-BB-B-B--B--BB--B---BB-BB--B-------B--B-------B--B-------B-BBBBBB-BBBB--BB-BB-BB--B--B----BB-BB---B-BB-----B------B---B--B-----B-B-- : Jnet_25

Jnet_5 : ------BB----------B-B-----B---B--B-------B--------------------------------------------------------------B-B--B------B------B---------BB--B--------------------------B---------B------------------B------------------------------------------------------B---------B-----------------------B-------------------------------------B--BBB--------B-------------------------------B-------------------------------------B---------------B--B----------B-------------------------BBBBBB--B-------BB-BB-----------------B------------------------------------B-B-B-BB---B------B--B---B-------B--BB------------------------------------- : Jnet_5

Jnet_0 : --------------------B----------------------------------------------------------------------------------------B----------------------------------------------------------------------------------------------------------------------------------------------------------------------------------------------------------------------B-----------------------------------------------------------------------------------------------------------------------------------------B-BB------------------------------------------------------------------------------------------------------------------------------------------------ : Jnet_0

Jnet Rel : 8368999874177763311210133213315777752356331466553564134225667771277777710544443110331467777777777776677711124134632116777624613677765356777763164367777776310136771112467777763223666666667777777777777777777777777777777777771016502046777667622777764200667776776535677773178303778772101367777667777777777777777772277766777776304106777532230677411127777777777767777751110337722266665456666666667777777765278888861267787701227877411267310001325777766566554432102763689999998703518834210244432245641223899999310330139998751578877500431477763331278999985318737899999987437742577777640122147887766400103024577777642278 : Jnet Rel

: 1---------11--------21--------31--------41--------51--------61--------71--------81--------91--------101-------111-------121-------131-------141-------151-------161-------171-------181-------191-------201-------211-------221-------231-------241-------251-------261-------271-------281-------291-------301-------311-------321-------331-------341-------351-------361-------371-------381-------391-------401-------411-------421-------431-------441-------451-------461-------471-------481-------491-------501-------511-------521-------531-------541-------551-------561-------571-------581-------591-------601------- :

> Schistosoma bovis (SBOVIS_1435.187)

Jnet Rel : 972000122102124677777766332122010288765311101112103367777777777666666665033677776331331466667677777765356777631013677765356777763111366777776412346777667667777763223677777775133157750333677776677777777777777777775133315777777667777764133667776765320257773188303778772164235777677777777777777777777777767777776330467777111123662103124677777777777777777011111111101777777777321136677777776412101237777777654566772277773652001321016764421100156777762289999999987413299988515788777765228999999854432728999999823357775010367777775110389999985318737899999987517843267666503311147787776511031123577777742278 : Jnet Rel
 : 1---------11--------21--------31--------41--------51--------61--------71--------81--------91--------101-------111-------121-------131-------141-------151-------161-------171-------181-------191-------201-------211-------221-------231-------241-------251-------261-------271-------281-------291-------301-------311-------321-------331-------341-------351-------361-------371-------381-------391-------401-------411-------421-------431-------441-------451-------461-------471-------481-------491-------501-------511-------521-------531-------541-------551-------561-------571-------581-------591------- :

OrigSeq : MYQTYQSVHSFDAHTKSTMAYMYNFKVFIEFLCLTIVWNSVIAVPLIFQSETLEGKTYPTVDTIAQMNNSNYTISSNENNETVTTTVSSIEGTGGVVEIQTETCDDPLKEFVSPVRGIPRCCRKCEPGNGMLRLCSNAEDTQCRPCKPGFEFSPFRSATKKCLHCRRCEEIHPLAKTRNECTPITDTICQCEKPYYMSEKEQTCKPCTVCKPGEGIVQACGWNSDTQCQSCPAGFWSAQSIDNVKCIPCQSCGKDQVLVKQCSPTSDTLCCPLNNPNCTHELSMYFDYSAYDQESDISDNNNKSNQMLPIYCSIMGLIIISLLCYVVYKLWRQREASKNAKLTDSYNSNKTDLLDRTSCLDNNHLQHRRISSGFINNNNNAHLPNHSPQSTTELNNTINSTPDNDINNHLQFNDIIVGHEKAPLLGKLDHSNSSFSNFEQKPISVIPMNILGVICYRLSQHGWQELANIMDLETSKFDQLPSEVTSDLLSAAMEAQNTVESHLKLCNQDNSNTIQSITNNNPKNNLTMTVSMFQYMCLQNTVNLGQLMNSLQKLNRSDLVALIQQHTGIIKSKKSINHSNEEYKDKTKSMKSKENFQIEN : OrigSeq

Jnet : ----HHHHHHH--------------------------------HHHHHH--------------------------------------------------------------------------------------------------------------------------------------------------------------------------------------------------------------EEEE--------EEEE------------------------------------------------E--------------------------------HHHHHHHHHH-----------------------------H----------------------------HHHHHHH---------HH---------HHHHHHHHHHHHH--HHHHHHH------------HHHHHHHHHHHHHHHHHHHHHHHHH-------EE----------EEHHHHHHHHHHH---HHHHHHHHHHHHH----HHHHHHH---EEE------------HHH-------------- : Jnet

jhmm : --------EE---------------------EEE------------------------------------------------------------------------------------------------------------------------------------------------------------------------------------------------------------------------------EE----------EEE-----------------------------------------------EE-------------------------------HHHHHHHHHHHH---------HHH----------------HHHH---------------EE----H---HHHHHHHH------HHHHH---------HHHHHHHHHHHH---HHHHHH-------------HHHHHHHHHHHH-H-HHHHHHHH--------EEEE--------EEEEHHHHHHHHHH--HHHHHHHHHHHHH----HHHHHHH---EEE------------HHH-------------- : jhmm

jpssm : --HHHHHHHHHH-------------HHHHH---------HHHHHHHHHHHH----------------------E--------E---------------------------EEE----------------EEE-----------EE-----------------EE-----------EE------EE-----------------------------EEE------------------E----------EE-------EEEEE-------EEE----------------------------------------E---------EEE------E---------------------------------------------E-------------HH-----------------------------HHHHH----------------------HHHHHHHHHHHHH-HHHHHHHH------------HHHHHHHHHH-HHHHHHHHHHHHHHH------------------HHHHHHHHHHHH-----HHHHHHHHHHH-----HHHHHHHHHHEEE------------HHH----------E--- : jpssm

Lupas 14 : ---------------------------------------------------------------------------------------------------------------------------------------------------------------------------------------------------------------------------------------------------------------------------------------------------------------------------------------------------------------------------------------------------------------------------------------------------------------------------------------------------------------------------------------------------------------CCCCCCCCCCCCCC------------------------------------------- : Lupas 14

Lupas 21 : ------------------------------------------------------------------------------------------------------------------------------------------------------------------------------------------------------------------------------------------------------------------------------------------------------------------------------------------------------------------------------------------------------------------------------------------------------------------------------------------------------------------------------------------------------------------------------------------------------------------------ : Lupas 21

Lupas 28 : ------------------------------------------------------------------------------------------------------------------------------------------------------------------------------------------------------------------------------------------------------------------------------------------------------------------------------------------------------------------------------------------------------------------------------------------------------------------------------------------------------------------------------------------------------------------------------------------------------------------------ : Lupas 28

Jnet_25 : -B--B-BBB-B------B--BB--BBBB---BBBBB---BB-BB-BBBBBBBB---BBBBB-BBB-BB-B-BBBBB---B-BBBBBBB-BBBB-BBB-B----B--B---BBB---B---BB--B--B-BBB--B--B----BBBB-----BB-B--BB--BB-B--BB-BB-B-BB-B-BBBB-BBB-BB--B-BBB--B--BB-B--B--BBBB---B------B-B-B--BBBB--------B--B--B-----BB--B---BBBBBB-B----B--B--BBBBBBBB---B-BB-------BBBBBBBB-B-B-BBBB-B---B-------------B--------B--BB--B------------------------B-----------------BB---B---B-BB-B-B-B----BB-BB------B--B----BB-----BBBBBBBBB--B-B--BB-BB-B----B--B---BB--BB-BB--B---B--BB-BB-------B--B--------B-BBBBBB-BBBB--BB-BB-BB--B--B----BBBBB--BB-BB-----B------B---B--B-----B-B-- : Jnet_25

Jnet_5 : -------B--B-------------B-------B-------------BB------------------B----B-BB------BB-B------------------B------B-------------B--------------------------B---------B------------------B----------------------------------------------------------------B----------------------BB---------------B---------------------B--B--------------------------------------------------------------------------------------------------B-------------B-------------------------BB-BBB--B-------B---B-------------B---B-------------------------------------B---B-BB---B------B--B---B-------B--BB------------------------------------- : Jnet_5

Jnet_0 : -----------------------------------------------------------------------------------------------------------------------------------------------------------------------------------------------------------------------------------------------------------------------------B---------------------------------------------------------------------------------------------------------------------------------------------------------------------------------------BB------------------------------------------------------------------------------------------------------------------------------------------------- : Jnet_0

Jnet Rel : 972000122102124677777766332122010288765311101112103367777777777666666665033677776331331466667677777765356777631013677765356777763111366777776412346777667667777763223677777775133157750333677776677777777777777777775133315777777667777764133667776765320257773188303778772164235777677777777777777777777777767777776330467777111123662103124677777777777777777011111111101777777777321136677777776412101237777777654566772277773652001321016764421100156777762289999999987413299988515788777765228999999854432728999999823357775010367777775110389999985318737899999987517843267666503311147787776511031123577777742278 : Jnet Rel

: 1---------11--------21--------31--------41--------51--------61--------71--------81--------91--------101-------111-------121-------131-------141-------151-------161-------171-------181-------191-------201-------211-------221-------231-------241-------251-------261-------271-------281-------291-------301-------311-------321-------331-------341-------351-------361-------371-------381-------391-------401-------411-------421-------431-------441-------451-------461-------471-------481-------491-------501-------511-------521-------531-------541-------551-------561-------571-------581-------591------- :

> Opisthorchis_felineus ISOFORM 1 - CRM22_004569.1

Jnet Rel : 898887777654223567777624644332156777776643336777406505778776521001111357766677777777776666514678875899999999999999987325776232231577875300477762001001157777777777767666211232231177764013301202455677763300123765614513234565521437888862367776667777762289999999999999999988210578876278999999999860687078999999875278851123330036777753435677777889 : Jnet Rel

: 1---------11--------21--------31--------41--------51--------61--------71--------81--------91--------101-------111-------121-------131-------141-------151-------161-------171-------181-------191-------201-------211-------221-------231-------241-------251-------261-------271-------281-------291-------301-------311-------321-------331-------34 :

OrigSeq : MHLSTPATSPLPSALPKTGNETKTVSLTTMTPVETQPPADDIWMDGWPKKLTINGSSFYPPVQTAFHIGDYTRYEQNGEASDTSSKQNQMLPIYCSIMGFIIVFLLLYVVYKLWKQREAMTNAKLCEVYTSSGYSTVKLPVNSAQMDSVLNGPCDGDGGGAADHVKSENISRLTTKHLCGANRQQERDPLIANFETGANELSYLEIQLVTLQRDVLGMICFQLSRSGWREMATNMDIPTTSLLGPSANDSEFATQLAQAAQEAKHLILKESTTQNSPDEDTIKASARLLAKLCQQPTANVRVLLTELERINRGDIVAFISDKLTSSPAIPISPWSATKDPRV : OrigSeq

Jnet : -----------------------EEEEEEE-------------------EEEE----------HHEEE-----------------------HHHHHHHHHHHHHHHHHHHHHHHHHHHH------------------E--------HH-----------------------HHHHHH------HHHHH-HH-----------HHEEEEEEE----HHHHHHHHHHHHHHHHHHH---------------HHHHHHHHHHHHHHHHHHHHHH--------HHHHHHHHHHHHHH-----HHHHHHHHHHH-----HHHHHHH--------------------- : Jnet

jhmm : -----------------------EEEEEEE--------------------EEE------------EE------------------------HHHHHHHHHHHHHHHHHHHHHHHHHHHH-------------------------------------------------HHHHHHHHHH-----HHHH--HH---------HHHHHHHEEEE----HHHHHHHHHHHHHHHHHHH----------------HHHHHHHHHHHHHHHHHHHH---------HHHHHHHHHHHHHH----HHHHHHHHHHHH-----HHHHHH---------------------- : jhmm

jpssm : -----------------------EEE---------------EE------EEEE---------HHHHEEEE---------------------HHHHHHHHHHHHHHHHHHHHHHHHHHH------HHEE-------EEE-------HHHHH-----------------------EEE--------HHHHHHH------------EEEEEEEEEE--HHHHHHHHHH--HHHHHHH---------------HHHHHHHHHHHHHHHHHHHHHHH--------HHHHHHHHHHHHH-----HHHHHHHHHHH-----HHHHHHHH-------------------- : jpssm

Lupas 14 : --------------------------------------------------------------------------------------------------------------------------------------------------------------------------------------------------------cccccccccccccc-------------------------------------------------------------------------------------------------------------------------------- : Lupas 14

Lupas 21 : ------------------------------------------------------------------------------------------------------------------------------------------------------------------------------------------------------------------------------------------------------------------------------------------------------------------------------------------------------ : Lupas 21

Lupas 28 : ------------------------------------------------------------------------------------------------------------------------------------------------------------------------------------------------------------------------------------------------------------------------------------------------------------------------------------------------------ : Lupas 28

Jnet_25 : ----------BB-BBB-------BB-B-BBBBB--------BBB--B---B-B-B--BBBBB-BBBBBB-BB-B-----B---------BBBBBBBBBBBBBBBBBBBBBB-BB--------B-BB-BB---BB--B-BB-----B---B--BB---------------B--BBB-BBB---------BBBB-B--B---B--B---BB-B-B-BBBBBBB-B---BB--BB--B-B-BB-B-B-------BB--BB-BB--B--BB---B---------BB-BBB-BBB-BB---BB-B--BB--B--B----BBBBB---B-----BBB--B-------- : Jnet_25

Jnet_5 : ----------B------------------------------B--------B----------B-----B----------------------B-B-B-------B---------B-----------B------------------------B----------------------BB---------------B-----------------B--B---BBBBBB--B-------B---B----------------B---B-------------------------B---B-BB--BB------B--BB--B-------BB-BB-----------B----------- : Jnet_5

Jnet_0 : ------------------------------------------------------------------------------------------------------------------------------------------------------------------------------------------------------------------------B-BB-------------------------------------------------------------------------------B--B---------------B----------------------- : Jnet_0

Jnet Rel : 898887777654223567777624644332156777776643336777406505778776521001111357766677777777776666514678875899999999999999987325776232231577875300477762001001157777777777767666211232231177764013301202455677763300123765614513234565521437888862367776667777762289999999999999999988210578876278999999999860687078999999875278851123330036777753435677777889 : Jnet Rel

: 1---------11--------21--------31--------41--------51--------61--------71--------81--------91--------101-------111-------121-------131-------141-------151-------161-------171-------181-------191-------201-------211-------221-------231-------241-------251-------261-------271-------281-------291-------301-------311-------321-------331-------34 :

> Opisthorchis_felineus ISOFORM 2 - CRM22_004569.2

Jnet Rel : 9988777776650211213550234677766777777777777775223477731001367777666777774310126777630366777631001267777643113677633046777777643044412335650222333316776212046777667777654435677777652256777765115677742213467777663333377731883037888721771367777667777777777777777777677777775234664221122346541316761557664157777777777775216436777666777777777777777720167777632188776512332177775278888753775077875207178999999852550334875157777777777764201111123986243036765126778770378999999999810687318999998540188731899932116787632335677777889 : Jnet Rel
 : 1---------11--------21--------31--------41--------51--------61--------71--------81--------91--------101-------111-------121-------131-------141-------151-------161-------171-------181-------191-------201-------211-------221-------231-------241-------251-------261-------271-------281-------291-------301-------311-------321-------331-------341-------351-------361-------371-------381-------391-------401-------411-------421-------431-------441-------451-------461-------471-------481-------491-------501-------511-------521 :

OrigSeq : MKSGDMNSNKLAFVLSCLLALAYASPVNNHSSRESLNGTEDAPKKVQYVNVGNKTVVTEFSEQHLETCPGPMEEFVSPVRGSPRCCRMCGPGTGMLRLCTDADDTQCIGCEPGVEFSPTTSATLKCQQCRRCQDIHPLATTRIVCTPTTDTECGCMKGYYMSVNNQTCKACTVCKPNEGVIRPCEWNADTQCQACPAGFWSASVGDTVKCIPCKTCAENEVMVRNCRENEDALCCPKTNVNCTLSPVFGFDYTRYEQNGEASDTSSKQNQMLPIYCSIMGFIIVFLLLYVVYKLWKQREAMTNAKLCEVYTSSGYSTVKLPVNSAQMDSVLNGPCDGDGGGAADHVKSENISRLTTKHLCGANRQQERDPLIANFETGANELSYLEIQLVTLQRDVLGMICFQLSRSGWREMATNMDIPTTSLLGPSANDSEFATQLAQAAQEAKHLILKESTTQNSPDEDTIKASARLLAKLCQQPTANVRVLLTELERINRGDIVAFISDKLTSSPAIPISPWSATKDPRV : OrigSeq

Jnet : -------------------------------------------------------EE------------------E------------------EE-------------------------------------------------------------------------------------------------------EE------------------EEEE--------EEEE------------------------------------------------------------HHHHHH----------------EEE--------------------------HHHHHHHHH-------HHHHH-------HHHHHHH----HHHHHHHH--HHHHHHHHHHH--HHHHHHH----------------HHHHHHHHHHHHHHHHHHHHH-------HHHHHHHHHHHHHHH-----HHHHHHHHHHH-----HHHHHHHH-------------------- : Jnet

jhmm : ----------------------------------------------------------------------------------------------------------------------------------------------------------------------------------------------------------------------------EE----------EE-------------------------------------------------------------HHHHHH-----------------EE------------------------HHHHHHHHHHHH------HHHHHH------HHHHHHH-----HHHHHHH--HHHHHHHHHHH------HHH------------------------HHHHHHHHHHHHH-------HHHHHHHHHHHHHH-------HHHHHHHHHHH-----HHHH----------------------- : jhmm

jpssm : -------------EEEE-----EE-----------------------EE-----EEEE---------------EEEE----------------EEEE--------EEE------E-----------E------------EEEEEEE-------E--------------------------------------------EEEE---------EEE-----EEEEE-------EEEE------------------------------------------HHHHHHH-----E-----HHHHHH---------------EEEE---------------------------HHHHHHH--------------------HHHHHHH----HHHHHHH---HHHHHHHHHH---HHHHHHH---------------HHHHHHHHHHHHH--HHHHHHH---------HHHHHHHHHHHHH----HHHHHHHHHHHH-----HHHHHHHH-------------------- : jpssm

Lupas 14 : ---------------------------------------------------------------------------------------------------------------------------------------------------------------------------------------------------------------------------------------------------------------------------------------------------------------------------------------------------------------------------------------------cccccccccccccc-------------------------------------------------------------------------------------------------------------------------------- : Lupas 14

Lupas 21 : ------------------------------------------------------------------------------------------------------------------------------------------------------------------------------------------------------------------------------------------------------------------------------------------------------------------------------------------------------------------------------------------------------------------------------------------------------------------------------------------------------------------------------------------- : Lupas 21

Lupas 28 : ------------------------------------------------------------------------------------------------------------------------------------------------------------------------------------------------------------------------------------------------------------------------------------------------------------------------------------------------------------------------------------------------------------------------------------------------------------------------------------------------------------------------------------------- : Lupas 28

Jnet_25 : -------B--BBBBBBBBB-BBB-BBBB-BBB--BB-BB--B--BB-BB-B-B-BBBBBB----B-BB--B--BB-B-------BB--B--B-BB---B--B--B-B-BB--BB-BB-----B--BBBBB-BB-B---------B--B--BBBBBB-BBBBBB-B--BB-B--BBBB-BBBBBBB---BBBB--B--BBB---------B--B--B----BBB--B---BBBBBBB---B-BBBBB-BBBBBBBB-----B--B-B----BB---BBBBBBBBBBBBBB-BB-BB--B--BB-B-B-----B---BBBBBBB-BB---------B---BB--B-------B--BB------B--------B--BB--BB--B------B--BB--BB--BB--B--B-B--BB--B-B----B-B-------BB--BB-----B--BBB--B---------BB-BBB-BBB-BB----BBB-BBB--B--B----BB-BB---B-----B-B--B-------- : Jnet_25

Jnet_5 : -------------BBBB----B-----------------------B----------B----------B-----------------B--B-----------------B------------------B-------------------------------------------------------------------------B---------B-----------------------B-----------------------------------------B---BB-B--B--------B---------------------BB-B------------------------------B--B----------------------------------B--B----B---B-----------B-------------------------------------------------B--B--BB----------B--BB--B-------B---B-----------B----------- : Jnet_5

Jnet_0 : --------------B---------------------------------------------------------------------------------------------------------------------------------------------------------------------------------------------------------------------------------------------------------------------------------------------------------------------------------------------------------------------------------------------------------------------------------------------------------------------B-----------B--B--------------------------------------- : Jnet_0

Jnet Rel : 9988777776650211213550234677766777777777777775223477731001367777666777774310126777630366777631001267777643113677633046777777643044412335650222333316776212046777667777654435677777652256777765115677742213467777663333377731883037888721771367777667777777777777777777677777775234664221122346541316761557664157777777777775216436777666777777777777777720167777632188776512332177775278888753775077875207178999999852550334875157777777777764201111123986243036765126778770378999999999810687318999998540188731899932116787632335677777889 : Jnet Rel

: 1---------11--------21--------31--------41--------51--------61--------71--------81--------91--------101-------111-------121-------131-------141-------151-------161-------171-------181-------191-------201-------211-------221-------231-------241-------251-------261-------271-------281-------291-------301-------311-------321-------331-------341-------351-------361-------371-------381-------391-------401-------411-------421-------431-------441-------451-------461-------471-------481-------491-------501-------511-------521 :

> Fasciola hepatica (Genoma: PRJEB25283)

Jnet Rel : 998542118999931110236301111033467777633146777767776535677731000347777762165367731123775144036777763577126888731010136777766776777775213377731888517787721642677776654566777677777777777777667777774353331899999999986010019999999874110001157777777763213465001666666667777777777777777777777777777643121110177777722777777777765322332344566652100122000333489999999810574688875157777666666667777777642299999999999986247888761308999999999862687018999999874377314665432678888899 : Jnet Rel
 : 1---------11--------21--------31--------41--------51--------61--------71--------81--------91--------101-------111-------121-------131-------141-------151-------161-------171-------181-------191-------201-------211-------221-------231-------241-------251-------261-------271-------281-------291-------301-------311-------321-------331-------341-------351-------361-------371-------381-------391-------401-------411-------421-------431-------441-------451-------461----- :

OrigSeq : MLQTNQFVLAWLLSVVFVLSRVISLPVEVESGKGENSVTTLLNVTNSTGEMEPHALHPLAKTRTPCTPTVNTECECVADFYWSLPGQTCKPCTKCKPNEGVVKPCTWNTDTQCQACPSGFWSATTEDSVKCVPCQTCTGDQVVVKECSLSSDTVCCPKTNQNCTITDAFAYEYAASDPRDAGSSSSGDVVTKQNQMLPIYCSIMGMIIISLLLYVIYKLWKQREAMANAKLSEVFANGTANGLDKALIRSPNVTIGSAAAASPPIGPVLVTVPEATTAIPTEGTYDQMHHHTQSPVSETHSHGRRSGRKVRDPHEVSTNSEKLPLISGAQTSLTDQDYMEKPLNSIPRNTLGFVCYSLAQNGWRDLAIATGLGSDHLDQLDQMKSDKRIQWPIPEDLFQAAQCAKSLESAGGVPPSEQDLMGAVLTVGKLMERPDMNVHTFCTALEKSTRLDLVSMLTKSNCPASNGS : OrigSeq

Jnet : -------HHHHHHHHHHH----EEEEE---------------------------------EE----------EEE-------------EEE--------EEEE--------EEE--------------------------EEEEEE------EEE----------------------------------------EEEEEHHHHHHHHHHHHHH---HHHHHHHHHHHH-HH--------------------EE------------------------------------------EEE---------------------------------------------HHHHHHHHHHHHHHH----HHHHHH------------------------HHHHHHHHHHHHHHHH----------HHHHHHHHHHHHH----HHHHHHHHHHH-----HHHHHH---------- : Jnet

jhmm : --------HHHHHEEEE--------------------------------------------------------EE-------------EE---------EEE---------------------------------------EEEEE-------EE----------------------------------------EEEEEEHHHHHHHHHHHH-----HHHHHHHHHH------------------------EEE----------------------------------------EEEEEE------EE--------------------------HHH----HHHHHHHHHHHHHHHH-----HHHHHH-------------------------HHHHHHHHHHHHHHH----------HHHHHHHHHHHHH-----HHHHHHHHHH-----HHHHHH---------- : jhmm

jpssm : ------HHHHHHHHHHHHH--EEEEEEEEE--------E--------------------EEEEE--------EEE-----EEE----EEEE--------EEEE-------EEEEE-------------------E-----EEEEE-------EEE----------------------------------------EEEE-HHHHHHHHHHHHHHHHHHHHHHHHHHHHHHHHHH------------EEE---------------------------------------------------------------------------HH------------------H--HHHHHHHHHHHH----HHHHHH-----------------------HHHHHHHHHHHHHHHHH--------HHHHHHHHHHHHHH-----HHHHHHHHHHH-----HHHHHH---------- : jpssm

Lupas 14 : ------------------------------------------------------------------------------------------------------------------------------------------------------------------------------------------------------------------------------------------------------------------------------------------------------------------------------------------------------------------------------------------------------------------------------------------------------------------------------------ : Lupas 14

Lupas 21 : ------------------------------------------------------------------------------------------------------------------------------------------------------------------------------------------------------------------------------------------------------------------------------------------------------------------------------------------------------------------------------------------------------------------------------------------------------------------------------------ : Lupas 21

Lupas 28 : ------------------------------------------------------------------------------------------------------------------------------------------------------------------------------------------------------------------------------------------------------------------------------------------------------------------------------------------------------------------------------------------------------------------------------------------------------------------------------------ : Lupas 28

Jnet_25 : ------B-BBBBBBBBBBBB-BBBBBB-B-B--B---BB-BB--B--B--B-BB----B--B---B--B--B-B-B---BBB------B--B--B--BBBBB--B-----B-B--B--BBBB--------B--B--B----B-B--B---BBBBBB------B--B--B-B-BBBB-B--BB---B--BB---B-BBBBBB-BBBBBB-BBB-BBBB-BB-B--BB--B-BB-B-B---B--B--BBB-B--B-B-BB----------------BB--BB------BB-B--------B---B---B--B-----BB-----B-BB--B--BB--B--B--BB--B---BBBBBBBBBB---B--BB-BB-B----B--B--------B-B-BB--BB-BB--B--B--B---------B-BBB-BBB-BB----B-B--BB-BB--B----BBBBB----B------ : Jnet_25

Jnet_5 : ---------B--BB-BB-BB--B-B------------B---------B--B------------------------B------------B--B----------------------------B---------B-----------------------B-----------------B----------------------BB-B-B---BBBBB-B-------B---------------------------------------------------------------------------------------------------------BB-----------------------BBBBBB--B-------BB------------B----------------B----------------------B---B--B--B-------B--BB--B-------BB-BB----------- : Jnet_5

Jnet_0 : ---------------B---------------------------------------------------------------------------------------------------------------------------------------------------------------------------------------------B-B----------B------------------------------------------------------------------------------------------------------------------------------------B-BB-----------------------------------------B-------------------------------------------------------B--------------- : Jnet_0

Jnet Rel : 998542118999931110236301111033467777633146777767776535677731000347777762165367731123775144036777763577126888731010136777766776777775213377731888517787721642677776654566777677777777777777667777774353331899999999986010019999999874110001157777777763213465001666666667777777777777777777777777777643121110177777722777777777765322332344566652100122000333489999999810574688875157777666666667777777642299999999999986247888761308999999999862687018999999874377314665432678888899 : Jnet Rel

: 1---------11--------21--------31--------41--------51--------61--------71--------81--------91--------101-------111-------121-------131-------141-------151-------161-------171-------181-------191-------201-------211-------221-------231-------241-------251-------261-------271-------281-------291-------301-------311-------321-------331-------341-------351-------361-------371-------381-------391-------401-------411-------421-------431-------441-------451-------461----- :

> Taenia multiceps

Jnet Rel : 980011001233111467777777777777777777777777777777777777776667777631013467777631011377732123677777632236666656665121146777733304777763213467777777764310111100336777777777777777777777765356777731783037787721643677776677777777777777777777777777777666777774202421001101100100027651784332023677777777777777777665333563204677776677777777777777765228888213367877776521320307764247872034677777777733517899999982101305786038999999987526523266540201765116777777777776655556667677777889 : Jnet Rel
 : 1---------11--------21--------31--------41--------51--------61--------71--------81--------91--------101-------111-------121-------131-------141-------151-------161-------171-------181-------191-------201-------211-------221-------231-------241-------251-------261-------271-------281-------291-------301-------311-------321-------331-------341-------351-------361-------371-------381-------391-------401-------411-------421-------431-------441-------451-------461-------471- :

OrigSeq : MSILLLIWTLISSFWCSALPQALHQPLNSTQLDPIQQETCPSANEELVSTLDGPLRCCNKCPPGEGMLQLCTNQTQTVCRPCQEGSEFSLEASATAKCMQCKQCQELHPFAKFRQHCTPTSDAVCECVSGYFFIEALSTCQSCTKCPPGQGAEKPCGWNENTVCKPCAEGTWSSTDSATDICQTCRRCKPGQIEMRPCTATQNTLCCPLHNPNCEDTFEEEELDEPKSIQSQGKCTDDQAASQLAYGDDYPMITIYCSLLGLVILTLLIYVFYKLWQQRLSAEDAKTIEADVCYPAFTGLKSGRDKLRSSKISALSKDTTDRQHLLGGSQQYSYPPQNPTIHEALLTELSQGLAVENRWKHVGGLLGFNEESLQNFEKVGASDETSNPVDSAAVATRLMLTSWYSARAATDPNPLTSLLMVLERTPSTGHLCRCLKEYIKQSSTSSPYAMPPTQPPLQLPTSAISPEAQNTSNH : OrigSeq

Jnet : --HEHHHH----------------------------------------------------------------------EE---------------------------------------------------------------------HHHHHH------------------------------------EEEE--------EEE-----------------------------------------------EEEEEE-EE---E---EE-----HHHHHHH---------------------------------------------------------HHHHHH------------HHHHHH-----HHHHHH----------------HHHHHHHHHHHH--------HHHHHHHHHHHHH---HHHHHHHH--HHHHH-------------------------------- : Jnet

jhmm : --EEE-------------------------------------------------------------------------------------------------------------------------------------------------------------------------------------------EE----------EE-------------------------------------------------EEEEEEEEEEEEEEEEE---HHH---------------------------------------------------------------HHHH-------------HHHHHHH----HHHHH--------------EE-HHHHHHHHHH----------HHHHHHHHHHHHH---HHHHHHHH---HHHHH------------------------------- : jhmm

jpssm : --HHHHHHHHHH-----------------------------------------------------EEEE--------EEEE-----EEE---------EE----------------------EE--------EEE------------HHHHHHHHHH----------------------------------EEEEE-------EEE----------------------------------------------EEEE--------------------HHHHHHHH----------------------------E---------------------------HHHHHHHH-----------HHHH------HHHHHHHH---------------HHHHHHHHHHHHHH-----HHHHHHHHHHHHH-----HHHHHH-HHHHHH-------------------------------- : jpssm

Lupas 14 : ------------------------------------------------------------------------------------------------------------------------------------------------------------------------------------------------------------------------------------------------------------------------------------------------------------------------------------------------------------------------------------------------------------------------------------------------------------------------------------------ : Lupas 14

Lupas 21 : ------------------------------------------------------------------------------------------------------------------------------------------------------------------------------------------------------------------------------------------------------------------------------------------------------------------------------------------------------------------------------------------------------------------------------------------------------------------------------------------ : Lupas 21

Lupas 28 : ------------------------------------------------------------------------------------------------------------------------------------------------------------------------------------------------------------------------------------------------------------------------------------------------------------------------------------------------------------------------------------------------------------------------------------------------------------------------------------------ : Lupas 28

Jnet_25 : --BB-BBBBBBBBB-B--BB-BBBBB--B--B--B-B-BB--B---B------BB-BB--B--B-BBB--B-------BBBB--B--B------B--B--B--B--B--BBBBB--B--BBBBBBBB---BBBB-BB--BB-BBB----BBBB-BB-BBBB-----B--BBBBB-------B--B--B----B-B--B---BB-BB--B----B--B-----BB----B--B--B-----B--BBBBB---BBBBBBBBBBB---B--BB-BB-BB---B-------------------B------B--B-B-B----------B---B--B-B-------B--B-------------BBBBBB-B-B---BB--B-B---------BB--BB-BBBBBBBBBB---BBB---BB-BBB-BB--B----BBB-BB---BB-BBBB-B-B-------B-B---BB---B------ : Jnet_25

Jnet_5 : -----------B---B--B--------------------B-----------------B--B-----------------B--------B---------B-----------------------------------------------------------------------------------B-----------------------B-----------------------------------------------B--BBBBB-----------------------------------------------------------------------------------B-------------B--BB--B----------------------------BB-BBB-------------BB-BB--B---------B------------------------------------------- : Jnet_5

Jnet_0 : ----------------------------------------------------------------------------------------------------------------------------------------------------------------------------------------------------------------------------------------------------------------B---B-----------------------------------------------------------------------------------------------------B--------------------------------B--------------------BB-------------------------------------------------------- : Jnet_0

Jnet Rel : 980011001233111467777777777777777777777777777777777777776667777631013467777631011377732123677777632236666656665121146777733304777763213467777777764310111100336777777777777777777777765356777731783037787721643677776677777777777777777777777777777666777774202421001101100100027651784332023677777777777777777665333563204677776677777777777777765228888213367877776521320307764247872034677777777733517899999982101305786038999999987526523266540201765116777777777776655556667677777889 : Jnet Rel

: 1---------11--------21--------31--------41--------51--------61--------71--------81--------91--------101-------111-------121-------131-------141-------151-------161-------171-------181-------191-------201-------211-------221-------231-------241-------251-------261-------271-------281-------291-------301-------311-------321-------331-------341-------351-------361-------371-------381-------391-------401-------411-------421-------431-------441-------451-------461-------471- :

> Echinostoma caproni

Jnet Rel : 898877777777777777601205676513799875899999999999999998621576306621267777643335667777776667777731786157776665666677777777777666666667677765445411467777777777777666666677777654200010012322771023000014222278887115777777777777665556651078999999999998235888747899999999998626870189999987216872489999871599 : Jnet Rel
 : 1---------11--------21--------31--------41--------51--------61--------71--------81--------91--------101-------111-------121-------131-------141-------151-------161-------171-------181-------191-------201-------211-------221-------231-------241-------251-------261-------271-------281-------291------- :

OrigSeq : MCDYAASYPHDSDSSASGDVVIKQNQMLPIYCSIMGMIIISLLLYVVYKLWKQREAMASAKLSEVFANGTNGMDKALIRTPPPPGATNTTSGPSPTVVAVTEATHPSGMTNLGAVEGHHYDDELHHHHHAHSQCGTETRSHTKRSGHKARDSHTSTVPEKHPLIPNAKSSSTERDYLEQPVNVVVPQNTLGFVCYSLAQNGWRELAVTTGFNSTDLDQLDQLTSEQRTPWPLPDNLLQAARDAKSHESGAPHSELELTQAVLVVSKLLEQQDMTFGTFLTALGQSNRSDLVTMLASTNGS : OrigSeq

Jnet : --------------------EE------HHHHHHHHHHHHHHHHHHHHHHHHHHHH-----HHHHH-----------------------------EEEEE------------------------------------------EE-------------------------------HHHHHEEEEE----EEEEEHHHHHHHHHHHHHH-----------------------HHHHHHHHHHHHHHHH-------HHHHHHHHHHHHHHH----HHHHHHHHHHH----HHHHHHHH---- : Jnet

jhmm : -------------------EEEE-----HHHHHHHHHHHHHHHHHHHHHHHHHHHHH-----HH--------------------------------EEEE------------------------------------------EE----------------------------------EEEEEEE----EEEEEHHHHHHHHHHHHH-----------------------HHHHHHHHHHHHHHHH--------HHHHHHHHHHHHHHH-----HHHHHHHHHH----HHHHHHHH---- : jhmm

jpssm : ----------------------------HHHHHHHHHHHHHHHHHHHHHHHHHHHH----HHHHHH-----------------------------EEEEE---------------------------------------------------------------------------HHHHHHHHHH----HHHEHHHH----HHHHHHH------------------------HHHHHHHHHHHHHHHH------HHHHHHHHHHHHHH-----HHHHHHHHHH-----HHHHHHHHH--- : jpssm

Lupas 14 : ------------------------------------------------------------------------------------------------------------------------------------------------------------------------------------------------------------------------------------------------------------------------------------------------------------ : Lupas 14

Lupas 21 : ------------------------------------------------------------------------------------------------------------------------------------------------------------------------------------------------------------------------------------------------------------------------------------------------------------ : Lupas 21

Lupas 28 : ------------------------------------------------------------------------------------------------------------------------------------------------------------------------------------------------------------------------------------------------------------------------------------------------------------ : Lupas 28

Jnet_25 : -B-B-B-B-----B------------BBBBBBBBBBBBBBBBBBBBBB-BB----B---B-BB-BB---B-BB--BBB----------BB-B-B-B-BBB-------BBB-B--B----BB--B---B-----B------------B---------B----BBB--B------B-BB---B-BBB---BBBBBBB-BB--BB--BB--B-B-B--B--B--B-----B-B-BB--BB-BB--B------B------B--BB-BBB-BB----B-BB-BB--B-------BBBBB------ : Jnet_25

Jnet_5 : -------------------------------B---B---B------B--B-----------B-----------------------------------B----------------------------------------------------------------BB----------------B--------BBBBBB-B----B--B---------------------------------------------------B--BB-BB--B-------B--BB--B-------BB-BB------ : Jnet_5

Jnet_0 : -----------------------------------B----------------------------------------------------------------------------------------------------------------------------------------------------------B-BB-----------------------------------------------------------------------------------B-----------B---------- : Jnet_0

Jnet Rel : 898877777777777777601205676513799875899999999999999998621576306621267777643335667777776667777731786157776665666677777777777666666667677765445411467777777777777666666677777654200010012322771023000014222278887115777777777777665556651078999999999998235888747899999999998626870189999987216872489999871599 : Jnet Rel

: 1---------11--------21--------31--------41--------51--------61--------71--------81--------91--------101-------111-------121-------131-------141-------151-------161-------171-------181-------191-------201-------211-------221-------231-------241-------251-------261-------271-------281-------291------- :

> Schistocephalus solidus

Jnet Rel : 9852121313677877765278999886207884577614777877318886116723755136777766777777777776436899998647999999999999999863120201017777777664212022002257777777777652231114675133677777777652789999999986067401000013577115689988742110146777777777777643089999999931135788763289999998744774188886036777777777777777777777777777889 : Jnet Rel
 : 1---------11--------21--------31--------41--------51--------61--------71--------81--------91--------101-------111-------121-------131-------141-------151-------161-------171-------181-------191-------201-------211-------221-------231-------241-------251-------261-------271-------281-------291-------301-------311 :

OrigSeq : MAPLERVLPVADLEGHANNSMEELSSTLAKSQSDWVVFSELGPSTDLNVTFQNMKIQTSFRLQDNPPESQPVDVSQKPSEDYPMITIYCSLLGLIIITLLLYVVYKLWQQHLVSGDFKVASNETPNRQGLFGGLADCNKLRKAHTDHAGETISEKDGLLMDDSSVSTYNNERPASSLPPYVLSRLAEEIVRNGQWKSIGSQLGFTEENLHEFEVAAAAAAAAAAPTTNAEPGKYEHTVDVNAAYHMLTTWVSRPGSKVGTLQQTVCQSLAAGCDTSLCRLFDPTFCPPAGWSPSESVGDGRGPPAYSPADVPM : OrigSeq

Jnet : --------------------HHHHHHHHHH----EEEEE--------EEEEEEEEEEEEEEE---------------------HHHHHHHHHHHHHHHHHHHHHHHHHHHHHHHH-HHH--------------HHHH-----------------------------------------HHHHHHHHHHHHH-----HHH-------HHHHHHHHHHHHHH-------------------HHHHHHHHHHH---------HHHHHHHHHHH----HHHHHHH-------------------------------- : Jnet

jhmm : --------------------HHHHHHHHHH----EEEEE---------EEEEEEEE--EEE----------------------HHHHHHHHHHHHHHHHHHHHHHHHHHHHHHHHHHHHH----------------------------------------------------------HHHHHHHHHHHHH---------------HHHHHHHHHHHHHH-------------------HHHHHHHHH------------HHHHHHHHHH-----HHHHH--------------------------------- : jhmm

jpssm : ---HHHHH------------HHHHHHHHH-----EEEEE--------EEEEEEEEEEEEEEE---------------------HHHHHHHHHHHHHHHHHHHHHHHHHHHHHH-------------------HHHHHHH------------------H------E-------------HHHHHHHHHHHHH----HHHHHHH----HHHHHHHHHHHHHH-----------------HHHHHHHHHHHHHHH-------HHHHHHHHHHH----HHHHHHH-------------------------------- : jpssm

Lupas 14 : ------------------------------------------------------------------------------------------------------------------------------------------------------------------------------------------------------------------------------------------------------------------------------------------------------------------------- : Lupas 14

Lupas 21 : ------------------------------------------------------------------------------------------------------------------------------------------------------------------------------------------------------------------------------------------------------------------------------------------------------------------------- : Lupas 21

Lupas 28 : ------------------------------------------------------------------------------------------------------------------------------------------------------------------------------------------------------------------------------------------------------------------------------------------------------------------------- : Lupas 28

Jnet_25 : ---B--BBBBB-B---B---B--B---B------BBBBB-BB----B-BBB--B-B-B-B-B--------BB-B---B---BBBBBBBB--BBB--BB-BBBBBB-BB---BB---B--B-------BBBBBBBB-BB----B----B---B---BBBBB--B--BBB----B---BB-BBB--BB--BB----B--BB--BBB----B--B--BBBB-----B---------B---BBB-BBBBBBBBBBBB------BB--BBB-BBBB-B---BB-BB--BBBBBBBBBB---------B---------- : Jnet_25

Jnet_5 : ---------B-------------B-----------BBB--------B-------------------------------------B-B-B-----B-B---------B----------------------BB-BBB-B---------------------B-----------------B---BB--B-----------------------B------------------------------B-BB--BB---B---------B---B-----------BB--B---B-----B---------------------- : Jnet_5

Jnet_0 : ------------------------------------------------------------------------------------B------------------------------------------------B--B-------------------------------------------B-------------------------------------------------------------B---------------------B-----------B------------------------------------ : Jnet_0

Jnet Rel : 9852121313677877765278999886207884577614777877318886116723755136777766777777777776436899998647999999999999999863120201017777777664212022002257777777777652231114675133677777777652789999999986067401000013577115689988742110146777777777777643089999999931135788763289999998744774188886036777777777777777777777777777889 : Jnet Rel

: 1---------11--------21--------31--------41--------51--------61--------71--------81--------91--------101-------111-------121-------131-------141-------151-------161-------171-------181-------191-------201-------211-------221-------231-------241-------251-------261-------271-------281-------291-------301-------311 :

> Spirometra erinaceieuropaei_1 (SPER_0001928601-mRNA-1)

Jnet Rel : 82899999999999999999999998886268733337777653355532677642324300467777777763146763474267778899 : Jnet Rel
 : 1---------11--------21--------31--------41--------51--------61--------71--------81--------91 :

OrigSeq : MITVYCSLLGLIIITLLLYIVYKLWQQHLVSGDFKVASNETSRSQRLFEGFTGCNKLHKAAEISRAHTDQSGETISEKDGLLVDDSSMSNCE : OrigSeq

Jnet : -HHHHHHHHHHHHHHHHHHHHHHHHHHHHH----EE--------HHHHH------HHHHHH-------------------EEE--------- : Jnet

jhmm : -HHHHHHHHHHHHHHHHHHHHHHHHHHHHH----EE--------HHHHH------HHHHHH-------------------EEE--------- : jhmm

Lupas 14 : -------------------------------------------------------------------------------------------- : Lupas 14

Lupas 21 : -------------------------------------------------------------------------------------------- : Lupas 21

Lupas 28 : -------------------------------------------------------------------------------------------- : Lupas 28

Jnet_25 : -----B---BB--B----B--B-BB--BBB-B-B-BB---------BB-BB-BB--BB-BB-B--B--------B----BBBB--------- : Jnet_25

Jnet_5 : -------------B---------------------------------------B-------------------------------------- : Jnet_5

Jnet_0 : -------------------------------------------------------------------------------------------- : Jnet_0

Jnet Rel :82899999999999999999999998886268733337777653355532677642324300467777777763146763474267778899 : Jnet Rel

: 1---------11--------21--------31--------41--------51--------61--------71--------81--------91 :

> Spirometra erinaceieuropaei_2 (SPER_0001928601-mRNA-1)

Jnet Rel :82899999999999999999999998886268733337777653355532677642324300467777777763146763474267778899 : Jnet Rel
 : 1---------11--------21--------31--------41--------51--------61--------71--------81--------91 :

OrigSeq : MITVYCSLLGLIIITLLLYIVYKLWQQHLVSGDFKVASNETSRSQRLFEGFTGCNKLHKAAEISRAHTDQSGETISEKDGLLVDDSSMSNCE : OrigSeq

Jnet : -HHHHHHHHHHHHHHHHHHHHHHHHHHHHH----EE--------HHHHH------HHHHHH-------------------EEE--------- : Jnet

jhmm : -HHHHHHHHHHHHHHHHHHHHHHHHHHHHH----EE--------HHHHH------HHHHHH-------------------EEE--------- : jhmm

Lupas 14 : -------------------------------------------------------------------------------------------- : Lupas 14

Lupas 21 : -------------------------------------------------------------------------------------------- : Lupas 21

Lupas 28 : -------------------------------------------------------------------------------------------- : Lupas 28

Jnet_25 : -----B---BB--B----B--B-BB--BBB-B-B-BB---------BB-BB-BB--BB-BB-B--B--------B----BBBB--------- : Jnet_25

Jnet_5 : -------------B---------------------------------------B-------------------------------------- : Jnet_5

Jnet_0 : -------------------------------------------------------------------------------------------- : Jnet_0

Jnet Rel : 82899999999999999999999998886268733337777653355532677642324300467777777763146763474267778899 : Jnet Rel

: 1---------11--------21--------31--------41--------51--------61--------71--------81--------91 :

Legends:

SSSSS – region of Transmembrane Domain

YYYYY – region of conserved Death Domain
