# Supplementary material for: The evolution of TNF signaling in platyhelminths suggests the cooptation of TNF receptor in the host-parasite interplay
Source: Parasit Vectors. 2020 Sep 25;13:491. doi: 10.1186/s13071-020-04370-1 (PMC7519573; doi:10.1186/s13071-020-04370-1)
Supplement: Supplementary file 12 — Additional file 12: Figure S5. Matrices of BLAST results obtained by the alignment of human TNF ligands and platyhelminths TNF ligands (see detailed description in the figure). [file 13071_2020_4370_MOESM12_ESM.pdf]

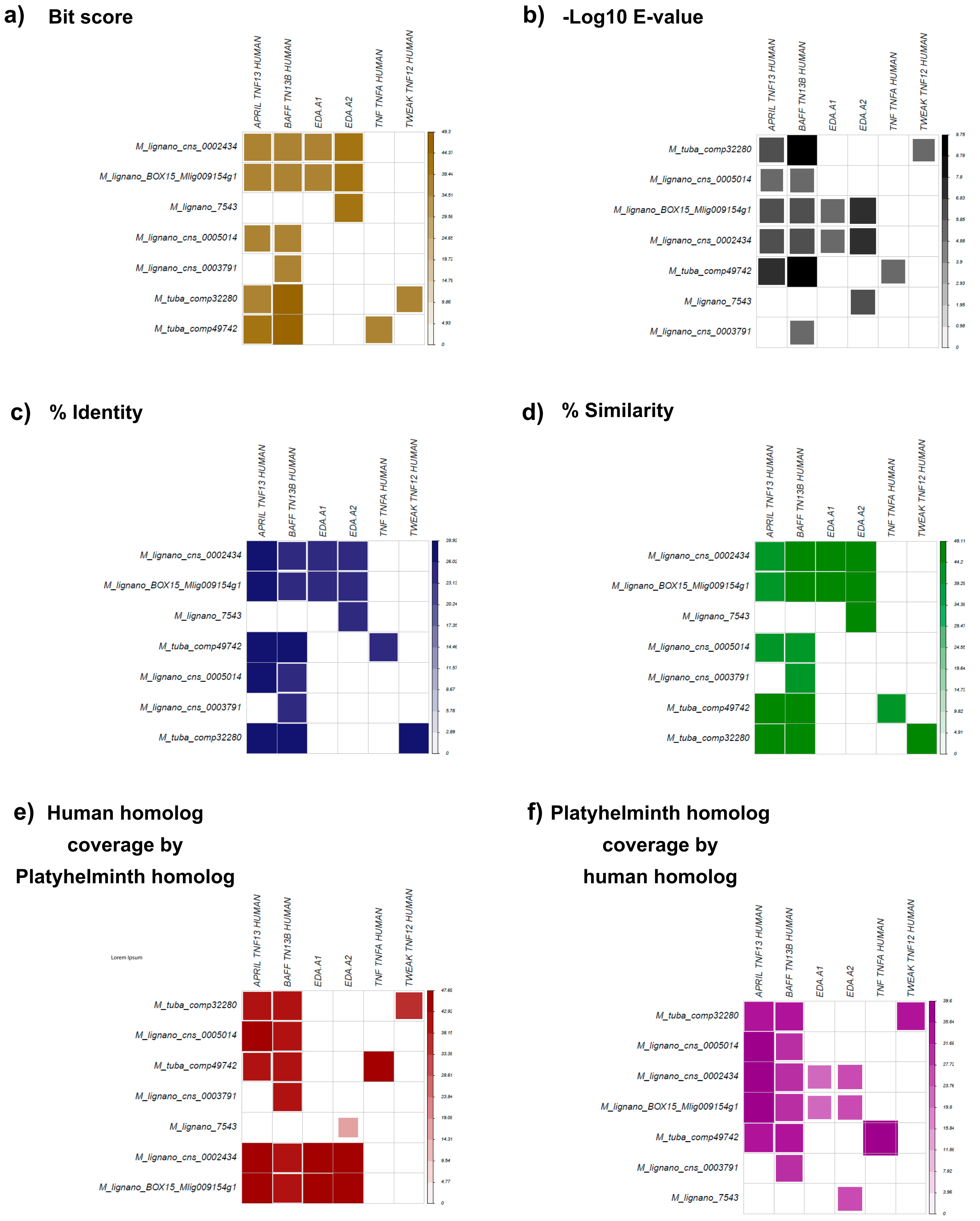

Additional file 12: Figure S5. Matrices of BLAST results obtained by the alignment of human TNF ligands and platyhelminths TNF ligands. a Alignment Bit scores; each score is represented by squares that are proportionally colored in beige (according to the intensity and colored area). b Alignment e-values; each e-value is represented by -log10 that is represented by squares which are inversely proportionally colored in black (according to the intensity and colored area), the lower P-value, the stronger the color. c Alignment identity percentages; each identity percentage is represented by squares that are proportionally colored in purple (according to the intensity and colored area). d Alignment positivity (similarity) percentages; each identity percentage is represented by squares that are proportionally colored in green (according to the intensity and colored area). e Alignment coverage percentage of human homologs by platyhelminth homologs, each coverage percentage is represented by squares that are proportionally colored in red (according to the intensity and colored area). f Alignment coverage percentage of platyhelminth homologs by human homologs, each coverage percentage is represented by squares that are proportionally colored in pink (according to the intensity and colored area). On the right of each figure is indicated the scale of color and its respective values.
