# Supplementary material for: The evolution of TNF signaling in platyhelminths suggests the cooptation of TNF receptor in the host-parasite interplay
Source: Parasit Vectors. 2020 Sep 25;13:491. doi: 10.1186/s13071-020-04370-1 (PMC7519573; doi:10.1186/s13071-020-04370-1)
Supplement: Supplementary file 14 — Additional file 14: Figure S6. Alignment of TNF domain of platyhelminth homologs (see detailed description in the figure). [file 13071_2020_4370_MOESM14_ESM.pdf]

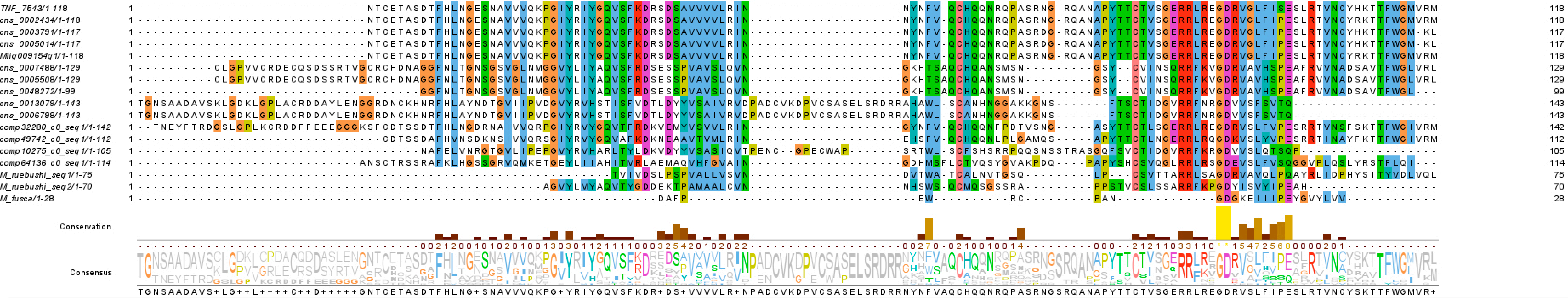

Additional file 14: Figure S6. Alignment of TNF domain of platyhelminth homologs. Alignment of 17 homolog sequences with TNF domain (around 120 amino acid residues) was performed by MUSCLE algorithm (MEGA 7.0 software). Amino acid residues were colored according to CLUSTAL pattern, at the bottom, the conservation level of each residues and consensus sequence are represented, as indicated.
