# Supplementary material for: The evolution of TNF signaling in platyhelminths suggests the cooptation of TNF receptor in the host-parasite interplay
Source: Parasit Vectors. 2020 Sep 25;13:491. doi: 10.1186/s13071-020-04370-1 (PMC7519573; doi:10.1186/s13071-020-04370-1)
Supplement: Supplementary file 2 — Additional file 2: Figure S1. Analysis of the quality parameters of the platyhelminth genome assemblies of all genomes used in this study, compared to the number of conserved TNFR domains that were identified in the original homolog gene predictions, according to each species. [file 13071_2020_4370_MOESM2_ESM.pdf]

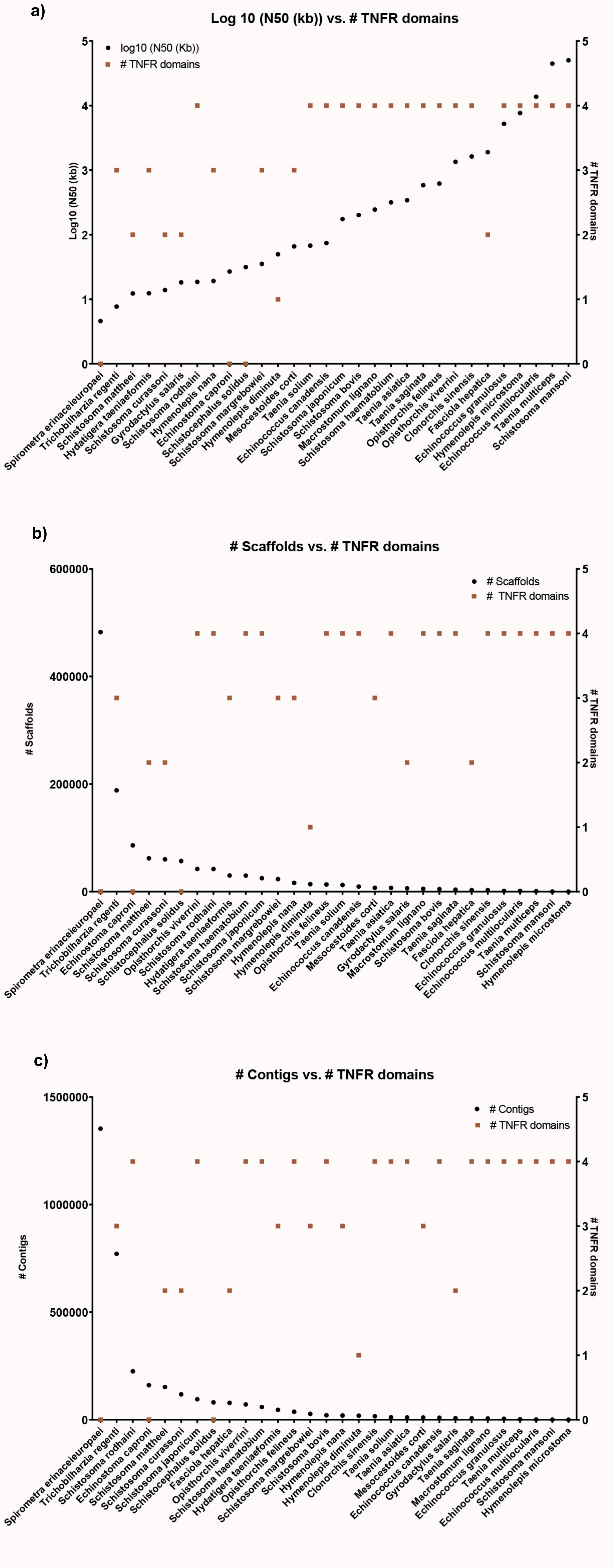

Additional file 2: Figure S1. Analysis of the quality parameters of the platyhelminth genome assemblies of all genomes used in this study, compared to the number of conserved TNFR domains that were identified in the original homolog gene predictions, according to each species. a Plot of log10(N50) (kb, left Y axis) and number of conserved identified TNFR domains (right Y axis). b Plot of the number of scaffolds (left Y axis) and number of conserved identified TNFR domain (right Y axis). c Plot of number of contigs (left Y axis) and number of conserved identified TNFR domain (right Y axis).
